# Genetic assignment predicts depth of benthic settlement for 0-group Atlantic cod

**DOI:** 10.1371/journal.pone.0292495

**Published:** 2023-10-04

**Authors:** Guðbjörg Ásta Ólafsdóttir, Shaun Turnbull, Ingibjörg G. Jónsdóttir, Anja Nickel, Hjalti Karlsson, Theresa Henke, Einar Eg Nielsen, Snæbjörn Pálsson

**Affiliations:** 1 University of Iceland, Research Centre of the Westfjords, Bolungarvík, Iceland; 2 Marine and Freshwater Research Institute, Hafnarfjörður, Iceland; 3 DTU Aqua, National Institute of Aquatic Resources, Silkeborg, Denmark; 4 University of Iceland, Faculty of Life and Environmental Sciences, Reykjavík, Iceland; Havforskningsinstituttet, NORWAY

## Abstract

Atlantic cod is a keystone species that remains among the most economically important demersal fish in the North Atlantic. Throughout its distribution range, Atlantic cod is composed of populations with varying environmental preferences and migratory propensities. This life-history variation is likely to have contributed to the niche width and large population sizes of Atlantic cod, and its relative resilience to environmental change and exploitation. The Icelandic cod stock is currently managed as a single unit, but early research indicates population variation by depth and temperature and distinct offshore and inshore spawning components. Pelagic 0-group juveniles from different spawning grounds coexist in nursery areas around Iceland, but their genetic composition or habitat partitioning had not been examined post benthic settlement. In the current study we examine the genetic composition of Atlantic cod juvenile aggregations at nearshore nursery grounds in NW-Iceland and report distinct segregation by the depth of offshore and inshore juvenile cod. The physiological mechanism of this segregation is not known, but the pattern demonstrates the need to consider population structure at nursery grounds in the application of marine spatial planning and other area-based conservation tools.

## Introduction

Atlantic cod is a keystone species that remains among the most economically important demersal fish in the North Atlantic despite severe overexploitation in many of the stocks [1]. Throughout its distribution range, Atlantic cod populations vary genetically across environmental gradients, such as temperature and salinity [2, 3]. Population variation in migratory propensity is also common [4], and was early on linked to genetic markers, notably the Pan I locus [5]. The Pan I locus has since been located on linkage group 1 (LG1) on the Atlantic cod genome, a supergene divergent between cod with different migratory propensity and life history [6]. Other linkage groups, or supergenes, have also been identified as important for the Atlantic cod population structure [6–9]. The origin of these supergenes is ancient [10], and variation in cod migratory behavior has been noted throughout historical times [11]. This life-history variation is likely to have contributed to the niche width and large population sizes of Atlantic cod, and its relative resilience to environmental change and exploitation.

The Icelandic cod has recovered in recent years, with reference stock biomass estimates exceeding 900 thousand tons since 2012 [12]. Currently managed as a single unit, stock structure of Atlantic cod in Icelandic waters has been examined for over two decades, first by identifying depth gradients in Pantophysin I (Pan I) and hemoglobin (HBI) frequencies [13] and soon after by confirming segregation by depth and geographic stock structure at the Pan I locus, microsatellites and by otolith shape and chemistry [14–16]. Later studies using genome examination of single-nucleotide polymorphism (SNPs) confirmed this differentiation and defined offshore and coastal populations based on a clustering analysis of spawning components around Iceland and Greenland [17].

Results from data storage tags (DSTs) show markedly different environmental profiles of Atlantic cod in Icelandic waters, part of the stock inhabits deeper and cooler waters both during foraging migrations and at spawning grounds [18, 19]. This environmental divergence is associated with genetic divergence on LG1 [20, 21]. The differentiation of stocks by foraging grounds [18], prompted the terms frontal and coastal cod [22], but the association of genetic stock structure and migratory behavior is likely complex [21] and there is no recent or geographically detailed genomic analysis of spawning populations of Atlantic cod around Iceland. In a study including contemporary and historical samples from Greenland, Iceland and Canada, Therkildsen et al. [17] sampled Icelandic cod from north and south coastal populations as well as at different depths at spawning grounds in south Iceland. Those results supported two genetic clusters associated with depth [17]. Further research is needed to resolve population structure of Icelandic cod and to understand how genetic structure relates to migratory behavior. However, in the current paper we make use of these previous population genetic clusters and refer to offshore or inshore populations as the identified groups by Therkildsen et al. [17]. We also use Pan I genotypes and refer to; Pan I^AA^, associated with cod in shallower, warmer waters, Pan I^BB^, associated with cod in offshore, cooler waters, and heterozygotes Pan I^AB^ [14]. When discussing Atlantic cod grouping based on depth or temperature profiles recorded by DSTs, we refer to migratory types [18, 19].

Pelagic juveniles from different spawning grounds coexist in nursery regions around Iceland [23, 24], but there is limited knowledge on juvenile distribution following benthic settlement. The main spawning area of Atlantic cod around Iceland is off the south-west coast, where migratory types have spawned at divergent depths [25]. Eggs and larvae from the main spawning ground are carried along the west and north coast with the Irminger current and then transported with coastal currents into fjords and nearshore waters, were they settle to benthic habitats and may mix with juveniles from local spawning components [23, 24, 26]. The abundance of pelagic juveniles found in these ocean currents varies year to year [27] but in any given year, more than half of the pelagic 0-group juveniles found in the northern area were likely to have originated from the main spawning area [24, 26, 28]. Tagging and recapture of age 1 and age 2 juvenile cod in the area has further shown that they remain mostly resident in their first two or three years [29].

Many questions remain unanswered on the stock complexities and population connectivity of Atlantic cod around Iceland. Resolving population structure and dynamics in the early life stages is a critical issue in stock management, for example, understanding how the influx of juveniles into nursery grounds and recruitment out of nursery grounds varies between different stock components, and how area-based conservation tool could be used to manage genetic variation. Moreover, smaller-scale patterns, caused by behavioral or physiological mechanisms post-settlement, can be important in determining growth and survival. Atlantic cod juveniles of different spawning stock origins co-occur in specific regions [30–32] but may use different habitats within those nursery grounds. A good example is the depth segregation of juvenile Norwegian coastal cod (NCC) and juvenile Northeast Arctic cod (NEAC) in near-shore waters off northern Norway, as the coastal cod juveniles were found in much shallower waters following benthic settlement [33]. Similar depth segregation of juveniles by ecotype was recently reported along the west coast of Sweden [34]. Conversely, in Skagerrak, coastal cod juveniles and juveniles from North Sea spawning populations inhabit nearshore nursery grounds without clear habitat or depth segregation [35, 36], although juvenile aggregations have differed temporally in genetic composition [37]. Different spawning time of ecotypes and spawning components could contribute to the segregation of juvenile cod ecotypes on nursery grounds.

The current study determined the genetic origin of Atlantic cod juvenile aggregations at nearshore nursery grounds in the Westfjords, NW-Iceland. The area is known as an important nursery area for cod with mixing of pelagic juveniles from different spawning grounds and genotypes [23, 28]. However, prior studies have not sampled 0-group juveniles post settlement or shallow tidal waters. For the current study, we repeatedly sampled juvenile cod in both shallow tidal waters and deep fjords around the Westfjords, analyzed variation in juvenile length and assigned juveniles to inshore and offshore populations using the specifically developed SNP panel described above [17]. We also determined size and Pan I alleles for a larger sample of individuals available as a measure of intra-annual variation. We ask if juvenile origin differs by sampling depth, indicating habitat segregation, or by time, indicating an influx of juveniles with different genetic makeup from different spatial or temporal spawning grounds.

## Materials and methods

### Ethics statement

The fish used for this research were either by-catch in a fishery survey conducted by the Marine and Freshwater Research Institute of Iceland (MFRI) or caught by beach seine for ecological field sampling. Neither is subject to licensing by an animal welfare or ethics committee by Icelandic law. The fish were anesthetized, immediately upon capture, by overexposure to phenoxyethanol to minimize stress or suffering.

### Study area and samples

**Fig 1:**
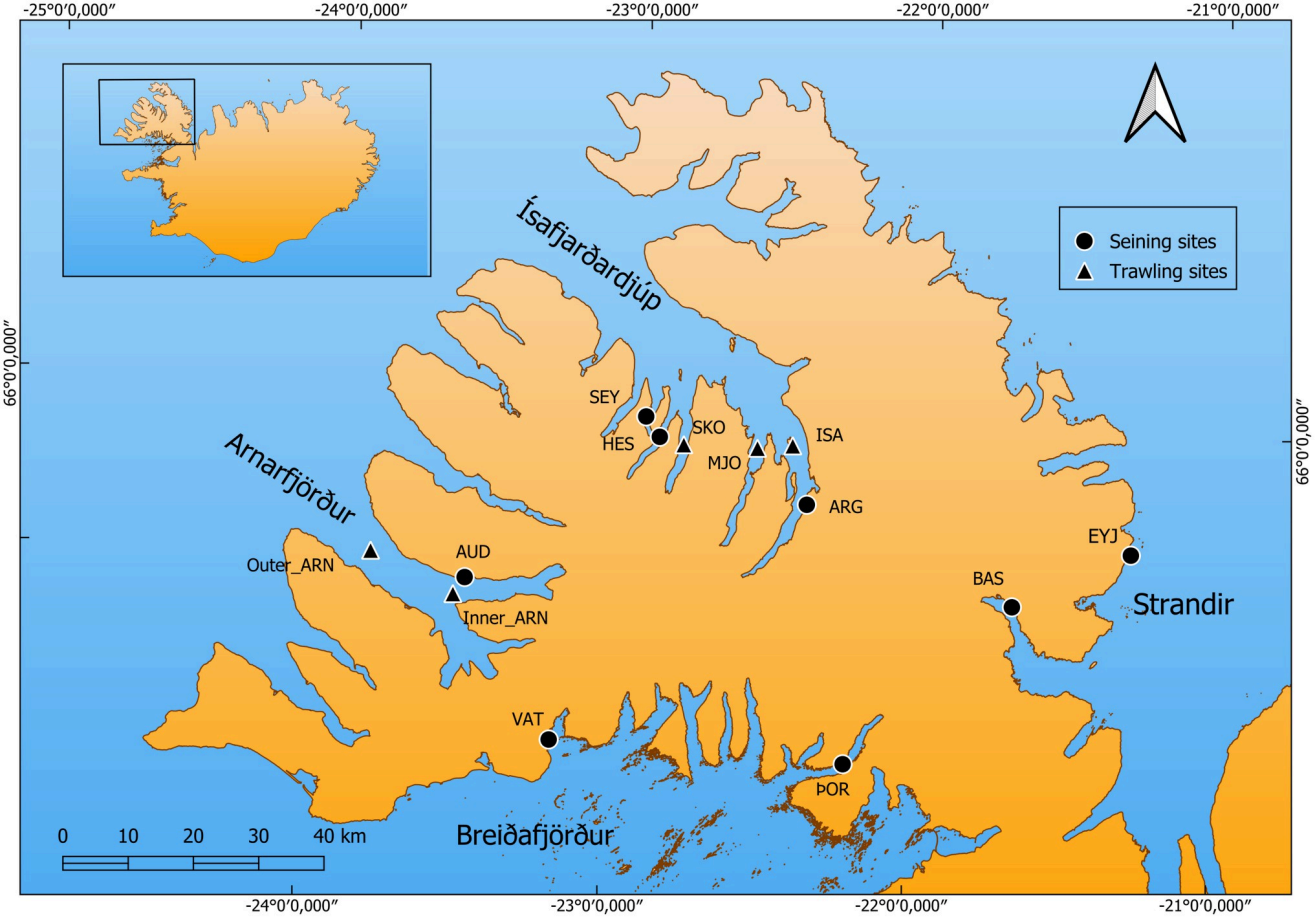
Map of sample sites. The map shows the four areas and the sample sites within each area (areas and site code matching Table 1). Beach seining sites are indicated by a black dot and trawl sites by a black triangle. The coastline presented in the map is based on the National Land Survey of Iceland (Landmælingar Íslands) IS 50V database, made available to the authors by CC BY 4.0, downloaded 12/2018 (www.lmi.is).

The juvenile cod used in this study were sampled around the Westfjords, a large peninsula in NW Iceland (Fig 1). Iceland is situated on the Greenland-Scotland ridge were the warm Irminger current from the south meets the cold currents from the north, resulting in a distinct temperature gradient between southern and northern Icelandic waters [38, 39]. The ocean front is located off the west of the Westfjords, making the peninsula an ideal system to examine biological variation across these ocean currents and the resulting temperature gradient. Juvenile cod were sampled with a beach seine (1.5 m x 20 m, mesh size = 6 mm) in tidal waters (depth < 1.5 m) in four areas (fjord systems), Breiðafjörður being the southernmost area, then Arnarfjörður, Ísafjarðardjúp and Strandir being the furthest north. The beach seining occurred at the time of benthic settlement of pelagic cod juveniles, in late August to early September, and again a few weeks post-settlement in 2017 and again in 2019, at all sites except for Breiðafjörður (Table 1). In October 2019, juvenile cod were specifically sampled for this study from by-catch in shrimp trawl (40 mm mesh size cod end) in an annual shrimp survey conducted by the Marine and Freshwater Research Institute (MFRI) in Ísafjarðardjúp and Arnarfjörður. As the numbers of juveniles caught as by-catch is highly variable among sampling stations juveniles were pooled from nearby stations to form four groups that represented distinct geographical variation, outer Arnarfjörður (Outer_ARN), inner Arnarfjörður (Inner_ARN), Skötufjörður (SKÖ) and Ísafjörður (ISA) (Fig 1). Additionally, samples from several trawl stations of the same survey carried out in Ísafjarðardjúp in November 2017 could be used and were pooled to represent two sample groups, Ísafjörður (ISA) and Mjófjörður (MJO). The trawl sites were at depths ranging from 33–93 meters. Catch from all sampling was frozen as soon as possible pending further analysis. Subsequently, the juveniles were defrosted, weighted, their standard length (SL) measured, and a fin clip taken for genetic analysis. Note that the juveniles caught in 2017 represent a post-hoc addition to core sampling of 2019 that was specifically for this study. This resulted in a somewhat unbalanced study design, as is reflected in the number of samples per group, number of samples per depth and the different timing of the trawl survey between the two years. Moreover, the SNP analysis could only be done on the 2019 samples. Nevertheless, we concluded that the inclusion of a second year was beneficial as it allowed examination of inter-annual variation.

**Table 1:** Overview of the juvenile cod samples used in the current study. The table shows the four sample areas as well as each sample site within that area, separated by time of sampling. The number of juvenile cod used for assignment to inshore / offshore populations is indicated as well as Pan I allele frequencies in each sample group and summary statistics for the SNP data.

Area	Site code	Age group	Month	Year	Gear	n SNPs	Inshore	Offshore	H_E_	H_O_	F_IS_	n Pan I	AA	AB	BB
Ísafjarðardjúp	ARG	Age 0	10	2017	Seine	-	-	-	-	-	-	32	7	21	4
Ísafjarðardjúp	ARG	Age 0	8	2017	Seine	-	-	-	-	-	-	31	24	7	0
Ísafjarðardjúp	ARG	Age 0	8	2019	Seine	14	14	0	0.23	0.21	0.11	14	9	5	0
Arnarfjörður	AUD	Age 0	10	2017	Seine	-	-	-	-	-	-	18	16	2	0
Arnarfjörður	AUD	Age 0	9	2017	Seine	-	-	-	-	-	-	22	21	1	0
Arnarfjörður	AUD	Age 0	9	2019	Seine	38	38	0	0.25	0.23	0.10	38	31	7	0
Arnarfjörður	AUD	Age 0	10	2019	Seine	28	28	0	0.24	0.23	0.04	27	23	4	0
Strandir	BAS	Age 0	10	2017	Seine	-	-	-	-	-	-	19	10	7	2
Strandir	BAS	Age 0	10	2019	Seine	35	31	4	0.24	0.22	0.14	34	28	5	1
Strandir	BAS	Age 0	8	2017	Seine	-	-	-	-	-	-	26	20	4	2
Strandir	BAS	Age 0	8	2019	Seine	35	32	3	0.25	0.22	0.10	62	52	9	1
Strandir	EYJ	Age 0	8	2017	Seine	-	-	-	-	-	-	11	2	8	1
Strandir	EYJ	Age 0	8	2019	Seine	-	-	-	-	-	-	42	39	3	0
Arnarfjörður	Inner_ARN	Age 0	10	2019	Trawl	31	18	13	0.22	0.19	0.16	49	2	13	34
Ísafjarðardjúp	ISA	Age 0	10	2019	Trawl	55	14	41	0.23	0.20	0.14	35	1	11	23
Ísafjarðardjúp	ISA	Age 0	11	2017	Trawl	-	-	-	-	-	-	127	9	45	73
Ísafjarðardjúp	ISA	Age 1	10	2019	Trawl	-	-	-	-	-	-	26	8	11	7
Ísafjarðardjúp	ISA	Age 1	11	2017	Trawl	-	-	-	-	-	-	13	0	6	7
Ísafjarðardjúp	MJO	Age 0	11	2017	Trawl	-	-	-	-	-	-	23	0	16	7
Ísafjarðardjúp	MJO	Age 1	11	2017	Trawl	-	-	-	-	-	-	15	1	10	4
Arnarfjörður	Outer_ARN	Age 0	10	2019	Trawl	39	7	32	0.19	0.16	0.18	39	1	9	29
Arnarfjörður	Outer_ARN	Age 1	10	2019	Trawl	-	-	-	-	-	-	21	6	13	2
Ísafjarðardjúp	SEY	Age 0	10	2019	Seine	34	31	3	0.24	0.22	0.10	36	27	6	3
Ísafjarðardjúp	SEY	Age 0	9	2019	Seine	45	40	5	0.25	0.23	0.11	34	28	5	1
Ísafjarðardjúp	SKO	Age 0	10	2019	Trawl	40	18	22	0.19	0.17	0.142	37	2	14	21
Breiðafjörður	VAT	Age 0	9	2017	Seine	-	-	-	-	-	-	9	2	6	1
Breiðafjörður	ÞOR	Age 0	10	2017	Seine	-	-	-	-	-	-	3	2	1	0
Breiðafjörður	ÞOR	Age 0	9	2017	Seine	-	-	-	-	-	-	40	26	12	2

After examining the length/frequency distributions, juveniles < 14 cm (SL) were assigned to age as 0-group juveniles and juveniles > 14 cm as 1-year-old juveniles. This cut-off may not fully differentiate between age classes in 2017 because the trawl survey was conducted in November. This makes differentiating age classes solely on size difficult and may result in some 0-group juveniles in 2017 being misclassified as 1-year-olds. However, this is preferable to false positive assignment of 1-year-olds as 0-groups, as the current analysis is focused on the early settling 0-group juveniles, and most of the sampling (before November) assigns well two the two size groups (S2 Fig). However, the size distributions presented should be interpreted keeping this uncertainty in mind. Only three putative 1-year-old juveniles were caught with the beach seine (and excluded from any further analysis), but the size (SL) and Pan I genotypes of the 77 putative 1-year-old juvenile cod from the trawl survey were used in the current study (Table 1). Note that these sample numbers do not reflect the relative frequencies of age classes in the catch as the collection of by-catch in the shrimp survey focused primarily on the 0-group. No fish larger than 23 cm was used in this study. The geographical areas, and sample groups within areas, as they are referred to in the statistical analysis can be found in Table 1 and are depicted in Fig 1.

### Genotyping

DNA was extracted using a Genomic DNA Purification Kit (Thermo Scientific) following the manufacturer’s protocol. Representative samples of geographical and depth variation from 2019 (n = 393), were analyzed using a 96 SNP panel previously developed to resolve the population structure of Atlantic cod around Iceland and Greenland (see Christensen et al. [40] for details of methods). A larger sample of 883 juvenile cod, from 2017 and 2019 was analyzed for the allele variation at the Pan I loci. Not all individuals genotyped at the Pan I locus could be analyzed using the 96 SNP panel and vice versa (see S1 Table for details). Pan I alleles A and B are distinguished by a single-nucleotide polymorphism (SNP) located on LG1, making the Pan locus potentially useful to differentiate between Atlantic cod inshore and offshore types [6]. Concurrence between SNP assignment and Pan I genotypes was examined when both were available for the same individuals (n = 346).

Samples were SNP genotyped using allele specific primers on a Fluidigms 96.96 Dynamic Array^™^ IFC. Individuals genotyped for less than 60 SNPs were discarded from further analysis (a total of 13 individuals). The Pan I alleles were determined by targeting variation of the specific Pan I SNP [6] using a KASP (Kompetitive Allele-Specific PCR) assay following the manufacturer’s protocol (LGC Genomics). The qPCR runs were replicated at least twice with a negative control for each eleven samples. The PCR reactions were run on a QuantStudio 3 Real-Time PCR system (Applied Biosystems), and the alleles called manually using the cloud-based Applied Biosystems analysis modules for genotyping.

### Statistical analysis

**Fig 2:**
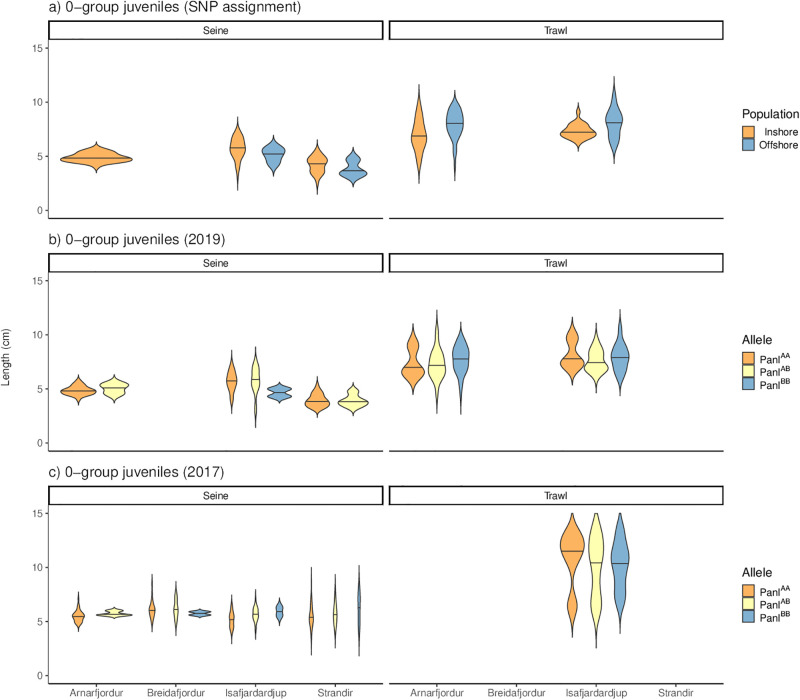
Standard length of juvenile Atlantic cod populations and genotypes across fishing gear and sample sites. The violin plots show the distribution of juvenile size in the areas sampled and by different fishing gear. The SNP assignment to inshore and offshore populations (a) and Pan I genotypes (b & c) are depicted separately and note that although the two sample years are depicted for the Pan I genotypes (b & c) the figure does not reflect variation in sampling time post benthic settlement.

The data used for analysis is available in S1 Table. Data handling, statistics, and graphs were done in R 4.1.0 software [41] and by using the R package tidyverse [42] in addition to the packages and software cited below. All models were checked using the R package DHARMa [43], and the examination revealed no significant deviations from the expected distribution of residuals. All the statistical analysis was done separately for the 0-group and 1-year-old juveniles, as well as for the inshore/offshore and Pan I dataset. To examine if juveniles of different origins (inshore/offshore assignment or Pan I genotypes) from different areas, sampling times or depths (as represented by beach seine and trawl) differed in size, we used a general linear model (GLM) with log-transformed standard length as the response variable, either inshore/offshore assignment or Pan I genotype (Pan I^AA^, Pan I^AB^ and Pan I^BB^), area (Breiðafjörður, Arnarfjörður, Ísafjarðardjúp, and Strandir), gear (trawl or beach seine), year and month as fixed effects. The model included an interaction effect between assignment/Pan I genotype and gear (acknowledging the obvious size difference between juveniles by gear) as well as the sampling area and month of sampling to examine temporal changes. The standard length of 0-group juveniles is depicted in Fig 2 and putative 1-year-old juveniles is S2 Fig.

Descriptive statistics, H_E_, H_O_ and F_IS_ were calculated for each sample group of the 96-SNP dataset using the Genetix 4.05 software [44]. Differentiation between samples representing sampling event or time was estimated by first calculating the pairwise F_ST_ values of all sample groups using the R package hierfstat version 0.5–10 [45]. Second, we used a discriminant analysis of principal components (DAPC), a non-model-based clustering method implemented in the R package adegenet 1.3–1 [46], to visualize the genetic composition of sample groups. We used the find.clusters function in adegenet to estimate the number of clusters in our sample and find.clusters used K-means clustering to maximize between group variance and minimizes within group variance. To select the optimal k, we applied the Bayesian Information Criterion (BIC) as suggested by the authors [46]. We then used DAPC on the pre-defined clusters with the dapc function in adegenet. Third, we identified the loci that had the highest loading on the axis of divergence between the identified clusters also in adegenet. Finally, we assigned the juveniles to the previously identified inshore/offshore populations originated from the SNP study of Atlantic cod population structure in Greenland and Iceland [17]. In that study 847 contemporary and historical cod tissue samples were analysed with 935 SNPs and individual genotypes clustered with DAPC [46] into four groups were two included the Icelandic samples [17].

Two binomial generalized linear models were used to examine how the likelihood of inshore vs offshore assignment and Pan I^AA^ vs PanI^BB^ genotypes varied across gear (trawl or beach seine), depth, area (Breiðafjörður, Arnarfjörður, Ísafjarðardjúp, and Strandir), and month of sampling. Note that gear and depth are highly correlated, but both are included in the model to examine if the juvenile genotype distribution would be better reflected by gear (indicating an abrupt shift) or depth (indicating a more gradual shift). For both models, Arnarfjordur was used as the reference (area) for the categorical factor of area.

## Results

The 0-group juveniles caught in trawls were much larger than the juveniles caught with a beach seine (Fig 2, Table 2). Moreover, 0-group juveniles were significantly smallest in Strandir, the sites furthest north. The 0-group juveniles were smaller in 2019 than in 2017 (Table 2). Juvenile standard length increased slightly by month of sampling. The standard length of juveniles assigned as offshore differed between trawl and seine samples (Table 2) but inshore/offshore assignment or Pan I genotype did not affect the standard length in other comparisons. The 1-year-old juveniles were larger in Arnarfjörður than in Ísafjarðardjúp and smaller in 2019 than in 2017 (Table 2). It should be noted that the difference in the time of the trawl survey between years could confound any between year as well as overall size comparison (S1 Fig).

**Table 2:** Results from the three generalized linear models examining variation in juvenile cod standard length (SL). The analysis was done separately for 0-group and putative 1-year-old juveniles as well as for the SNP and Pan I dataset. The results indicate differences in size by area, such as a north-south size gradient, as well as differences in sizes between years. The most notable result relating to genotype or population is that juveniles assigned to the offshore population are slightly (but significantly) larger that inshore juvenile within the trawled samples.

Predictors	0-group by Pan I genotypes			0-group by inshore /offshore populations			1-year-old		
	Estimate	CI	p-value	Estimate	CI	p-value	Estimate	CI	p-value
(Intercept)	62.62	46.27–78.97	**<0.001**	0.70	0.69–0.72	**<0.001**	-70.42	-100.21 –-40.62	**<0.001**
Pan genotype [AB]	0.01	-0.01–0.03	0.384	-	-	-	0.02	-0.01–0.05	0.186
Pan genotype [BB]	0.01	-0.04–0.05	0.787	-	-	-	0.03	-0.01–0.06	0.176
Gear [Trawl]	0.20	0.15–0.25	**<0.001**	0.13	0.10–0.16	**<0.001**	-	-	-
Area [Breiðafjörður]	-1.07	-2.14–0.00	0.051	-	-	-	-	-	-
Area [Ísafjarðardjúp]	-0.08	-0.35–0.20	0.595	0.03	0.01–0.05	**0.001**	-0.07	-0.10 –-0.03	**<0.001**
Area [Strandir]	-0.61	-0.95 –-0.27	**<0.001**	-0.09	-0.12 –-0.05	**<0.001**	-0.01	-0.09–0.07	0.777
Month	0.01	-0.02–0.04	0.538	-	-	-	-	-	-
Year	-0.03	-0.04 –-0.02	**<0.001**	-	-	-	0.04	0.02–0.05	**<0.001**
Pan genotype [AB] × Gear[Trawl]	-0.04	-0.08–0.01	0.128	-	-	-	-	-	-
Pan genotype [BB] × Gear[Trawl]	-0.02	-0.08–0.04	0.571	-	-	-	-	-	-
Area [Breiðafjörður] ×Month	0.12	0.00–0.24	**0.042**	-	-	-	-	-	-
Area [Ísafjarðardjúp] × Month	0.01	-0.02–0.04	0.457	-	-	-	-	-	-
Area [Strandir] × Month	0.07	0.03–0.10	**<0.001**	-	-	-	-	-	-
SNP assignment [Offshore]	-	-	-	-0.03	-0.09–0.02	0.230	-	-	-
SNP assignment [Offshore]× Gear [Trawl]	-	-	-	0.08	0.02–0.14	**0.009**	-	-	-
Observations	692			253			77		
R^2^ / R^2^ adjusted	0.751 / 0.746			0.683 / 0.677			0.522 / 0.488		

Only 0-group juveniles were genotyped with the 96-SNP panel. There were no instances of heterozygote deficiency or significant F_IS_ values within sample groups (Table 1). Pairwise F_ST_ values calculated using the 96-SNP dataset were most often significant between juvenile groups sampled with a trawl at more depth and juvenile groups sampled with a beach seine (S2 Table).

**Fig 3:**
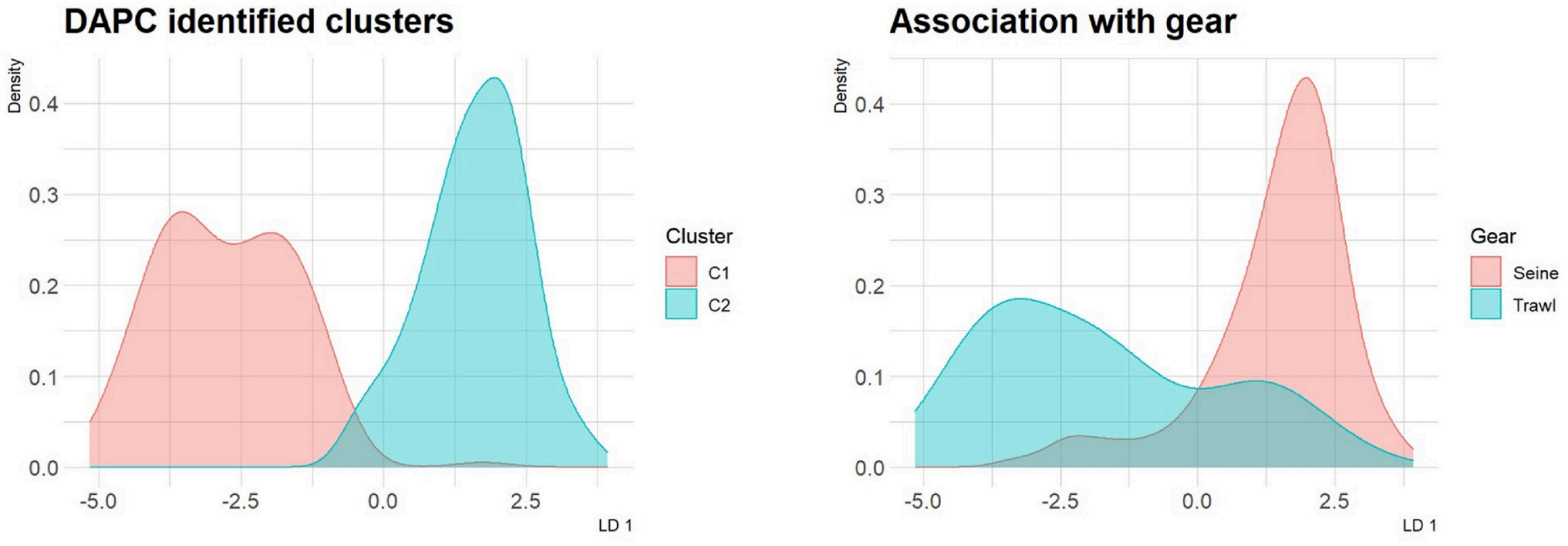
Results from the DAPC analysis. The figure shows how the juveniles separated to the two identified clusters based on the SNP data and how the identified groups association to fishing gear.

The largest drop in BIC values was observed between one and two clusters, and the values then plateaued. The clear clusters (Fig 3) support that juveniles of two population components were present and that the previously identified differentiation between inshore and offshore populations [17] explained much of the variation in the SNP data. Three SNPs were found to have the highest loadings on the axis discriminating between groups: Rhodopsin, cgpGmo-S1166, and cgpGmo-S523. These loci are all on LG1.

**Fig 4:**
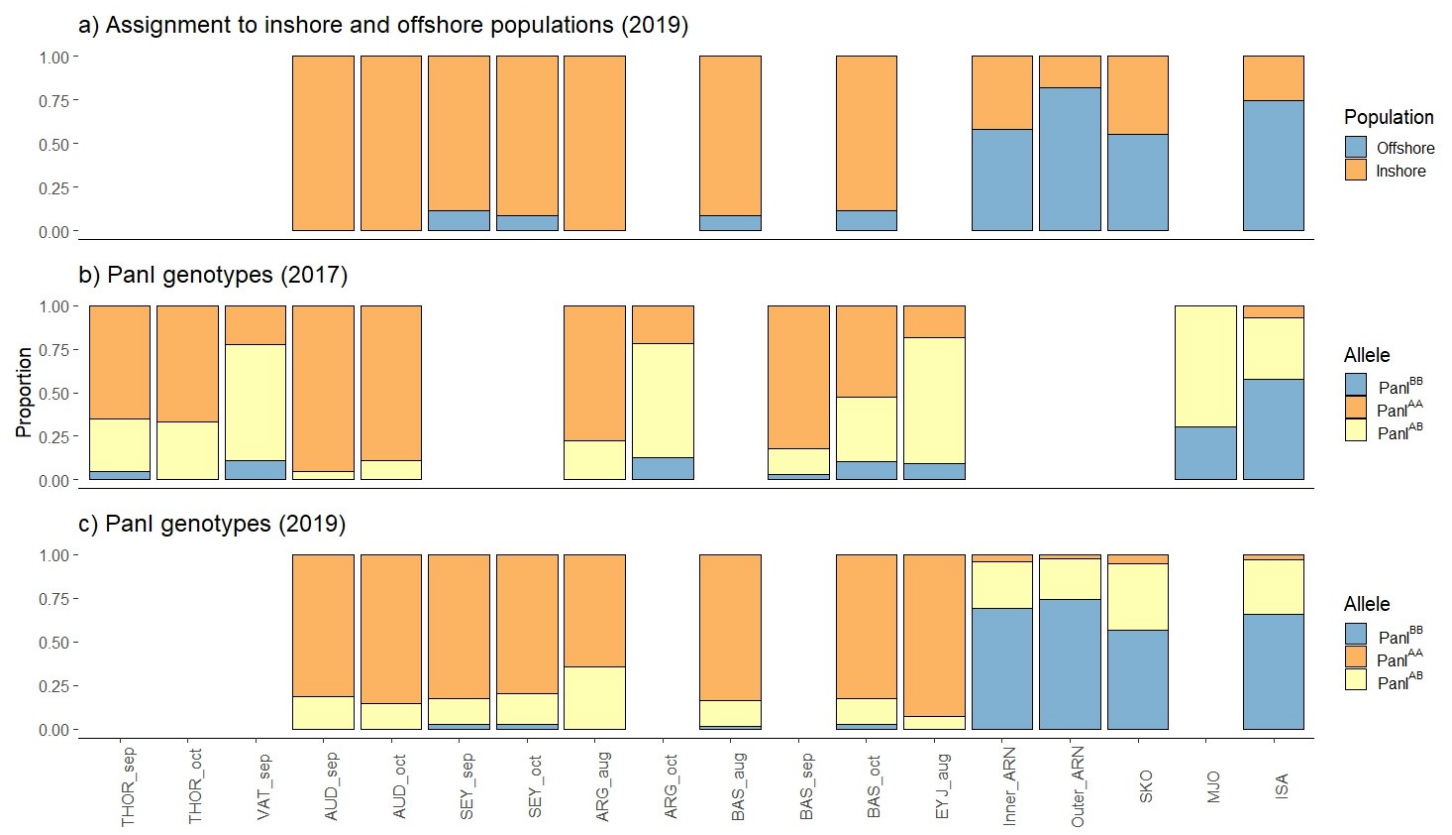
Assignment to inshore / offshore populations and Pan I genotypes. The barplots specifically depict the relative assignment of juveniles from different sample sites and times to inshore and offshore populations (a), Pan I genotypes in the 2017 sample groups (b) and Pan I genotypes in the 2019 sample groups (c). The month of sampling is indicated after the site code.

Most juveniles (> 95%) were assigned to the previously defined offshore or inshore populations with over 90% probability. Juveniles assigned to the inshore population were much more common in seined samples (Figs 3 and 4), but depth was the only significant predictor of the likelihood of inshore vs. offshore assignment (Table 3), conversely sampling gear predicted Pan I^AA^ vs. Pan I^BB^ genotypes (Table 3). It should be noted that since gear and depth were highly correlated these differing results most likely reflects the unbalances sample distribution. This is particularly relevant for the Pan I genotype dataset when sampling by gear and depth differed notably by year of sampling. Pan I^BB^ genotypes were more likely in the later months of sampling but this likely reflects that the trawling survey samples were more frequent in later months and the shrimp survey that was conducted in November in 2017 rather than in October 2019. No other geographical or temporal variations were significant.

**Table 3:** Results from binomial models examining the likelihood of inshore/offshore and Pan I genotypes in different samples. Both results show the clear differentiation by depth / gear (likely to be highly correlated). Other factors explain little of the variation and the slightly higher likelihood in later sampling may simply result from the trawling survey samples being more frequent in later months.

Predictors	Inshore vs offshore assignment				Pan I^AA^ vs Pan I^BB^ genotypes			
	Odds Ratios	SE	CI	p-value	Odds Ratios	SE	CI	p-value
(Intercept)	0.01	0.03	0.00–3.59	0.129	0.00	0.00	0.00–0.03	**0.001**
Gear [Trawl]	1.48	1.60	0.17–12.07	0.716	87.92	86.85	12.92–631.99	**<0.001**
Depth	1.05	0.02	1.02–1.08	**0.002**	1.00	0.01	0.98–1.03	0.998
Area [Ísafjarðardjúp]	1.26	0.39	0.69–2.32	0.452	1.24	0.46	0.60–2.58	0.562
Area [Strandir]	1.56	1.48	0.81–8.03	0.103	1.72	0.99	0.54–5.40	0.347
Month	1.20	0.40	0.63–2.37	0.589	1.79	0.51	1.03–3.19	**0.040**
Area [Breiðafjörður]					3.11	2.30	0.62–12.35	0.124
Observations	400				624			
R^2^ Tjur	0.432				0.702			

Examining agreement between the inshore/offshore assignment and Pan I genotypes there was a general agreement of assignment by the 96-SNP panel and the Pan I genotypes by site (Fig 4). However, there was considerable percentage of inshore individuals that were Pan I^BB^ genotypes (7.9%), but this was much less common for offshore juveniles that rarely had a Pan I^AA^ genotype (2.9%). Pan I heterozygotes were assigned as offshore in 33.9% of the cases.

## Discussion

The current study shows that juveniles of both inshore and offshore Atlantic cod populations, as defined by Therkildsen et al. [17], co-occur in nursery habitats around the Westfjords in NW Iceland. However, 0-group juveniles classed to the offshore population (and Pan I^BB^ genotypes) were rarely sampled in shallow tidal waters, and juveniles of inshore population origin (and Pan I^AA^ genotypes) make up most of the juvenile groups sampled with the beach seine and were rare at deeper stations. This pattern was consistent between years and time after benthic settlement. However, for 1-year-old cod, there was a mix of Pan I genotypes in the trawled samples with Pan I heterozygotes being the most common (Table 1).

The inshore / offshore assignment by the SNP panel and the Pan I allele frequencies showed similar differentiation by depth/gear, as is to be expected as the differentiation of Icelandic cod is most notably on LG1 [21]. Heterozygotes were common in this present study as in previous studies of the Icelandic cod stock, perhaps indicating incomplete differentiation, admixture, or a role of selection [21]. The Pan I^AA^ individuals in the current study were most often classed as inshore cod using the SNP panel. However, the classification of Pan I^BB^ and Pan I^AB^ individuals was more erratic, and almost 20% of Pan I^BB^ juveniles were assigned to the inshore population. This may highlight the relatively recent origin of Icelandic inshore populations [10] reflected in the plasticity of the Pan I^BB^ genotypes. This was previously suggested by examining the variation in Pan I genotypes with depth and temperature profiles of Atlantic cod around Iceland [21]. Although the genetic segregation by depth in the current study is clear we suggest that a comprehensive genetic analysis including pelagic and benthic juveniles as well as individuals from spawning grounds across regions is important to further understand the dynamics of demersal juvenile Atlantic cod around Iceland.

Any comparison of beach seine and trawl samples should consider different mesh sizes and gear selectivity. In the current study, the catchability of cod juveniles with the shrimp trawl depends on many factors, importantly other catches, and the retention of very small juveniles may be lower than with the beach seine. This could affect the size distribution reported by the different gear, but also genotype/population assignment frequencies, if genotypes differ by size. Juveniles and subadults of Icelandic cod Pan I genotypes have differed in growth rate [47, 48] and variation in growth rate has been found for Norwegian coastal and Northeast Arctic cod reared under identical conditions [49]. However, juvenile growth rate is highly affected by the environment [50–52], and environmentally induced variation in growth may mask any ecotype effect [48]. Consequently, disentangling genetic and environmental effects on juvenile cod size and growth can be difficult across very different life-time temperature regimes. For example, cod originating from offshore and inshore spawning grounds around Iceland did not differ in growth rate when measured at standardized aquaculture conditions [53]. In the current study, 0-group juvenile cod caught by beach seining were much smaller than juveniles caught in trawls, although size is more associated with geographical and temporal variation (Table 2, Fig 2). Nevertheless, the current results could partly reflect coastal juveniles being equally likely to settle at a range of depths but less likely to be caught in the shrimp trawl because of their smaller size. However, it is unlikely that fishing gear selectivity explains the lack of offshore/Pan I^BB^ genotypes in tidal waters.

Segregation by depth of migratory and coastal ecotypes was described in northern Norway a decade ago [33]. The authors concluded that environmental preferences, perhaps based on the divergent adaptations of cod ecotypes to different salinity levels during glacial epochs, could explain the depth segregation [33]. Moreover, a recent analysis of juvenile Atlantic cod aggregations along the west coast of Sweden shows that the North Sea (offshore) and coastal juveniles segregate by depth and suggests that the divergence is associated with environmental adaptations, such as, to temperature, hypoxia, and salinity [34]. The current study cannot be used to identify specific drivers of depth divergence, and many environmental factors co-vary with depth, notably temperature and light. Icelandic fjords are not very stratified and salinity at the beach seining sites in the current study was always above 32.5 PSU. In Arnarfjörður, the only fjord were vertical salinity profiles were available, salinity varies from c.a. 32.5 PSU at the surface to 35 or 36 PSU at benthic depths [54]. Although the difference in salinity is not as pronounced as reported in Norway, the variation of 2–3 PSU in Iceland could result in habitat selection. However, temperature [55] and light regime [56] have also been associated with divergence on LG1, the most divergent genomic region between Icelandic cod migratory types [21]. Rhodopsin, a gene that encodes variant visual pigments differs by depth profiles in Icelandic cod [56] and was also an outlier in the current analysis. Still, several other stressors or impacts vary by depth. Young adult cod infested by ectoparasites have, for example, been shown to inhabit deeper waters [57], and parasite load has been suggested as a biomarker for cod ecotypes as they experience very different infestation levels in their lifetime [58]. Juveniles from inshore populations may merely be more physiologically adept to tolerate the stressors associated with the tidal habitat, for example, fluctuations in environmental factors. The benefits of residing in the shallow habitat are likely to include low predation as well as high and diverse food availability [59]. A recent study from Norway found high growth of coastal cod 0-group juveniles in very shallow water [60]. Finally, a recent study indicates a relationship between individual tendency for exploration and Pan I genotypes in 0-group juvenile cod [61] supporting that innate behavioral differences could also influence habitat choice.

It should also be considered that juvenile aggregations can differ in genetic composition because of the temporal variation in the arrival and settlement of juveniles from geographically, or temporally, different spawning components. The prevalence of juvenile cod aggregations from the North Sea vs. local groups in Skagerrak, for example, differ temporally [30, 31, 37]. Atlantic cod at the large spawning grounds off SW Iceland spawn earlier and may grow initially faster, than smaller spawning components along the west coast [23, 25]. A possible explanation for the current pattern would be that the offshore juveniles inhabited the shallow tidal habitat earlier than juveniles from inshore populations that spawn later in the season. However, no evidence was found for larger aggregations of offshore juvenile cod in tidal waters earlier in the season, within the temporal range of the current sampling, and there were no differences in the genetic composition of November 2017 and October 2019 Ísafjörður (ISA) trawling sites. Therefore, we infer that the temporal variation in benthic settlement of juveniles from different spawning grounds is unlikely to fully explain the current results but recommend that a more comprehensive study, including repeated within-year sampling at both shallow and deeper sites and including otolith analysis for inference of hatch date, growth and age.

To conclude, we show that genotypes of 0-group Atlantic cod juveniles in NW- Iceland differ by depth at the time of benthic settlement, and in the following weeks. Possible environmental drivers of the divergence cannot be determined but such selection mechanisms could be experimentally examined. The current results add significantly to the two previous studies of similar segregation and highlight that physiological divergence is already clear in 0-group juvenile cod. With the clear climate-driven northward shift of Atlantic cod and the establishment of local northern populations, understanding how genotypes and environmental factors shape distribution is a critical issue. From a national management perspective, the results highlight the need for juvenile surveys to include a range of depths and times and suggest that the anthropogenic impacts in coastal waters disproportionately affect inshore juveniles. Conservation efforts should focus on protecting the total within species biodiversity, and population segregation at nursery grounds should be considered in the application of future marine spatial planning and other area-based conservation tools.

## Supporting information

S1 Table Dataset. (DOCX)

S2 Table Pairwise F_ST_ values between sample groups. (PDF)

S1 Fig Juvenile standard length distributions. (PDF)

S2 Fig 1-year-old juvenile standard length. (PDF)
