# Supplementary material for: Genetic assignment predicts depth of benthic settlement for 0-group Atlantic cod
Source: PLoS One. 2023 Oct 4;18(10):e0292495. doi: 10.1371/journal.pone.0292495 (PMC10550133; doi:10.1371/journal.pone.0292495)
Supplement: S1 Table — (DOCX) [file pone.0292495.s001.docx]

**S1 Table. Dataset.** Differences in the depth of benthic settlement of 0-group Atlantic cod of offshore and inshore origin. Corresponding author: [gaol@hi.is](mailto:gaol@hi.is)

| ID | Gear | Area | Length | Age | Weight | Depth | Site | Site.code | Latitude | Longitude | Day | Month | Year | SNP.assignment | Pan.genotype |
| --- | --- | --- | --- | --- | --- | --- | --- | --- | --- | --- | --- | --- | --- | --- | --- |
|  |  |  |  |  |  |  |  |  |  |  |  |  |  |  |  |
| A1244T1 | Trawl | Isafjordur | 9.1 | 0 | 9.73 | 63 | Skotufjordur | SKO-T | 65.5868 | -22.2582 | 8 | 10 | 2019 | Inshore | AB |
| A1244T10 | Trawl | Isafjordur | 6.8 | 0 | 3.5 | 63 | Skotufjordur | SKO-T | 65.5868 | -22.2582 | 8 | 10 | 2019 | Inshore | AB |
| A1244T13 | Trawl | Isafjordur | 7.4 | 0 | 4.91 | 63 | Skotufjordur | SKO-T | 65.5868 | -22.2582 | 8 | 10 | 2019 | Inshore | AA |
| A1244T14 | Trawl | Isafjordur | 8 | 0 | 6.36 | 63 | Skotufjordur | SKO-T | 65.5868 | -22.2582 | 8 | 10 | 2019 | Inshore | AB |
| AD109BS2 | Seine | Arnarfjordur | 16.5 | 1 | NA | 0 | Audkula | AUD-S | 65.96488 | -22.89689 | 10 | 9 | 2019 | Inshore | NA |
| A1244T16 | Trawl | Isafjordur | 7.6 | 0 | 4.6 | 63 | Skotufjordur | SKO-T | 65.5868 | -22.2582 | 8 | 10 | 2019 | Inshore | BB |
| A1244T17 | Trawl | Isafjordur | 7 | 0 | 3.61 | 63 | Skotufjordur | SKO-T | 65.5868 | -22.2582 | 8 | 10 | 2019 | Inshore | BB |
| A1244T18 | Trawl | Isafjordur | NA | 0 | NA | 63 | Skotufjordur | SKO-T | 65.5868 | -22.2582 | 8 | 10 | 2019 | Inshore | NA |
| A1244T2 | Trawl | Isafjordur | 7.1 | 0 | 4.36 | 63 | Skotufjordur | SKO-T | 65.5868 | -22.2582 | 8 | 10 | 2019 | Inshore | BB |
| A1249T10 | Trawl | Isafjordur | 6.9 | 0 | 3.62 | 48 | Skotufjordur | SKO-T | 65.5509 | -22.2432 | 8 | 10 | 2019 | Inshore | BB |
| BS268BS7 | Seine | Strandir | 2.5 | 0 | 0.14 | 0 | Bassastadir | BAS-S | 65.8945 | -22.387 | 26 | 8 | 2019 | Inshore | AA |
| A1249T12 | Trawl | Isafjordur | 6.9 | 0 | 3.27 | 48 | Skotufjordur | SKO-T | 65.5509 | -22.2432 | 8 | 10 | 2019 | Inshore | AB |
| A1249T15 | Trawl | Isafjordur | 7.2 | 0 | 4.08 | 48 | Skotufjordur | SKO-T | 65.5509 | -22.2432 | 8 | 10 | 2019 | Inshore | AB |
| A1249T16 | Trawl | Isafjordur | 7.3 | 0 | 4.14 | 48 | Skotufjordur | SKO-T | 65.5509 | -22.2432 | 8 | 10 | 2019 | Inshore | AA |
| A1249T17 | Trawl | Isafjordur | 7.4 | 0 | 4.2 | 48 | Skotufjordur | SKO-T | 65.5509 | -22.2432 | 8 | 10 | 2019 | Inshore | BB |
| A1249T18 | Trawl | Isafjordur | NA | 0 | NA | 48 | Skotufjordur | SKO-T | 65.5509 | -22.2432 | 8 | 10 | 2019 | Inshore | NA |
| BS268BS14 | Seine | Strandir | 3.2 | 0 | 0.31 | 0 | Bassastadir | BAS-S | 65.8945 | -22.387 | 26 | 8 | 2019 | Inshore | AA |
| BS268BS44 | Seine | Strandir | 3.2 | 0 | 0.21 | 0 | Bassastadir | BAS-S | 65.8945 | -22.387 | 26 | 8 | 2019 | Inshore | AA |
| BS268BS41 | Seine | Strandir | 3.3 | 0 | 0.36 | 0 | Bassastadir | BAS-S | 65.8945 | -22.387 | 26 | 8 | 2019 | Inshore | AA |
| BS268BS13 | Seine | Strandir | 3.5 | 0 | 0.37 | 0 | Bassastadir | BAS-S | 65.8945 | -22.387 | 26 | 8 | 2019 | Inshore | AA |
| BS268BS36 | Seine | Strandir | 3.5 | 0 | 0.4 | 0 | Bassastadir | BAS-S | 65.8945 | -22.387 | 26 | 8 | 2019 | Inshore | AA |
| BS268BS42 | Seine | Strandir | 3.5 | 0 | 0.46 | 0 | Bassastadir | BAS-S | 65.8945 | -22.387 | 26 | 8 | 2019 | Inshore | AA |
| BS268BS55 | Seine | Strandir | 3.6 | 0 | 0.42 | 0 | Bassastadir | BAS-S | 65.8945 | -22.387 | 26 | 8 | 2019 | Inshore | AA |
| BS268BS6 | Seine | Strandir | 3.7 | 0 | 0.55 | 0 | Bassastadir | BAS-S | 65.8945 | -22.387 | 26 | 8 | 2019 | Inshore | AA |
| BS268BS39 | Seine | Strandir | 3.9 | 0 | 0.52 | 0 | Bassastadir | BAS-S | 65.8945 | -22.387 | 26 | 8 | 2019 | Inshore | AB |
| BS268BS5 | Seine | Strandir | 3.9 | 0 | 0.6 | 0 | Bassastadir | BAS-S | 65.8945 | -22.387 | 26 | 8 | 2019 | Inshore | AA |
| BS268BS8 | Seine | Strandir | 3.9 | 0 | 0.49 | 0 | Bassastadir | BAS-S | 65.8945 | -22.387 | 26 | 8 | 2019 | Inshore | AA |
| A1249T19 | Trawl | Isafjordur | 7.7 | 0 | 4.53 | 48 | Skotufjordur | SKO-T | 65.5509 | -22.2432 | 8 | 10 | 2019 | Inshore | BB |
| AA109BS14 | Seine | Arnarfjordur | 4 | 0 | 0.52 | 0 | Audkula | AUD-S | 65.96486 | -22.89687 | 10 | 9 | 2019 | Inshore | AA |
| AA109BS37 | Seine | Arnarfjordur | 4.3 | 0 | 0.89 | 0 | Audkula | AUD-S | 65.96486 | -22.89687 | 10 | 9 | 2019 | Inshore | AA |
| BS268BS27 | Seine | Strandir | 4.3 | 0 | 0.77 | 0 | Bassastadir | BAS-S | 65.8945 | -22.387 | 26 | 8 | 2019 | Inshore | AB |
| A1249T4 | Trawl | Isafjordur | 6.3 | 0 | 2.44 | 48 | Skotufjordur | SKO-T | 65.5509 | -22.2432 | 8 | 10 | 2019 | Inshore | BB |
| AA109BS23 | Seine | Arnarfjordur | 4.4 | 0 | 0.85 | 0 | Audkula | AUD-S | 65.96486 | -22.89687 | 10 | 9 | 2019 | Inshore | AA |
| AA109BS34 | Seine | Arnarfjordur | 4.4 | 0 | 0.92 | 0 | Audkula | AUD-S | 65.96486 | -22.89687 | 10 | 9 | 2019 | Inshore | AA |
| BS268BS23 | Seine | Strandir | 4.4 | 0 | 0.75 | 0 | Bassastadir | BAS-S | 65.8945 | -22.387 | 26 | 8 | 2019 | Inshore | AA |
| BS268BS24 | Seine | Strandir | 4.4 | 0 | 0.82 | 0 | Bassastadir | BAS-S | 65.8945 | -22.387 | 26 | 8 | 2019 | Inshore | AA |
| BS268BS40 | Seine | Strandir | 4.4 | 0 | 0.84 | 0 | Bassastadir | BAS-S | 65.8945 | -22.387 | 26 | 8 | 2019 | Inshore | AA |
| AA109BS11 | Seine | Arnarfjordur | 4.5 | 0 | 0.94 | 0 | Audkula | AUD-S | 65.96486 | -22.89687 | 10 | 9 | 2019 | Inshore | AA |
| AA109BS18 | Seine | Arnarfjordur | 4.5 | 0 | 0.97 | 0 | Audkula | AUD-S | 65.96486 | -22.89687 | 10 | 9 | 2019 | Inshore | AA |
| AA109BS19 | Seine | Arnarfjordur | 4.5 | 0 | 0.81 | 0 | Audkula | AUD-S | 65.96486 | -22.89687 | 10 | 9 | 2019 | Inshore | AB |
| AA109BS20 | Seine | Arnarfjordur | 4.5 | 0 | 0.93 | 0 | Audkula | AUD-S | 65.96486 | -22.89687 | 10 | 9 | 2019 | Inshore | AA |
| AA109BS22 | Seine | Arnarfjordur | 4.5 | 0 | 0.87 | 0 | Audkula | AUD-S | 65.96486 | -22.89687 | 10 | 9 | 2019 | Inshore | AB |
| AA109BS25 | Seine | Arnarfjordur | 4.5 | 0 | 0.89 | 0 | Audkula | AUD-S | 65.96486 | -22.89687 | 10 | 9 | 2019 | Inshore | AA |
| AA109BS29 | Seine | Arnarfjordur | 4.5 | 0 | 0.89 | 0 | Audkula | AUD-S | 65.96486 | -22.89687 | 10 | 9 | 2019 | Inshore | AA |
| BS268BS32 | Seine | Strandir | 4.5 | 0 | 1.07 | 0 | Bassastadir | BAS-S | 65.8945 | -22.387 | 26 | 8 | 2019 | Inshore | AA |
| A933T6 | Trawl | Arnarfjordur | 4.52 | 0 | 7.3 | 64 | Outer_Arnarfjordur | Outer_ARN-T | 65.8031 | -23.7978 | 1 | 10 | 2019 | Inshore | AB |
| AA109BS38 | Seine | Arnarfjordur | 4.6 | 0 | 1.06 | 0 | Audkula | AUD-S | 65.96486 | -22.89687 | 10 | 9 | 2019 | Inshore | AA |
| BS268BS11 | Seine | Strandir | 4.6 | 0 | 0.85 | 0 | Bassastadir | BAS-S | 65.8945 | -22.387 | 26 | 8 | 2019 | Inshore | AA |
| BS268BS15 | Seine | Strandir | 4.6 | 0 | 0.69 | 0 | Bassastadir | BAS-S | 65.8945 | -22.387 | 26 | 8 | 2019 | Inshore | AA |
| BS268BS2 | Seine | Strandir | 4.6 | 0 | 0.8 | 0 | Bassastadir | BAS-S | 65.8945 | -22.387 | 26 | 8 | 2019 | Inshore | AA |
| AA109BS15 | Seine | Arnarfjordur | 4.7 | 0 | 1.11 | 0 | Audkula | AUD-S | 65.96486 | -22.89687 | 10 | 9 | 2019 | Inshore | AA |
| AA109BS16 | Seine | Arnarfjordur | 4.7 | 0 | 1.14 | 0 | Audkula | AUD-S | 65.96486 | -22.89687 | 10 | 9 | 2019 | Inshore | AA |
| AA109BS32 | Seine | Arnarfjordur | 4.7 | 0 | 0.94 | 0 | Audkula | AUD-S | 65.96486 | -22.89687 | 10 | 9 | 2019 | Inshore | AA |
| BS268BS21 | Seine | Strandir | 4.7 | 0 | 1.16 | 0 | Bassastadir | BAS-S | 65.8945 | -22.387 | 26 | 8 | 2019 | Inshore | AA |
| BS268BS22 | Seine | Strandir | 4.7 | 0 | 0.93 | 0 | Bassastadir | BAS-S | 65.8945 | -22.387 | 26 | 8 | 2019 | Inshore | AA |
| A933T2 | Trawl | Arnarfjordur | 4.8 | 0 | 7.5 | 64 | Outer_Arnarfjordur | Outer_ARN-T | 65.8031 | -23.7978 | 1 | 10 | 2019 | Inshore | BB |
| AA109BS1 | Seine | Arnarfjordur | 4.8 | 0 | 1.34 | 0 | Audkula | AUD-S | 65.96486 | -22.89687 | 10 | 9 | 2019 | Inshore | AA |
| AA109BS26 | Seine | Arnarfjordur | 4.8 | 0 | 1.09 | 0 | Audkula | AUD-S | 65.96486 | -22.89687 | 10 | 9 | 2019 | Inshore | AA |
| AA109BS28 | Seine | Arnarfjordur | 4.8 | 0 | 1.19 | 0 | Audkula | AUD-S | 65.96486 | -22.89687 | 10 | 9 | 2019 | Inshore | AA |
| AA109BS36 | Seine | Arnarfjordur | 4.8 | 0 | 1.12 | 0 | Audkula | AUD-S | 65.96486 | -22.89687 | 10 | 9 | 2019 | Inshore | AA |
| AA109BS5 | Seine | Arnarfjordur | 4.8 | 0 | 0.94 | 0 | Audkula | AUD-S | 65.96486 | -22.89687 | 10 | 9 | 2019 | Inshore | AA |
| AA109BS7 | Seine | Arnarfjordur | 4.8 | 0 | 1.26 | 0 | Audkula | AUD-S | 65.96486 | -22.89687 | 10 | 9 | 2019 | Inshore | AB |
| BS268BS9 | Seine | Strandir | 4.8 | 0 | 0.81 | 0 | Bassastadir | BAS-S | 65.8945 | -22.387 | 26 | 8 | 2019 | Inshore | AA |
| AA109BS27 | Seine | Arnarfjordur | 4.9 | 0 | 1.09 | 0 | Audkula | AUD-S | 65.96486 | -22.89687 | 10 | 9 | 2019 | Inshore | AA |
| BS268BS62 | Seine | Strandir | 4.9 | 0 | 1.14 | 0 | Bassastadir | BAS-S | 65.8945 | -22.387 | 26 | 8 | 2019 | Inshore | AA |
| A1249T6 | Trawl | Isafjordur | 6.6 | 0 | 2.63 | 48 | Skotufjordur | SKO-T | 65.5509 | -22.2432 | 8 | 10 | 2019 | Inshore | BB |
| AA109BS12 | Seine | Arnarfjordur | 5 | 0 | 1.35 | 0 | Audkula | AUD-S | 65.96486 | -22.89687 | 10 | 9 | 2019 | Inshore | AA |
| AA109BS13 | Seine | Arnarfjordur | 5 | 0 | 1.42 | 0 | Audkula | AUD-S | 65.96486 | -22.89687 | 10 | 9 | 2019 | Inshore | AA |
| AA109BS2 | Seine | Arnarfjordur | 5 | 0 | 1.28 | 0 | Audkula | AUD-S | 65.96486 | -22.89687 | 10 | 9 | 2019 | Inshore | AA |
| AA109BS24 | Seine | Arnarfjordur | 5 | 0 | 1.01 | 0 | Audkula | AUD-S | 65.96486 | -22.89687 | 10 | 9 | 2019 | Inshore | AA |
| AA109BS35 | Seine | Arnarfjordur | 5 | 0 | 1.31 | 0 | Audkula | AUD-S | 65.96486 | -22.89687 | 10 | 9 | 2019 | Inshore | AA |
| BS268BS10 | Seine | Strandir | 5 | 0 | 1.12 | 0 | Bassastadir | BAS-S | 65.8945 | -22.387 | 26 | 8 | 2019 | Inshore | AA |
| A1249T8 | Trawl | Isafjordur | 6.7 | 0 | 3.49 | 48 | Skotufjordur | SKO-T | 65.5509 | -22.2432 | 8 | 10 | 2019 | Inshore | AB |
| BS268BS3 | Seine | Strandir | 5.1 | 0 | 1.68 | 0 | Bassastadir | BAS-S | 65.8945 | -22.387 | 26 | 8 | 2019 | Inshore | AA |
| BS268BS43 | Seine | Strandir | 5.1 | 0 | 1.24 | 0 | Bassastadir | BAS-S | 65.8945 | -22.387 | 26 | 8 | 2019 | Inshore | AA |
| A1282T10 | Trawl | Isafjordur | NA | 0 | NA | 68 | Isafjordur | ISA-T | 65.9833 | -22.4923 | 9 | 10 | 2019 | Inshore | NA |
| A1282T11 | Trawl | Isafjordur | NA | 0 | NA | 68 | Isafjordur | ISA-T | 65.9833 | -22.4923 | 9 | 10 | 2019 | Inshore | NA |
| AA109BS30 | Seine | Arnarfjordur | 5.2 | 0 | 1.47 | 0 | Audkula | AUD-S | 65.96486 | -22.89687 | 10 | 9 | 2019 | Inshore | AA |
| AA109BS8 | Seine | Arnarfjordur | 5.2 | 0 | 1.55 | 0 | Audkula | AUD-S | 65.96486 | -22.89687 | 10 | 9 | 2019 | Inshore | AB |
| AA109BS17 | Seine | Arnarfjordur | 5.3 | 0 | 1.62 | 0 | Audkula | AUD-S | 65.96486 | -22.89687 | 10 | 9 | 2019 | Inshore | AA |
| AA109BS3 | Seine | Arnarfjordur | 5.3 | 0 | 1.72 | 0 | Audkula | AUD-S | 65.96486 | -22.89687 | 10 | 9 | 2019 | Inshore | AA |
| AA109BS31 | Seine | Arnarfjordur | 5.3 | 0 | 1.71 | 0 | Audkula | AUD-S | 65.96486 | -22.89687 | 10 | 9 | 2019 | Inshore | AB |
| A1282T13 | Trawl | Isafjordur | NA | 0 | NA | 68 | Isafjordur | ISA-T | 65.9833 | -22.4923 | 9 | 10 | 2019 | Inshore | NA |
| A1282T14 | Trawl | Isafjordur | NA | 0 | NA | 68 | Isafjordur | ISA-T | 65.9833 | -22.4923 | 9 | 10 | 2019 | Inshore | NA |
| AA109BS10 | Seine | Arnarfjordur | 5.4 | 0 | 1.7 | 0 | Audkula | AUD-S | 65.96486 | -22.89687 | 10 | 9 | 2019 | Inshore | AA |
| BS268BS18 | Seine | Strandir | 5.4 | 0 | 1.38 | 0 | Bassastadir | BAS-S | 65.8945 | -22.387 | 26 | 8 | 2019 | Inshore | AA |
| AA109BS21 | Seine | Arnarfjordur | 5.5 | 0 | 1.65 | 0 | Audkula | AUD-S | 65.96486 | -22.89687 | 10 | 9 | 2019 | Inshore | AA |
| AA109BS9 | Seine | Arnarfjordur | 5.5 | 0 | 1.62 | 0 | Audkula | AUD-S | 65.96486 | -22.89687 | 10 | 9 | 2019 | Inshore | AB |
| BS268BS4 | Seine | Strandir | 5.5 | 0 | 1.76 | 0 | Bassastadir | BAS-S | 65.8945 | -22.387 | 26 | 8 | 2019 | Inshore | AA |
| AA109BS6 | Seine | Arnarfjordur | 5.6 | 0 | 1.8 | 0 | Audkula | AUD-S | 65.96486 | -22.89687 | 10 | 9 | 2019 | Inshore | AA |
| AD109BS1 | Seine | Arnarfjordur | 5.6 | 0 | NA | 1 | Audkula | AUD-S | 65.96487 | -22.89688 | 10 | 9 | 2019 | Inshore | NA |
| A1282T18 | Trawl | Isafjordur | NA | 0 | NA | 68 | Isafjordur | ISA-T | 65.9833 | -22.4923 | 9 | 10 | 2019 | Inshore | NA |
| A1282T3 | Trawl | Isafjordur | NA | 0 | NA | 68 | Isafjordur | ISA-T | 65.9833 | -22.4923 | 9 | 10 | 2019 | Inshore | NA |
| A933T13 | Trawl | Arnarfjordur | 5.72 | 0 | 8 | 64 | Outer_Arnarfjordur | Outer_ARN-T | 65.8031 | -23.7978 | 1 | 10 | 2019 | Inshore | AB |
| A1072T12 | Trawl | Arnarfjordur | 5.8 | 0 | 1.83 | 63 | Inner_Arnarfjordur | Inner_ARN-T | 65.4476 | -23.185 | 4 | 10 | 2019 | Inshore | BB |
| AA109BS4 | Seine | Arnarfjordur | 5.8 | 0 | 1.99 | 0 | Audkula | AUD-S | 65.96486 | -22.89687 | 10 | 9 | 2019 | Inshore | AA |
| A1282T7 | Trawl | Isafjordur | NA | 0 | NA | 68 | Isafjordur | ISA-T | 65.9833 | -22.4923 | 9 | 10 | 2019 | Inshore | NA |
| A1076T7 | Trawl | Arnarfjordur | 5.9 | 0 | 1.88 | 48 | Inner_Arnarfjordur | Inner_ARN-T | 65.4421 | -23.2196 | 4 | 10 | 2019 | Inshore | AB |
| E101 | Seine | Isafjordur | NA | 0 | NA | 0 | Seydisfjordur | SEY-S | 65.95817 | -22.38883 | 9 | 10 | 2019 | Inshore | AB |
| E102 | Seine | Isafjordur | NA | 0 | NA | 0 | Seydisfjordur | SEY-S | 65.95817 | -22.38883 | 9 | 10 | 2019 | Inshore | AA |
| E11 | Seine | Isafjordur | NA | 0 | NA | 0 | Seydisfjordur | SEY-S | 65.95817 | -22.38883 | 9 | 10 | 2019 | Inshore | AA |
| E12 | Seine | Isafjordur | NA | 0 | NA | 0 | Seydisfjordur | SEY-S | 65.95817 | -22.38883 | 9 | 10 | 2019 | Inshore | AB |
| E13 | Seine | Isafjordur | NA | 0 | NA | 0 | Seydisfjordur | SEY-S | 65.95817 | -22.38883 | 9 | 10 | 2019 | Inshore | AA |
| E14 | Seine | Isafjordur | NA | 0 | NA | 0 | Seydisfjordur | SEY-S | 65.95817 | -22.38883 | 9 | 10 | 2019 | Inshore | AA |
| E15 | Seine | Isafjordur | NA | 0 | NA | 0 | Seydisfjordur | SEY-S | 65.95817 | -22.38883 | 9 | 10 | 2019 | Inshore | AA |
| A1076T9 | Trawl | Arnarfjordur | 6.3 | 0 | 2.65 | 48 | Inner_Arnarfjordur | Inner_ARN-T | 65.4421 | -23.2196 | 4 | 10 | 2019 | Inshore | AB |
| E16 | Seine | Isafjordur | NA | 0 | NA | 0 | Seydisfjordur | SEY-S | 65.95817 | -22.38883 | 9 | 10 | 2019 | Inshore | AA |
| E19 | Seine | Isafjordur | NA | 0 | NA | 0 | Seydisfjordur | SEY-S | 65.95817 | -22.38883 | 9 | 10 | 2019 | Inshore | AA |
| A1076T6 | Trawl | Arnarfjordur | 6.4 | 0 | 2.49 | 48 | Inner_Arnarfjordur | Inner_ARN-T | 65.4421 | -23.2196 | 4 | 10 | 2019 | Inshore | AB |
| E23 | Seine | Isafjordur | NA | 0 | NA | 0 | Seydisfjordur | SEY-S | 65.95817 | -22.38883 | 9 | 10 | 2019 | Inshore | AA |
| E24 | Seine | Isafjordur | NA | 0 | NA | 0 | Seydisfjordur | SEY-S | 65.95817 | -22.38883 | 9 | 10 | 2019 | Inshore | AA |
| E25 | Seine | Isafjordur | NA | 0 | NA | 0 | Seydisfjordur | SEY-S | 65.95817 | -22.38883 | 9 | 10 | 2019 | Inshore | AA |
| A1017T13 | Trawl | Arnarfjordur | 6.6 | 0 | 3.43 | 85 | Inner_Arnarfjordur | Inner_ARN-T | 65.4416 | -23.3048 | 3 | 10 | 2019 | Inshore | AA |
| A1072T8 | Trawl | Arnarfjordur | 6.6 | 0 | 3.18 | 63 | Inner_Arnarfjordur | Inner_ARN-T | 65.4476 | -23.185 | 4 | 10 | 2019 | Inshore | BB |
| E26 | Seine | Isafjordur | NA | 0 | NA | 0 | Seydisfjordur | SEY-S | 65.95817 | -22.38883 | 9 | 10 | 2019 | Inshore | AA |
| E28 | Seine | Isafjordur | NA | 0 | NA | 0 | Seydisfjordur | SEY-S | 65.95817 | -22.38883 | 9 | 10 | 2019 | Inshore | AA |
| E29 | Seine | Isafjordur | NA | 0 | NA | 0 | Seydisfjordur | SEY-S | 65.95817 | -22.38883 | 9 | 10 | 2019 | Inshore | AB |
| E42 | Seine | Isafjordur | NA | 0 | NA | 0 | Seydisfjordur | SEY-S | 65.95817 | -22.38883 | 9 | 10 | 2019 | Inshore | AA |
| E51 | Seine | Isafjordur | NA | 0 | NA | 0 | Seydisfjordur | SEY-S | 65.95817 | -22.38883 | 9 | 10 | 2019 | Inshore | AA |
| A1076T10 | Trawl | Arnarfjordur | 6.7 | 0 | 3.14 | 48 | Inner_Arnarfjordur | Inner_ARN-T | 65.4421 | -23.2196 | 4 | 10 | 2019 | Inshore | AB |
| E52 | Seine | Isafjordur | NA | 0 | NA | 0 | Seydisfjordur | SEY-S | 65.95817 | -22.38883 | 9 | 10 | 2019 | Inshore | BB |
| E58 | Seine | Isafjordur | NA | 0 | NA | 0 | Seydisfjordur | SEY-S | 65.95817 | -22.38883 | 9 | 10 | 2019 | Inshore | AA |
| E61 | Seine | Isafjordur | NA | 0 | NA | 0 | Seydisfjordur | SEY-S | 65.95817 | -22.38883 | 9 | 10 | 2019 | Inshore | AA |
| A1072T11 | Trawl | Arnarfjordur | 6.8 | 0 | 3.47 | 63 | Inner_Arnarfjordur | Inner_ARN-T | 65.4476 | -23.185 | 4 | 10 | 2019 | Inshore | BB |
| A1076T8 | Trawl | Arnarfjordur | 6.8 | 0 | 3.23 | 48 | Inner_Arnarfjordur | Inner_ARN-T | 65.4421 | -23.2196 | 4 | 10 | 2019 | Inshore | BB |
| E62 | Seine | Isafjordur | NA | 0 | NA | 0 | Seydisfjordur | SEY-S | 65.95817 | -22.38883 | 9 | 10 | 2019 | Inshore | AA |
| E63 | Seine | Isafjordur | NA | 0 | NA | 0 | Seydisfjordur | SEY-S | 65.95817 | -22.38883 | 9 | 10 | 2019 | Inshore | AA |
| E64 | Seine | Isafjordur | NA | 0 | NA | 0 | Seydisfjordur | SEY-S | 65.95817 | -22.38883 | 9 | 10 | 2019 | Inshore | AA |
| E65 | Seine | Isafjordur | NA | 0 | NA | 0 | Seydisfjordur | SEY-S | 65.95817 | -22.38883 | 9 | 10 | 2019 | Inshore | AA |
| E66 | Seine | Isafjordur | NA | 0 | NA | 0 | Seydisfjordur | SEY-S | 65.95817 | -22.38883 | 9 | 10 | 2019 | Inshore | AA |
| E7 | Seine | Isafjordur | NA | 0 | NA | 0 | Seydisfjordur | SEY-S | 65.95817 | -22.38883 | 9 | 10 | 2019 | Inshore | AB |
| E75 | Seine | Isafjordur | NA | 0 | NA | 0 | Seydisfjordur | SEY-S | 65.95817 | -22.38883 | 9 | 10 | 2019 | Inshore | BB |
| E8 | Seine | Isafjordur | NA | 0 | NA | 0 | Seydisfjordur | SEY-S | 65.95817 | -22.38883 | 9 | 10 | 2019 | Inshore | AA |
| E81 | Seine | Isafjordur | NA | 0 | NA | 0 | Seydisfjordur | SEY-S | 65.95817 | -22.38883 | 9 | 10 | 2019 | Inshore | AA |
| E82 | Seine | Isafjordur | NA | 0 | NA | 0 | Seydisfjordur | SEY-S | 65.95817 | -22.38883 | 9 | 10 | 2019 | Inshore | AA |
| E9 | Seine | Isafjordur | NA | 0 | NA | 0 | Seydisfjordur | SEY-S | 65.95817 | -22.38883 | 9 | 10 | 2019 | Inshore | AA |
| A1072T15 | Trawl | Arnarfjordur | 7.1 | 0 | 3.56 | 63 | Inner_Arnarfjordur | Inner_ARN-T | 65.4476 | -23.185 | 4 | 10 | 2019 | Inshore | BB |
| A1072T4 | Trawl | Arnarfjordur | 7.1 | 0 | 3.68 | 63 | Inner_Arnarfjordur | Inner_ARN-T | 65.4476 | -23.185 | 4 | 10 | 2019 | Inshore | BB |
| A933T20 | Trawl | Arnarfjordur | 7.17 | 0 | 8.7 | 64 | Outer_Arnarfjordur | Outer_ARN-T | 65.8031 | -23.7978 | 1 | 10 | 2019 | Inshore | AB |
| A1017T12 | Trawl | Arnarfjordur | 7.3 | 0 | 5.11 | 85 | Inner_Arnarfjordur | Inner_ARN-T | 65.4416 | -23.3048 | 3 | 10 | 2019 | Inshore | AB |
| A1076T11 | Trawl | Arnarfjordur | 7.9 | 0 | 5.15 | 48 | Inner_Arnarfjordur | Inner_ARN-T | 65.4421 | -23.2196 | 4 | 10 | 2019 | Inshore | AB |
| A980T6 | Trawl | Arnarfjordur | 8.1 | 0 | 5.94 | 58 | Outer_Arnarfjordur | Outer_ARN-T | 65.4243 | -23.322 | 2 | 10 | 2019 | Inshore | AB |
| I1322T13 | Trawl | Isafjordur | 7.3 | 0 | 3.92 | 75 | Isafjordur | ISA-T | 65.572 | -22.4802 | 10 | 10 | 2019 | Inshore | AB |
| A1017T1 | Trawl | Arnarfjordur | 8.5 | 0 | 6.46 | 85 | Inner_Arnarfjordur | Inner_ARN-T | 65.4416 | -23.3048 | 3 | 10 | 2019 | Inshore | BB |
| I1322T16 | Trawl | Isafjordur | 6.7 | 0 | 2.82 | 75 | Isafjordur | ISA-T | 65.572 | -22.4802 | 10 | 10 | 2019 | Inshore | AB |
| A1072T5 | Trawl | Arnarfjordur | 8.7 | 0 | 6.85 | 63 | Inner_Arnarfjordur | Inner_ARN-T | 65.4476 | -23.185 | 4 | 10 | 2019 | Inshore | AB |
| I1322T22 | Trawl | Isafjordur | 8 | 0 | 5.4 | 75 | Isafjordur | ISA-T | 65.572 | -22.4802 | 10 | 10 | 2019 | Inshore | AB |
| A980T14 | Trawl | Arnarfjordur | 9 | 0 | 7.45 | 58 | Outer_Arnarfjordur | Outer_ARN-T | 65.4243 | -23.322 | 2 | 10 | 2019 | Inshore | AA |
| I1322T23 | Trawl | Isafjordur | 7 | 0 | 3.79 | 75 | Isafjordur | ISA-T | 65.572 | -22.4802 | 10 | 10 | 2019 | Inshore | AB |
| I1322T25 | Trawl | Isafjordur | 8 | 0 | 5.29 | 75 | Isafjordur | ISA-T | 65.572 | -22.4802 | 10 | 10 | 2019 | Inshore | AB |
| A980T18 | Trawl | Arnarfjordur | 9.6 | 0 | 8.78 | 58 | Outer_Arnarfjordur | Outer_ARN-T | 65.4243 | -23.322 | 2 | 10 | 2019 | Inshore | BB |
| A1076T12 | Trawl | Arnarfjordur | NA | 0 | NA | 49 | Inner_Arnarfjordur | Inner_ARN-T | 65.4422 | -23.2197 | 4 | 10 | 2019 | Inshore | NA |
| A1076T13 | Trawl | Arnarfjordur | NA | 0 | NA | 50 | Inner_Arnarfjordur | Inner_ARN-T | 65.4423 | -23.2198 | 4 | 10 | 2019 | Inshore | NA |
| A1076T14 | Trawl | Arnarfjordur | NA | 0 | NA | 51 | Inner_Arnarfjordur | Inner_ARN-T | 65.4424 | -23.2199 | 4 | 10 | 2019 | Inshore | NA |
| BS268BS38 | Seine | Strandir | NA | 0 | NA | 0 | Bassastadir | BAS-S | 65.8946 | -22.388 | 26 | 8 | 2019 | Inshore | NA |
| BS268BS48 | Seine | Strandir | NA | 0 | NA | 0 | Bassastadir | BAS-S | 65.8946 | -22.388 | 26 | 8 | 2019 | Inshore | NA |
| BS268BS67 | Seine | Strandir | NA | 0 | NA | 0 | Bassastadir | BAS-S | 65.8946 | -22.388 | 26 | 8 | 2019 | Inshore | NA |
| E10 | Seine | Strandir | NA | 0 | NA | 0 | Bassastadir | BAS-S | 65.8945 | -22.387 | 12 | 10 | 2019 | Inshore | NA |
| E100 | Seine | Arnarfjordur | NA | 0 | NA | 0 | Audkula | AUD-S | 65.96486 | -22.89687 | 11 | 10 | 2019 | Inshore | AB |
| I1322T8 | Trawl | Isafjordur | 8.1 | 0 | 5.5 | 75 | Isafjordur | ISA-T | 65.572 | -22.4802 | 10 | 10 | 2019 | Inshore | AB |
| I1322T9 | Trawl | Isafjordur | NA | 0 | NA | 47 | Isafjordur | ISA-T | 65.572 | -22.4802 | 10 | 10 | 2019 | Inshore | NA |
| IA268BS1 | Seine | Isafjordur | 6.4 | 0 | 2.5 | 0 | Arngerdareyri | ARG-S | 65.76263 | -21.67914 | 26 | 8 | 2019 | Inshore | AA |
| IA268BS10 | Seine | Isafjordur | 7.2 | 0 | 4.61 | 0 | Arngerdareyri | ARG-S | 65.76263 | -21.67914 | 26 | 8 | 2019 | Inshore | AB |
| E18 | Seine | Arnarfjordur | NA | 0 | NA | 0 | Audkula | AUD-S | 65.96486 | -22.89687 | 11 | 10 | 2019 | Inshore | AA |
| IA268BS11 | Seine | Isafjordur | 3.1 | 0 | 0.32 | 0 | Arngerdareyri | ARG-S | 65.76263 | -21.67914 | 26 | 8 | 2019 | Inshore | AB |
| E2 | Seine | Strandir | NA | 0 | NA | 0 | Bassastadir | BAS-S | 65.8945 | -22.387 | 12 | 10 | 2019 | Inshore | AA |
| E22 | Seine | Arnarfjordur | NA | 0 | NA | 0 | Audkula | AUD-S | 65.96486 | -22.89687 | 11 | 10 | 2019 | Inshore | AA |
| IA268BS12 | Seine | Isafjordur | 6.7 | 0 | 3.06 | 0 | Arngerdareyri | ARG-S | 65.76263 | -21.67914 | 26 | 8 | 2019 | Inshore | AB |
| IA268BS13 | Seine | Isafjordur | 5.7 | 0 | 2.02 | 0 | Arngerdareyri | ARG-S | 65.76263 | -21.67914 | 26 | 8 | 2019 | Inshore | AA |
| IA268BS14 | Seine | Isafjordur | 6.6 | 0 | 3.57 | 0 | Arngerdareyri | ARG-S | 65.76263 | -21.67914 | 26 | 8 | 2019 | Inshore | AB |
| IA268BS2 | Seine | Isafjordur | 7.4 | 0 | 4.77 | 0 | Arngerdareyri | ARG-S | 65.76263 | -21.67914 | 26 | 8 | 2019 | Inshore | AA |
| E27 | Seine | Arnarfjordur | NA | 0 | NA | 0 | Audkula | AUD-S | 65.96486 | -22.89687 | 11 | 10 | 2019 | Inshore | AA |
| IA268BS3 | Seine | Isafjordur | 6.1 | 0 | 2.41 | 0 | Arngerdareyri | ARG-S | 65.76263 | -21.67914 | 26 | 8 | 2019 | Inshore | AA |
| IA268BS4 | Seine | Isafjordur | 7 | 0 | 4.25 | 0 | Arngerdareyri | ARG-S | 65.76263 | -21.67914 | 26 | 8 | 2019 | Inshore | AB |
| E3 | Seine | Arnarfjordur | NA | 0 | NA | 0 | Audkula | AUD-S | 65.96486 | -22.89687 | 11 | 10 | 2019 | Inshore | AA |
| E30 | Seine | Strandir | NA | 0 | NA | 0 | Bassastadir | BAS-S | 65.8945 | -22.387 | 12 | 10 | 2019 | Inshore | AA |
| E31 | Seine | Arnarfjordur | NA | 0 | NA | 0 | Audkula | AUD-S | 65.96486 | -22.89687 | 11 | 10 | 2019 | Inshore | AA |
| E32 | Seine | Arnarfjordur | NA | 0 | NA | 0 | Audkula | AUD-S | 65.96486 | -22.89687 | 11 | 10 | 2019 | Inshore | AA |
| E33 | Seine | Strandir | NA | 0 | NA | 0 | Bassastadir | BAS-S | 65.8945 | -22.387 | 12 | 10 | 2019 | Inshore | AA |
| E34 | Seine | Strandir | NA | 0 | NA | 0 | Bassastadir | BAS-S | 65.8945 | -22.387 | 12 | 10 | 2019 | Inshore | AA |
| E36 | Seine | Strandir | NA | 0 | NA | 0 | Bassastadir | BAS-S | 65.8945 | -22.387 | 12 | 10 | 2019 | Inshore | AA |
| E37 | Seine | Strandir | NA | 0 | NA | 0 | Bassastadir | BAS-S | 65.8945 | -22.387 | 12 | 10 | 2019 | Inshore | AB |
| E38 | Seine | Arnarfjordur | NA | 0 | NA | 0 | Audkula | AUD-S | 65.96486 | -22.89687 | 11 | 10 | 2019 | Inshore | AA |
| E39 | Seine | Strandir | NA | 0 | NA | 0 | Bassastadir | BAS-S | 65.8945 | -22.387 | 12 | 10 | 2019 | Inshore | AB |
| E4 | Seine | Arnarfjordur | NA | 0 | NA | 0 | Audkula | AUD-S | 65.96485 | -22.89686 | 11 | 10 | 2019 | Inshore | NA |
| E40 | Seine | Arnarfjordur | NA | 0 | NA | 0 | Audkula | AUD-S | 65.96486 | -22.89687 | 11 | 10 | 2019 | Inshore | AA |
| E41B | Seine | Arnarfjordur | NA | 0 | NA | 0 | Audkula | AUD-S | 65.96486 | -22.89687 | 11 | 10 | 2019 | Inshore | AA |
| IA268BS5 | Seine | Isafjordur | 7.1 | 0 | 4.3 | 0 | Arngerdareyri | ARG-S | 65.76263 | -21.67914 | 26 | 8 | 2019 | Inshore | AA |
| E43 | Seine | Strandir | NA | 0 | NA | 0 | Bassastadir | BAS-S | 65.8945 | -22.387 | 12 | 10 | 2019 | Inshore | AA |
| E44 | Seine | Arnarfjordur | NA | 0 | NA | 0 | Audkula | AUD-S | 65.96486 | -22.89687 | 11 | 10 | 2019 | Inshore | AA |
| E46 | Seine | Arnarfjordur | NA | 0 | NA | 0 | Audkula | AUD-S | 65.96486 | -22.89687 | 11 | 10 | 2019 | Inshore | AB |
| E47 | Seine | Strandir | NA | 0 | NA | 0 | Bassastadir | BAS-S | 65.8945 | -22.387 | 12 | 10 | 2019 | Inshore | AA |
| E48 | Seine | Strandir | NA | 0 | NA | 0 | Bassastadir | BAS-S | 65.8945 | -22.387 | 12 | 10 | 2019 | Inshore | AA |
| E49 | Seine | Strandir | NA | 0 | NA | 0 | Bassastadir | BAS-S | 65.8945 | -22.387 | 12 | 10 | 2019 | Inshore | AA |
| E5 | Seine | Arnarfjordur | NA | 0 | NA | 0 | Audkula | AUD-S | 65.96486 | -22.89687 | 11 | 10 | 2019 | Inshore | AB |
| E50 | Seine | Arnarfjordur | NA | 0 | NA | 0 | Audkula | AUD-S | 65.96486 | -22.89687 | 11 | 10 | 2019 | Inshore | AA |
| IA268BS6 | Seine | Isafjordur | 4.1 | 0 | 0.75 | 0 | Arngerdareyri | ARG-S | 65.76263 | -21.67914 | 26 | 8 | 2019 | Inshore | AA |
| IA268BS7 | Seine | Isafjordur | 6.8 | 0 | 3.38 | 0 | Arngerdareyri | ARG-S | 65.76263 | -21.67914 | 26 | 8 | 2019 | Inshore | AA |
| E53 | Seine | Strandir | NA | 0 | NA | 0 | Bassastadir | BAS-S | 65.8945 | -22.387 | 12 | 10 | 2019 | Inshore | AA |
| E55 | Seine | Strandir | NA | 0 | NA | 0 | Bassastadir | BAS-S | 65.8945 | -22.387 | 12 | 10 | 2019 | Inshore | AA |
| E56 | Seine | Arnarfjordur | NA | 0 | NA | 0 | Audkula | AUD-S | 65.96486 | -22.89687 | 11 | 10 | 2019 | Inshore | AA |
| IA268BS8 | Seine | Isafjordur | 7 | 0 | 4.45 | 0 | Arngerdareyri | ARG-S | 65.76263 | -21.67914 | 26 | 8 | 2019 | Inshore | AA |
| E59 | Seine | Arnarfjordur | NA | 0 | NA | 0 | Audkula | AUD-S | 65.96486 | -22.89687 | 11 | 10 | 2019 | Inshore | AA |
| E6 | Seine | Arnarfjordur | NA | 0 | NA | 0 | Audkula | AUD-S | 65.96486 | -22.89687 | 11 | 10 | 2019 | Inshore | AB |
| E60 | Seine | Strandir | NA | 0 | NA | 0 | Bassastadir | BAS-S | 65.8945 | -22.387 | 12 | 10 | 2019 | Inshore | AA |
| IA268BS9 | Seine | Isafjordur | 5.6 | 0 | 1.96 | 0 | Arngerdareyri | ARG-S | 65.76263 | -21.67914 | 26 | 8 | 2019 | Inshore | AA |
| SF229BS11 | Seine | Isafjordur | 6.3 | 0 | 2.62 | 0 | Seydisfjordur | SEY-S | 65.95817 | -22.38883 | 22 | 9 | 2019 | Inshore | AA |
| SF229BS12 | Seine | Isafjordur | 5.3 | 0 | 1.44 | 0 | Seydisfjordur | SEY-S | 65.95817 | -22.38883 | 22 | 9 | 2019 | Inshore | AA |
| SF229BS13 | Seine | Isafjordur | 5.5 | 0 | 1.72 | 0 | Seydisfjordur | SEY-S | 65.95817 | -22.38883 | 22 | 9 | 2019 | Inshore | AA |
| E67 | Seine | Strandir | NA | 0 | NA | 0 | Bassastadir | BAS-S | 65.8945 | -22.387 | 12 | 10 | 2019 | Inshore | AA |
| E68 | Seine | Strandir | NA | 0 | NA | 0 | Bassastadir | BAS-S | 65.8945 | -22.387 | 12 | 10 | 2019 | Inshore | AA |
| E69 | Seine | Arnarfjordur | NA | 0 | NA | 0 | Audkula | AUD-S | 65.96486 | -22.89687 | 11 | 10 | 2019 | Inshore | AA |
| SF229BS14 | Seine | Isafjordur | 5.7 | 0 | 2.19 | 0 | Seydisfjordur | SEY-S | 65.95817 | -22.38883 | 22 | 9 | 2019 | Inshore | AA |
| E70 | Seine | Strandir | NA | 0 | NA | 0 | Bassastadir | BAS-S | 65.8945 | -22.387 | 12 | 10 | 2019 | Inshore | AA |
| E71 | Seine | Arnarfjordur | NA | 0 | NA | 0 | Audkula | AUD-S | 65.96486 | -22.89687 | 11 | 10 | 2019 | Inshore | AA |
| E72 | Seine | Arnarfjordur | NA | 0 | NA | 0 | Audkula | AUD-S | 65.96486 | -22.89687 | 11 | 10 | 2019 | Inshore | AA |
| E73 | Seine | Strandir | NA | 0 | NA | 0 | Bassastadir | BAS-S | 65.8945 | -22.387 | 12 | 10 | 2019 | Inshore | AA |
| E74 | Seine | Arnarfjordur | NA | 0 | NA | 0 | Audkula | AUD-S | 65.96486 | -22.89687 | 11 | 10 | 2019 | Inshore | AA |
| SF229BS15 | Seine | Isafjordur | 6.5 | 0 | 3.07 | 0 | Seydisfjordur | SEY-S | 65.95817 | -22.38883 | 22 | 9 | 2019 | Inshore | AA |
| E77 | Seine | Strandir | NA | 0 | NA | 0 | Bassastadir | BAS-S | 65.8945 | -22.387 | 12 | 10 | 2019 | Inshore | AA |
| E78 | Seine | Arnarfjordur | NA | 0 | NA | 0 | Audkula | AUD-S | 65.96486 | -22.89687 | 11 | 10 | 2019 | Inshore | AA |
| SF229BS16 | Seine | Isafjordur | 5.9 | 0 | 2.12 | 0 | Seydisfjordur | SEY-S | 65.95817 | -22.38883 | 22 | 9 | 2019 | Inshore | AA |
| E80 | Seine | Arnarfjordur | NA | 0 | NA | 0 | Audkula | AUD-S | 65.96486 | -22.89687 | 11 | 10 | 2019 | Inshore | AA |
| SF229BS17 | Seine | Isafjordur | 5.1 | 0 | 1.48 | 0 | Seydisfjordur | SEY-S | 65.95817 | -22.38883 | 22 | 9 | 2019 | Inshore | AA |
| SF229BS18 | Seine | Isafjordur | 6.6 | 0 | 3 | 0 | Seydisfjordur | SEY-S | 65.95817 | -22.38883 | 22 | 9 | 2019 | Inshore | AA |
| E83 | Seine | Strandir | NA | 0 | NA | 0 | Bassastadir | BAS-S | 65.8945 | -22.387 | 12 | 10 | 2019 | Inshore | AA |
| E84 | Seine | Strandir | NA | 0 | NA | 0 | Bassastadir | BAS-S | 65.8945 | -22.387 | 12 | 10 | 2019 | Inshore | AA |
| E85 | Seine | Strandir | NA | 0 | NA | 0 | Bassastadir | BAS-S | 65.8945 | -22.387 | 12 | 10 | 2019 | Inshore | AA |
| E86 | Seine | Arnarfjordur | NA | 0 | NA | 0 | Audkula | AUD-S | 65.96486 | -22.89687 | 11 | 10 | 2019 | Inshore | AA |
| E87 | Seine | Strandir | NA | 0 | NA | 0 | Bassastadir | BAS-S | 65.8945 | -22.387 | 12 | 10 | 2019 | Inshore | AA |
| E88 | Seine | Strandir | NA | 0 | NA | 0 | Bassastadir | BAS-S | 65.8945 | -22.387 | 12 | 10 | 2019 | Inshore | AA |
| E89 | Seine | Strandir | NA | 0 | NA | 0 | Bassastadir | BAS-S | 65.8945 | -22.387 | 12 | 10 | 2019 | Inshore | AA |
| SF229BS19 | Seine | Isafjordur | 5.6 | 0 | 1.77 | 0 | Seydisfjordur | SEY-S | 65.95817 | -22.38883 | 22 | 9 | 2019 | Inshore | AB |
| E90 | Seine | Arnarfjordur | NA | 0 | NA | 0 | Audkula | AUD-S | 65.96486 | -22.89687 | 11 | 10 | 2019 | Inshore | AA |
| E91 | Seine | Strandir | NA | 0 | NA | 0 | Bassastadir | BAS-S | 65.8945 | -22.387 | 12 | 10 | 2019 | Inshore | AA |
| E92 | Seine | Strandir | NA | 0 | NA | 0 | Bassastadir | BAS-S | 65.8945 | -22.387 | 12 | 10 | 2019 | Inshore | AA |
| E93 | Seine | Arnarfjordur | NA | 0 | NA | 0 | Audkula | AUD-S | 65.96486 | -22.89687 | 11 | 10 | 2019 | Inshore | AA |
| E94 | Seine | Strandir | NA | 0 | NA | 0 | Bassastadir | BAS-S | 65.8945 | -22.387 | 12 | 10 | 2019 | Inshore | AB |
| E96 | Seine | Strandir | NA | 0 | NA | 0 | Bassastadir | BAS-S | 65.8945 | -22.387 | 12 | 10 | 2019 | Inshore | AB |
| E97 | Seine | Strandir | NA | 0 | NA | 0 | Bassastadir | BAS-S | 65.8945 | -22.387 | 12 | 10 | 2019 | Inshore | AA |
| E98 | Seine | Arnarfjordur | NA | 0 | NA | 0 | Audkula | AUD-S | 65.96486 | -22.89687 | 11 | 10 | 2019 | Inshore | AA |
| E99 | Seine | Arnarfjordur | NA | 1 | NA | 1 | Audkula | AUD-S | 65.96487 | -22.89688 | 11 | 10 | 2019 | Inshore | NA |
| SF229BS2 | Seine | Isafjordur | 6.3 | 0 | 2.49 | 0 | Seydisfjordur | SEY-S | 65.95817 | -22.38883 | 22 | 9 | 2019 | Inshore | AA |
| SF229BS20 | Seine | Isafjordur | 5.6 | 0 | 1.64 | 0 | Seydisfjordur | SEY-S | 65.95817 | -22.38883 | 22 | 9 | 2019 | Inshore | AA |
| SF229BS21 | Seine | Isafjordur | 6.6 | 0 | 2.8 | 0 | Seydisfjordur | SEY-S | 65.95817 | -22.38883 | 22 | 9 | 2019 | Inshore | AA |
| SF229BS22 | Seine | Isafjordur | 6.8 | 0 | 3.35 | 0 | Seydisfjordur | SEY-S | 65.95817 | -22.38883 | 22 | 9 | 2019 | Inshore | AA |
| SF229BS23 | Seine | Isafjordur | 5.6 | 0 | 1.86 | 0 | Seydisfjordur | SEY-S | 65.95817 | -22.38883 | 22 | 9 | 2019 | Inshore | AA |
| SF229BS3 | Seine | Isafjordur | 5.1 | 0 | 1.29 | 0 | Seydisfjordur | SEY-S | 65.95817 | -22.38883 | 22 | 9 | 2019 | Inshore | AA |
| SF229BS4 | Seine | Isafjordur | 5 | 0 | 1.3 | 0 | Seydisfjordur | SEY-S | 65.95817 | -22.38883 | 22 | 9 | 2019 | Inshore | AA |
| SF229BS5 | Seine | Isafjordur | 5.8 | 0 | 2.22 | 0 | Seydisfjordur | SEY-S | 65.95817 | -22.38883 | 22 | 9 | 2019 | Inshore | AA |
| SF229BS6 | Seine | Isafjordur | 5.9 | 0 | 2.18 | 0 | Seydisfjordur | SEY-S | 65.95817 | -22.38883 | 22 | 9 | 2019 | Inshore | AA |
| SF229BS7 | Seine | Isafjordur | 5.2 | 0 | 1.4 | 0 | Seydisfjordur | SEY-S | 65.95817 | -22.38883 | 22 | 9 | 2019 | Inshore | AA |
| SF229BS8 | Seine | Isafjordur | 6.1 | 0 | 2.45 | 0 | Seydisfjordur | SEY-S | 65.95817 | -22.38883 | 22 | 9 | 2019 | Inshore | AA |
| SF229BS9 | Seine | Isafjordur | 6 | 0 | 2.37 | 0 | Seydisfjordur | SEY-S | 65.95817 | -22.38883 | 22 | 9 | 2019 | Inshore | AA |
| SF59BS1 | Seine | Isafjordur | NA | 0 | NA | 0 | Seydisfjordur | SEY-S | 65.95818 | -22.38884 | 22 | 9 | 2019 | Inshore | NA |
| SF59BS11 | Seine | Isafjordur | NA | 0 | NA | 0 | Seydisfjordur | SEY-S | 65.95818 | -22.38884 | 5 | 9 | 2019 | Inshore | NA |
| SF59BS12 | Seine | Isafjordur | NA | 0 | NA | 0 | Seydisfjordur | SEY-S | 65.95819 | -22.38885 | 5 | 9 | 2019 | Inshore | NA |
| SF59BS13 | Seine | Isafjordur | NA | 0 | NA | 0 | Seydisfjordur | SEY-S | 65.95820 | -22.38886 | 5 | 9 | 2019 | Inshore | NA |
| SF59BS14 | Seine | Isafjordur | 5.3 | 0 | 1.5 | 0 | Seydisfjordur | SEY-S | 65.95821 | -22.38887 | 5 | 9 | 2019 | Inshore | AB |
| SF59BS15 | Seine | Isafjordur | NA | 0 | NA | 0 | Seydisfjordur | SEY-S | 65.95822 | -22.38888 | 5 | 9 | 2019 | Inshore | NA |
| SF59BS16 | Seine | Isafjordur | 5 | 0 | 1.11 | 0 | Seydisfjordur | SEY-S | 65.95817 | -22.38883 | 5 | 9 | 2019 | Inshore | AA |
| SF59BS17 | Seine | Isafjordur | 4.1 | 0 | 0.65 | 0 | Seydisfjordur | SEY-S | 65.95817 | -22.38883 | 5 | 9 | 2019 | Inshore | AA |
| SF59BS18 | Seine | Isafjordur | 4.9 | 0 | 1.07 | 0 | Seydisfjordur | SEY-S | 65.95817 | -22.38883 | 5 | 9 | 2019 | Inshore | AA |
| SF59BS19 | Seine | Isafjordur | 4 | 0 | 0.54 | 0 | Seydisfjordur | SEY-S | 65.95817 | -22.38883 | 5 | 9 | 2019 | Inshore | AA |
| SF59BS21 | Seine | Isafjordur | 5.7 | 0 | 2.06 | 0 | Seydisfjordur | SEY-S | 65.95819 | -22.38885 | 5 | 9 | 2019 | Inshore | AA |
| SF59BS22 | Seine | Isafjordur | 3.9 | 0 | 0.41 | 0 | Seydisfjordur | SEY-S | 65.95817 | -22.38883 | 5 | 9 | 2019 | Inshore | AA |
| SF59BS23 | Seine | Isafjordur | NA | 0 | NA | 0 | Seydisfjordur | SEY-S | 65.95818 | -22.38884 | 5 | 9 | 2019 | Inshore | NA |
| SF59BS24 | Seine | Isafjordur | NA | 0 | NA | 0 | Seydisfjordur | SEY-S | 65.95819 | -22.38885 | 5 | 9 | 2019 | Inshore | NA |
| SF59BS25 | Seine | Isafjordur | NA | 0 | NA | 0 | Seydisfjordur | SEY-S | 65.95820 | -22.38886 | 5 | 9 | 2019 | Inshore | NA |
| SF59BS26 | Seine | Isafjordur | NA | 0 | NA | 0 | Seydisfjordur | SEY-S | 65.95821 | -22.38887 | 5 | 9 | 2019 | Inshore | NA |
| SF59BS6 | Seine | Isafjordur | 5 | 0 | 0.96 | 0 | Seydisfjordur | SEY-S | 65.95817 | -22.38883 | 5 | 9 | 2019 | Inshore | AB |
| SF59BS7 | Seine | Isafjordur | NA | 0 | NA | 0 | Seydisfjordur | SEY-S | 65.95818 | -22.38884 | 5 | 9 | 2019 | Inshore | NA |
| SF59BS9 | Seine | Isafjordur | 4.6 | 0 | 0.93 | 0 | Seydisfjordur | SEY-S | 65.95817 | -22.38883 | 5 | 9 | 2019 | Inshore | AA |
| 021177C35 | Trawl | Isafjordur | 10 | 0 | 9.7 | 43 | Mjoifjordur | MJO-T | 65.97533 | -22.55733 | 2 | 11 | 2017 | NA | BB |
| 021178C23 | Trawl | Isafjordur | 10 | 0 | 9.73 | 33 | Isafjordur | ISA-T | 65.9655 | -22.45833333 | 2 | 11 | 2017 | NA | AB |
| 0711106C47 | Trawl | Isafjordur | 10.1 | 0 | 11.08 | 42 | Isafjordur | ISA-T | 65.92267 | -22.4075 | 7 | 11 | 2017 | NA | BB |
| 0711106C29 | Trawl | Isafjordur | 10.2 | 0 | 10.8 | 42 | Isafjordur | ISA-T | 65.92267 | -22.4075 | 7 | 11 | 2017 | NA | BB |
| 0711106C44 | Trawl | Isafjordur | 10.2 | 0 | 10.86 | 42 | Isafjordur | ISA-T | 65.92267 | -22.4075 | 7 | 11 | 2017 | NA | BB |
| 0711106C26 | Trawl | Isafjordur | 10.2 | 0 | 11 | 42 | Isafjordur | ISA-T | 65.92267 | -22.4075 | 7 | 11 | 2017 | NA | BB |
| 0711106C23 | Trawl | Isafjordur | 10.4 | 0 | 11.04 | 42 | Isafjordur | ISA-T | 65.92267 | -22.4075 | 7 | 11 | 2017 | NA | BB |
| 0711106C38 | Trawl | Isafjordur | 10.4 | 0 | 9.84 | 42 | Isafjordur | ISA-T | 65.92267 | -22.4075 | 7 | 11 | 2017 | NA | BB |
| A1072T2 | Trawl | Arnarfjordur | 10.5 | 0 | 9.92 | 63 | Inner_Arnarfjordur | Inner_ARN-T | 65.4476 | -23.185 | 4 | 10 | 2019 | NA | AB |
| 021177C18 | Trawl | Isafjordur | 10.5 | 0 | 11.69 | 43 | Mjoifjordur | MJO-T | 65.97533 | -22.55733 | 2 | 11 | 2017 | NA | BB |
| 0711106C28 | Trawl | Isafjordur | 10.5 | 0 | 12.24 | 42 | Isafjordur | ISA-T | 65.92267 | -22.4075 | 7 | 11 | 2017 | NA | BB |
| 0711106C34 | Trawl | Isafjordur | 10.5 | 0 | 12.78 | 42 | Isafjordur | ISA-T | 65.92267 | -22.4075 | 7 | 11 | 2017 | NA | BB |
| 011167C11 | Trawl | Isafjordur | 10.5 | 0 | 8.58 | 48 | Isafjordur | ISA-T | 65.92233 | -22.4065 | 1 | 11 | 2017 | NA | AA |
| 0711106C45 | Trawl | Isafjordur | 10.6 | 0 | 11.27 | 42 | Isafjordur | ISA-T | 65.92267 | -22.4075 | 7 | 11 | 2017 | NA | BB |
| 0711106C25 | Trawl | Isafjordur | 10.6 | 0 | 11.74 | 42 | Isafjordur | ISA-T | 65.92267 | -22.4075 | 7 | 11 | 2017 | NA | BB |
| 0711106C30 | Trawl | Isafjordur | 10.6 | 0 | 11.88 | 42 | Isafjordur | ISA-T | 65.92267 | -22.4075 | 7 | 11 | 2017 | NA | BB |
| 0711106C37 | Trawl | Isafjordur | 10.6 | 0 | 13.38 | 42 | Isafjordur | ISA-T | 65.92267 | -22.4075 | 7 | 11 | 2017 | NA | BB |
| 021178C5 | Trawl | Isafjordur | 10.7 | 0 | 10.34 | 33 | Isafjordur | ISA-T | 65.9655 | -22.45833333 | 2 | 11 | 2017 | NA | AB |
| 0711106C51 | Trawl | Isafjordur | 10.7 | 0 | 11.24 | 42 | Isafjordur | ISA-T | 65.92267 | -22.4075 | 7 | 11 | 2017 | NA | BB |
| 021178C10 | Trawl | Isafjordur | 10.7 | 0 | 11.59 | 33 | Isafjordur | ISA-T | 65.9655 | -22.45833333 | 2 | 11 | 2017 | NA | AB |
| 021177C36 | Trawl | Isafjordur | 10.7 | 0 | 12.21 | 43 | Mjoifjordur | MJO-T | 65.97533 | -22.55733 | 2 | 11 | 2017 | NA | AB |
| 021178C24 | Trawl | Isafjordur | 10.8 | 0 | 13.38 | 33 | Isafjordur | ISA-T | 65.9655 | -22.45833333 | 2 | 11 | 2017 | NA | AB |
| 021177C30 | Trawl | Isafjordur | 11 | 0 | 11.94 | 43 | Mjoifjordur | MJO-T | 65.97533 | -22.55733 | 2 | 11 | 2017 | NA | BB |
| 011167C16 | Trawl | Isafjordur | 11 | 0 | 13.08 | 48 | Isafjordur | ISA-T | 65.92233 | -22.4065 | 1 | 11 | 2017 | NA | AB |
| 0711106C39 | Trawl | Isafjordur | 11 | 0 | 13.21 | 42 | Isafjordur | ISA-T | 65.92267 | -22.4075 | 7 | 11 | 2017 | NA | BB |
| 021178C11 | Trawl | Isafjordur | 11 | 0 | 15.15 | 33 | Isafjordur | ISA-T | 65.9655 | -22.45833333 | 2 | 11 | 2017 | NA | AB |
| 021177C28 | Trawl | Isafjordur | 11 | 0 | 18 | 43 | Mjoifjordur | MJO-T | 65.97533 | -22.55733 | 2 | 11 | 2017 | NA | AB |
| 011167C12 | Trawl | Isafjordur | 11.1 | 0 | 10.2 | 48 | Isafjordur | ISA-T | 65.92233 | -22.4065 | 1 | 11 | 2017 | NA | BB |
| 021177C37 | Trawl | Isafjordur | 11.1 | 0 | 13.49 | 43 | Mjoifjordur | MJO-T | 65.97533 | -22.55733 | 2 | 11 | 2017 | NA | AB |
| 0711106C24 | Trawl | Isafjordur | 11.1 | 0 | 13.73 | 42 | Isafjordur | ISA-T | 65.92267 | -22.4075 | 7 | 11 | 2017 | NA | BB |
| 011167C15 | Trawl | Isafjordur | 11.1 | 0 | 14.47 | 48 | Isafjordur | ISA-T | 65.92233 | -22.4065 | 1 | 11 | 2017 | NA | AB |
| 0711106C35 | Trawl | Isafjordur | 11.1 | 0 | 14.87 | 42 | Isafjordur | ISA-T | 65.92267 | -22.4075 | 7 | 11 | 2017 | NA | BB |
| 021178C31 | Trawl | Isafjordur | 11.2 | 0 | 14.54 | 33 | Isafjordur | ISA-T | 65.9655 | -22.45833333 | 2 | 11 | 2017 | NA | AB |
| 0711106C54 | Trawl | Isafjordur | 11.3 | 0 | 13.61 | 42 | Isafjordur | ISA-T | 65.92267 | -22.4075 | 7 | 11 | 2017 | NA | AB |
| 0711106C43 | Trawl | Isafjordur | 11.4 | 0 | 13.85 | 42 | Isafjordur | ISA-T | 65.92267 | -22.4075 | 7 | 11 | 2017 | NA | BB |
| 0711106C21 | Trawl | Isafjordur | 11.4 | 0 | 15.59 | 42 | Isafjordur | ISA-T | 65.92267 | -22.4075 | 7 | 11 | 2017 | NA | BB |
| 021177C12 | Trawl | Isafjordur | 11.4 | 0 | 16.28 | 43 | Mjoifjordur | MJO-T | 65.97533 | -22.55733 | 2 | 11 | 2017 | NA | AB |
| 011167C7 | Trawl | Isafjordur | 11.4 | 0 | 18.6 | 48 | Isafjordur | ISA-T | 65.92233 | -22.4065 | 1 | 11 | 2017 | NA | AA |
| 0711106C46 | Trawl | Isafjordur | 11.5 | 0 | 15.85 | 42 | Isafjordur | ISA-T | 65.92267 | -22.4075 | 7 | 11 | 2017 | NA | BB |
| 0711106C18 | Trawl | Isafjordur | 11.5 | 0 | 16.06 | 42 | Isafjordur | ISA-T | 65.92267 | -22.4075 | 7 | 11 | 2017 | NA | BB |
| 0711106C41 | Trawl | Isafjordur | 11.5 | 0 | 16.67 | 42 | Isafjordur | ISA-T | 65.92267 | -22.4075 | 7 | 11 | 2017 | NA | BB |
| 0711106C22 | Trawl | Isafjordur | 11.6 | 0 | 15.2 | 42 | Isafjordur | ISA-T | 65.92267 | -22.4075 | 7 | 11 | 2017 | NA | BB |
| 021178C29 | Trawl | Isafjordur | 11.6 | 0 | 16.71 | 33 | Isafjordur | ISA-T | 65.9655 | -22.45833333 | 2 | 11 | 2017 | NA | AB |
| 021178C2 | Trawl | Isafjordur | 11.6 | 0 | 17.14 | 33 | Isafjordur | ISA-T | 65.9655 | -22.45833333 | 2 | 11 | 2017 | NA | AA |
| 0711106C13 | Trawl | Isafjordur | 11.6 | 0 | 17.78 | 42 | Isafjordur | ISA-T | 65.92267 | -22.4075 | 7 | 11 | 2017 | NA | BB |
| 021178C3 | Trawl | Isafjordur | 11.7 | 0 | 16.4 | 33 | Isafjordur | ISA-T | 65.9655 | -22.45833333 | 2 | 11 | 2017 | NA | BB |
| 0711106C42 | Trawl | Isafjordur | 11.8 | 0 | 15.52 | 42 | Isafjordur | ISA-T | 65.92267 | -22.4075 | 7 | 11 | 2017 | NA | BB |
| 011167C9 | Trawl | Isafjordur | 11.8 | 0 | 15.78 | 48 | Isafjordur | ISA-T | 65.92233 | -22.4065 | 1 | 11 | 2017 | NA | AB |
| 021178C4 | Trawl | Isafjordur | 11.8 | 0 | 16.28 | 33 | Isafjordur | ISA-T | 65.9655 | -22.45833333 | 2 | 11 | 2017 | NA | BB |
| 0711106C48 | Trawl | Isafjordur | 11.8 | 0 | 16.38 | 42 | Isafjordur | ISA-T | 65.92267 | -22.4075 | 7 | 11 | 2017 | NA | BB |
| 021178C12 | Trawl | Isafjordur | 11.8 | 0 | 17.46 | 33 | Isafjordur | ISA-T | 65.9655 | -22.45833333 | 2 | 11 | 2017 | NA | AA |
| 0711106C4 | Trawl | Isafjordur | 11.9 | 0 | 18.93 | 42 | Isafjordur | ISA-T | 65.92267 | -22.4075 | 7 | 11 | 2017 | NA | AB |
| 021178C20 | Trawl | Isafjordur | 11.9 | 0 | 20.6 | 33 | Isafjordur | ISA-T | 65.9655 | -22.45833333 | 2 | 11 | 2017 | NA | BB |
| 021177C38 | Trawl | Isafjordur | 12 | 0 | 17.52 | 43 | Mjoifjordur | MJO-T | 65.97533 | -22.55733 | 2 | 11 | 2017 | NA | AB |
| 011167C2 | Trawl | Isafjordur | 12.1 | 0 | 17.73 | 48 | Isafjordur | ISA-T | 65.92233 | -22.4065 | 1 | 11 | 2017 | NA | AB |
| 021178C33 | Trawl | Isafjordur | 12.1 | 0 | 19.4 | 33 | Isafjordur | ISA-T | 65.9655 | -22.45833333 | 2 | 11 | 2017 | NA | AB |
| 011167C1 | Trawl | Isafjordur | 12.1 | 0 | 20.16 | 48 | Isafjordur | ISA-T | 65.92233 | -22.4065 | 1 | 11 | 2017 | NA | AB |
| 021178C1 | Trawl | Isafjordur | 12.2 | 0 | 18.03 | 33 | Isafjordur | ISA-T | 65.9655 | -22.45833333 | 2 | 11 | 2017 | NA | AB |
| 011167C20 | Trawl | Isafjordur | 12.2 | 0 | 18.84 | 48 | Isafjordur | ISA-T | 65.92233 | -22.4065 | 1 | 11 | 2017 | NA | BB |
| 021177C23 | Trawl | Isafjordur | 12.2 | 0 | 21.77 | 43 | Mjoifjordur | MJO-T | 65.97533 | -22.55733 | 2 | 11 | 2017 | NA | BB |
| 021178C19 | Trawl | Isafjordur | 12.2 | 0 | 22.87 | 33 | Isafjordur | ISA-T | 65.9655 | -22.45833333 | 2 | 11 | 2017 | NA | AB |
| 021177C16 | Trawl | Isafjordur | 12.4 | 0 | 21.88 | 43 | Mjoifjordur | MJO-T | 65.97533 | -22.55733 | 2 | 11 | 2017 | NA | BB |
| 0711106C15 | Trawl | Isafjordur | 12.5 | 0 | 20.35 | 42 | Isafjordur | ISA-T | 65.92267 | -22.4075 | 7 | 11 | 2017 | NA | BB |
| 0711106C10 | Trawl | Isafjordur | 12.5 | 0 | 23.12 | 42 | Isafjordur | ISA-T | 65.92267 | -22.4075 | 7 | 11 | 2017 | NA | AB |
| 021178C8 | Trawl | Isafjordur | 12.7 | 0 | 23.16 | 33 | Isafjordur | ISA-T | 65.9655 | -22.45833333 | 2 | 11 | 2017 | NA | AA |
| 021178C18 | Trawl | Isafjordur | 12.8 | 0 | 24.09 | 33 | Isafjordur | ISA-T | 65.9655 | -22.45833333 | 2 | 11 | 2017 | NA | BB |
| 021177C4 | Trawl | Isafjordur | 12.8 | 0 | 25.81 | 43 | Mjoifjordur | MJO-T | 65.97533 | -22.55733 | 2 | 11 | 2017 | NA | AB |
| 021177C22 | Trawl | Isafjordur | 12.9 | 0 | 25.45 | 43 | Mjoifjordur | MJO-T | 65.97533 | -22.55733 | 2 | 11 | 2017 | NA | AB |
| 021178C21 | Trawl | Isafjordur | 12.9 | 0 | 25.69 | 33 | Isafjordur | ISA-T | 65.9655 | -22.45833333 | 2 | 11 | 2017 | NA | BB |
| 0711106C17 | Trawl | Isafjordur | 12.9 | 0 | 26 | 42 | Isafjordur | ISA-T | 65.92267 | -22.4075 | 7 | 11 | 2017 | NA | BB |
| 021178C6 | Trawl | Isafjordur | 12.9 | 0 | 29.7 | 33 | Isafjordur | ISA-T | 65.9655 | -22.45833333 | 2 | 11 | 2017 | NA | AA |
| 021177C5 | Trawl | Isafjordur | 13 | 0 | 26.57 | 43 | Mjoifjordur | MJO-T | 65.97533 | -22.55733 | 2 | 11 | 2017 | NA | AB |
| 021178C17 | Trawl | Isafjordur | 13 | 0 | 27.45 | 33 | Isafjordur | ISA-T | 65.9655 | -22.45833333 | 2 | 11 | 2017 | NA | AA |
| 0711106C20 | Trawl | Isafjordur | 13.1 | 0 | 24.76 | 42 | Isafjordur | ISA-T | 65.92267 | -22.4075 | 7 | 11 | 2017 | NA | BB |
| 021177C34 | Trawl | Isafjordur | 13.2 | 0 | 23.54 | 43 | Mjoifjordur | MJO-T | 65.97533 | -22.55733 | 2 | 11 | 2017 | NA | BB |
| 021178C9 | Trawl | Isafjordur | 13.3 | 0 | 23.65 | 33 | Isafjordur | ISA-T | 65.9655 | -22.45833333 | 2 | 11 | 2017 | NA | AB |
| 0711106C3 | Trawl | Isafjordur | 13.3 | 0 | 25.53 | 42 | Isafjordur | ISA-T | 65.92267 | -22.4075 | 7 | 11 | 2017 | NA | BB |
| 021178C13 | Trawl | Isafjordur | 13.3 | 0 | 29.98 | 33 | Isafjordur | ISA-T | 65.9655 | -22.45833333 | 2 | 11 | 2017 | NA | AB |
| 021177C11 | Trawl | Isafjordur | 13.3 | 0 | 31.4 | 43 | Mjoifjordur | MJO-T | 65.97533 | -22.55733 | 2 | 11 | 2017 | NA | AB |
| 021177C33 | Trawl | Isafjordur | 13.5 | 0 | 28.28 | 43 | Mjoifjordur | MJO-T | 65.97533 | -22.55733 | 2 | 11 | 2017 | NA | AB |
| 0711106C6 | Trawl | Isafjordur | 13.5 | 0 | 28.94 | 42 | Isafjordur | ISA-T | 65.92267 | -22.4075 | 7 | 11 | 2017 | NA | AB |
| 0711106C11 | Trawl | Isafjordur | 13.6 | 0 | 26.8 | 42 | Isafjordur | ISA-T | 65.92267 | -22.4075 | 7 | 11 | 2017 | NA | AB |
| 021178C14 | Trawl | Isafjordur | 13.6 | 0 | 30.42 | 33 | Isafjordur | ISA-T | 65.9655 | -22.45833333 | 2 | 11 | 2017 | NA | BB |
| 0711106C5 | Trawl | Isafjordur | 13.6 | 0 | 30.47 | 42 | Isafjordur | ISA-T | 65.92267 | -22.4075 | 7 | 11 | 2017 | NA | AB |
| 0711106C7 | Trawl | Isafjordur | 13.7 | 0 | 25.32 | 42 | Isafjordur | ISA-T | 65.92267 | -22.4075 | 7 | 11 | 2017 | NA | BB |
| 0711106C9 | Trawl | Isafjordur | 13.7 | 0 | 27.56 | 42 | Isafjordur | ISA-T | 65.92267 | -22.4075 | 7 | 11 | 2017 | NA | BB |
| 021178C27 | Trawl | Isafjordur | 13.7 | 0 | 28.53 | 33 | Isafjordur | ISA-T | 65.9655 | -22.45833333 | 2 | 11 | 2017 | NA | BB |
| 021177C2 | Trawl | Isafjordur | 13.7 | 0 | 30.99 | 43 | Mjoifjordur | MJO-T | 65.97533 | -22.55733 | 2 | 11 | 2017 | NA | AB |
| 021177C19 | Trawl | Isafjordur | 13.9 | 0 | 30.05 | 43 | Mjoifjordur | MJO-T | 65.97533 | -22.55733 | 2 | 11 | 2017 | NA | BB |
| 021177C3 | Trawl | Isafjordur | 13.9 | 0 | 33.24 | 43 | Mjoifjordur | MJO-T | 65.97533 | -22.55733 | 2 | 11 | 2017 | NA | AB |
| 0711106C12 | Trawl | Isafjordur | 13.9 | 0 | 63.45 | 42 | Isafjordur | ISA-T | 65.92267 | -22.4075 | 7 | 11 | 2017 | NA | BB |
| 021177C7 | Trawl | Isafjordur | 14 | 1 | 31.01 | 43 | Mjoifjordur | MJO-T | 65.97533 | -22.55733 | 2 | 11 | 2017 | NA | BB |
| 021177C15 | Trawl | Isafjordur | 14 | 1 | 34.12 | 43 | Mjoifjordur | MJO-T | 65.97533 | -22.55733 | 2 | 11 | 2017 | NA | AB |
| 021177C31 | Trawl | Isafjordur | 14.1 | 1 | 35 | 43 | Mjoifjordur | MJO-T | 65.97533 | -22.55733 | 2 | 11 | 2017 | NA | AB |
| 021177C1 | Trawl | Isafjordur | 14.2 | 1 | 33.5 | 43 | Mjoifjordur | MJO-T | 65.97533 | -22.55733 | 2 | 11 | 2017 | NA | AB |
| 0711106C16 | Trawl | Isafjordur | 14.2 | 1 | 35.27 | 42 | Isafjordur | ISA-T | 65.92267 | -22.4075 | 7 | 11 | 2017 | NA | BB |
| 021177C6 | Trawl | Isafjordur | 14.3 | 1 | 31.18 | 43 | Mjoifjordur | MJO-T | 65.97533 | -22.55733 | 2 | 11 | 2017 | NA | AA |
| 021177C25 | Trawl | Isafjordur | 14.3 | 1 | 36.36 | 43 | Mjoifjordur | MJO-T | 65.97533 | -22.55733 | 2 | 11 | 2017 | NA | BB |
| 0711106C14 | Trawl | Isafjordur | 14.3 | 1 | 36.99 | 42 | Isafjordur | ISA-T | 65.92267 | -22.4075 | 7 | 11 | 2017 | NA | AB |
| 0711106C19 | Trawl | Isafjordur | 14.3 | 1 | 37.56 | 42 | Isafjordur | ISA-T | 65.92267 | -22.4075 | 7 | 11 | 2017 | NA | BB |
| 0711106C1 | Trawl | Isafjordur | 14.4 | 1 | 38.46 | 42 | Isafjordur | ISA-T | 65.92267 | -22.4075 | 7 | 11 | 2017 | NA | AB |
| 021177C27 | Trawl | Isafjordur | 14.5 | 1 | 35.39 | 43 | Mjoifjordur | MJO-T | 65.97533 | -22.55733 | 2 | 11 | 2017 | NA | AB |
| 021177C10 | Trawl | Isafjordur | 14.5 | 1 | 36.04 | 43 | Mjoifjordur | MJO-T | 65.97533 | -22.55733 | 2 | 11 | 2017 | NA | AB |
| 021177C20 | Trawl | Isafjordur | 14.9 | 1 | 35.27 | 43 | Mjoifjordur | MJO-T | 65.97533 | -22.55733 | 2 | 11 | 2017 | NA | AB |
| 021177C24 | Trawl | Isafjordur | 15.1 | 1 | 40.46 | 43 | Mjoifjordur | MJO-T | 65.97533 | -22.55733 | 2 | 11 | 2017 | NA | BB |
| 021177C8 | Trawl | Isafjordur | 15.1 | 1 | 42.23 | 43 | Mjoifjordur | MJO-T | 65.97533 | -22.55733 | 2 | 11 | 2017 | NA | AB |
| 021177C17 | Trawl | Isafjordur | 15.1 | 1 | 44.39 | 43 | Mjoifjordur | MJO-T | 65.97533 | -22.55733 | 2 | 11 | 2017 | NA | AB |
| 0711106C8 | Trawl | Isafjordur | 15.3 | 1 | 41.76 | 42 | Isafjordur | ISA-T | 65.92267 | -22.4075 | 7 | 11 | 2017 | NA | BB |
| 021177C9 | Trawl | Isafjordur | 15.5 | 1 | 50.97 | 43 | Mjoifjordur | MJO-T | 65.97533 | -22.55733 | 2 | 11 | 2017 | NA | AB |
| 021177C21 | Trawl | Isafjordur | 15.6 | 1 | 42.65 | 43 | Mjoifjordur | MJO-T | 65.97533 | -22.55733 | 2 | 11 | 2017 | NA | BB |
| 021178C22 | Trawl | Isafjordur | 15.7 | 1 | 51.14 | 33 | Isafjordur | ISA-T | 65.9655 | -22.45833333 | 2 | 11 | 2017 | NA | BB |
| 2508EYC1 | Seine | Strandir | 16.2 | 1 | 67.01 | 0 | Eyjar | EYJ-S | 65.75949 | -23.47852 | 25 | 8 | 2017 | NA | AB |
| 021177C26 | Trawl | Isafjordur | 16.3 | 1 | 55.68 | 43 | Mjoifjordur | MJO-T | 65.97533 | -22.55733 | 2 | 11 | 2017 | NA | AB |
| 021178C15 | Trawl | Isafjordur | 17.5 | 1 | 65.04 | 33 | Isafjordur | ISA-T | 65.9655 | -22.45833333 | 2 | 11 | 2017 | NA | BB |
| 0711106C2 | Trawl | Isafjordur | 17.7 | 1 | 88.99 | 42 | Isafjordur | ISA-T | 65.92267 | -22.4075 | 7 | 11 | 2017 | NA | AB |
| 021178C30 | Trawl | Isafjordur | 18 | 1 | 28.25 | 33 | Isafjordur | ISA-T | 65.9655 | -22.45833333 | 2 | 11 | 2017 | NA | BB |
| A937T9 | Trawl | Arnarfjordur | 19.8 | 1 | 108.82 | 59 | Outer_Arnarfjordur | Outer_ARN-T | 65.8044444 | -23.9405555 | 1 | 10 | 2019 | NA | AA |
| A937T2 | Trawl | Arnarfjordur | 19.9 | 1 | 83.72 | 59 | Outer_Arnarfjordur | Outer_ARN-T | 65.8044444 | -23.9405555 | 1 | 10 | 2019 | NA | AB |
| 2508EYC2 | Seine | Strandir | 19.9 | 1 | 91.53 | 0 | Eyjar | EYJ-S | 65.75949 | -23.47852 | 25 | 8 | 2017 | NA | AB |
| BS268BS58 | Seine | Strandir | 2.8 | 0 | 0.21 | 0 | Bassastadir | BAS-S | 65.8945 | -22.387 | 26 | 8 | 2019 | NA | AA |
| BS268BS12 | Seine | Strandir | 2.8 | 0 | 0.22 | 0 | Bassastadir | BAS-S | 65.8945 | -22.387 | 26 | 8 | 2019 | NA | AA |
| A937T23 | Trawl | Arnarfjordur | 20 | 1 | 105.73 | 59 | Outer_Arnarfjordur | Outer_ARN-T | 65.8044444 | -23.9405555 | 1 | 10 | 2019 | NA | AB |
| 021178C25 | Trawl | Isafjordur | 20.3 | 1 | 105.13 | 33 | Isafjordur | ISA-T | 65.9655 | -22.45833333 | 2 | 11 | 2017 | NA | AB |
| A937T7 | Trawl | Arnarfjordur | 20.5 | 1 | 103.53 | 59 | Outer_Arnarfjordur | Outer_ARN-T | 65.8044444 | -23.9405555 | 1 | 10 | 2019 | NA | AB |
| A937T24 | Trawl | Arnarfjordur | 20.5 | 1 | 111.76 | 59 | Outer_Arnarfjordur | Outer_ARN-T | 65.8044444 | -23.9405555 | 1 | 10 | 2019 | NA | AA |
| A937T5 | Trawl | Arnarfjordur | 20.5 | 1 | 92.51 | 59 | Outer_Arnarfjordur | Outer_ARN-T | 65.8044444 | -23.9405555 | 1 | 10 | 2019 | NA | AB |
| A937T19 | Trawl | Arnarfjordur | 20.6 | 1 | 111.61 | 59 | Outer_Arnarfjordur | Outer_ARN-T | 65.8044444 | -23.9405555 | 1 | 10 | 2019 | NA | AB |
| A937T8 | Trawl | Arnarfjordur | 21 | 1 | 111.52 | 59 | Outer_Arnarfjordur | Outer_ARN-T | 65.8044444 | -23.9405555 | 1 | 10 | 2019 | NA | AB |
| A937T16 | Trawl | Arnarfjordur | 21 | 1 | 120.5 | 59 | Outer_Arnarfjordur | Outer_ARN-T | 65.8044444 | -23.9405555 | 1 | 10 | 2019 | NA | AB |
| A937T12 | Trawl | Arnarfjordur | 21.2 | 1 | 119.13 | 59 | Outer_Arnarfjordur | Outer_ARN-T | 65.8044444 | -23.9405555 | 1 | 10 | 2019 | NA | AB |
| A937T22 | Trawl | Arnarfjordur | 21.2 | 1 | 122.32 | 59 | Outer_Arnarfjordur | Outer_ARN-T | 65.8044444 | -23.9405555 | 1 | 10 | 2019 | NA | AB |
| A937T20 | Trawl | Arnarfjordur | 21.4 | 1 | 120.82 | 59 | Outer_Arnarfjordur | Outer_ARN-T | 65.8044444 | -23.9405555 | 1 | 10 | 2019 | NA | AB |
| A937T21 | Trawl | Arnarfjordur | 21.5 | 1 | 120.43 | 59 | Outer_Arnarfjordur | Outer_ARN-T | 65.8044444 | -23.9405555 | 1 | 10 | 2019 | NA | AB |
| A937T6 | Trawl | Arnarfjordur | 21.5 | 1 | 140.44 | 59 | Outer_Arnarfjordur | Outer_ARN-T | 65.8044444 | -23.9405555 | 1 | 10 | 2019 | NA | AA |
| 021178C28 | Trawl | Isafjordur | 21.5 | 1 | 140.73 | 33 | Isafjordur | ISA-T | 65.9655 | -22.45833333 | 2 | 11 | 2017 | NA | AB |
| 021178C16 | Trawl | Isafjordur | 21.9 | 1 | 137.15 | 33 | Isafjordur | ISA-T | 65.9655 | -22.45833333 | 2 | 11 | 2017 | NA | BB |
| A937T13 | Trawl | Arnarfjordur | 22.3 | 1 | 135.85 | 59 | Outer_Arnarfjordur | Outer_ARN-T | 65.8044444 | -23.9405555 | 1 | 10 | 2019 | NA | AB |
| A937T17 | Trawl | Arnarfjordur | 22.5 | 1 | 124.06 | 59 | Outer_Arnarfjordur | Outer_ARN-T | 65.8044444 | -23.9405555 | 1 | 10 | 2019 | NA | BB |
| A937T1 | Trawl | Arnarfjordur | 22.5 | 1 | 147.92 | 59 | Outer_Arnarfjordur | Outer_ARN-T | 65.8044444 | -23.9405555 | 1 | 10 | 2019 | NA | BB |
| A937T11 | Trawl | Arnarfjordur | 22.5 | 1 | 148.6 | 59 | Outer_Arnarfjordur | Outer_ARN-T | 65.8044444 | -23.9405555 | 1 | 10 | 2019 | NA | AA |
| A937T10 | Trawl | Arnarfjordur | 22.8 | 1 | 150.67 | 59 | Outer_Arnarfjordur | Outer_ARN-T | 65.8044444 | -23.9405555 | 1 | 10 | 2019 | NA | AB |
| A937T3 | Trawl | Arnarfjordur | 23.5 | 1 | 187.36 | 59 | Outer_Arnarfjordur | Outer_ARN-T | 65.8044444 | -23.9405555 | 1 | 10 | 2019 | NA | AA |
| 021178C36 | Trawl | Isafjordur | 23.9 | 1 | 160.2 | 33 | Isafjordur | ISA-T | 65.9655 | -22.45833333 | 2 | 11 | 2017 | NA | AB |
| A937T15 | Trawl | Arnarfjordur | 24 | 1 | 175.5 | 59 | Outer_Arnarfjordur | Outer_ARN-T | 65.8044444 | -23.9405555 | 1 | 10 | 2019 | NA | AA |
| E268BS12 | Seine | Strandir | 3 | 0 | 0.29 | 0 | Eyjar | EYJ-S | 65.8331 | -21.289 | 26 | 8 | 2019 | NA | AA |
| BS268BS30 | Seine | Strandir | 3.1 | 0 | 0.3 | 0 | Bassastadir | BAS-S | 65.8945 | -22.387 | 26 | 8 | 2019 | NA | AA |
| E268BS6 | Seine | Strandir | 3.1 | 0 | 0.31 | 0 | Eyjar | EYJ-S | 65.8331 | -21.289 | 26 | 8 | 2019 | NA | AA |
| E268BS50 | Seine | Strandir | 3.1 | 0 | 0.35 | 0 | Eyjar | EYJ-S | 65.8331 | -21.289 | 26 | 8 | 2019 | NA | AA |
| BS268BS16 | Seine | Strandir | 3.2 | 0 | 0.22 | 0 | Bassastadir | BAS-S | 65.8945 | -22.387 | 26 | 8 | 2019 | NA | AB |
| BS268BS63 | Seine | Strandir | 3.2 | 0 | 0.27 | 0 | Bassastadir | BAS-S | 65.8945 | -22.387 | 26 | 8 | 2019 | NA | AA |
| BS268BS34 | Seine | Strandir | 3.2 | 0 | 0.28 | 0 | Bassastadir | BAS-S | 65.8945 | -22.387 | 26 | 8 | 2019 | NA | AA |
| E268BS43 | Seine | Strandir | 3.2 | 0 | 0.32 | 0 | Eyjar | EYJ-S | 65.8331 | -21.289 | 26 | 8 | 2019 | NA | AA |
| E268BS18 | Seine | Strandir | 3.3 | 0 | 0.33 | 0 | Eyjar | EYJ-S | 65.8331 | -21.289 | 26 | 8 | 2019 | NA | AA |
| 2508BSC7 | Seine | Strandir | 3.3 | 0 | 0.36 | 0 | Bassastadir | BAS-S | 65.75949 | -23.47852 | 25 | 8 | 2017 | NA | AA |
| BS268BS31 | Seine | Strandir | 3.3 | 0 | 0.36 | 0 | Bassastadir | BAS-S | 65.8945 | -22.387 | 26 | 8 | 2019 | NA | AA |
| E268BS14 | Seine | Strandir | 3.3 | 0 | 0.36 | 0 | Eyjar | EYJ-S | 65.8331 | -21.289 | 26 | 8 | 2019 | NA | AA |
| E268BS39 | Seine | Strandir | 3.3 | 0 | 0.36 | 0 | Eyjar | EYJ-S | 65.8331 | -21.289 | 26 | 8 | 2019 | NA | AA |
| E268BS16 | Seine | Strandir | 3.3 | 0 | 0.4 | 0 | Eyjar | EYJ-S | 65.8331 | -21.289 | 26 | 8 | 2019 | NA | AA |
| BS268BS25 | Seine | Strandir | 3.4 | 0 | 0.32 | 0 | Bassastadir | BAS-S | 65.8945 | -22.387 | 26 | 8 | 2019 | NA | AA |
| E268BS7 | Seine | Strandir | 3.4 | 0 | 0.36 | 0 | Eyjar | EYJ-S | 65.8331 | -21.289 | 26 | 8 | 2019 | NA | AB |
| BS268BS20 | Seine | Strandir | 3.4 | 0 | 0.38 | 0 | Bassastadir | BAS-S | 65.8945 | -22.387 | 26 | 8 | 2019 | NA | AA |
| BS268BS49 | Seine | Strandir | 3.4 | 0 | 0.38 | 0 | Bassastadir | BAS-S | 65.8945 | -22.387 | 26 | 8 | 2019 | NA | AA |
| E268BS48 | Seine | Strandir | 3.4 | 0 | 0.39 | 0 | Eyjar | EYJ-S | 65.8331 | -21.289 | 26 | 8 | 2019 | NA | AA |
| BS268BS59 | Seine | Strandir | 3.5 | 0 | 0.4 | 0 | Bassastadir | BAS-S | 65.8945 | -22.387 | 26 | 8 | 2019 | NA | AA |
| E268BS49 | Seine | Strandir | 3.5 | 0 | 0.39 | 0 | Eyjar | EYJ-S | 65.8331 | -21.289 | 26 | 8 | 2019 | NA | AA |
| BS268BS61 | Seine | Strandir | 3.5 | 0 | 0.41 | 0 | Bassastadir | BAS-S | 65.8945 | -22.387 | 26 | 8 | 2019 | NA | AA |
| E268BS44 | Seine | Strandir | 3.5 | 0 | 0.43 | 0 | Eyjar | EYJ-S | 65.8331 | -21.289 | 26 | 8 | 2019 | NA | AA |
| E268BS2 | Seine | Strandir | 3.5 | 0 | 0.48 | 0 | Eyjar | EYJ-S | 65.8331 | -21.289 | 26 | 8 | 2019 | NA | AA |
| E268BS10 | Seine | Strandir | 3.6 | 0 | 0.4 | 0 | Eyjar | EYJ-S | 65.8331 | -21.289 | 26 | 8 | 2019 | NA | AA |
| E268BS35 | Seine | Strandir | 3.6 | 0 | 0.4 | 0 | Eyjar | EYJ-S | 65.8331 | -21.289 | 26 | 8 | 2019 | NA | AB |
| E268BS25 | Seine | Strandir | 3.6 | 0 | 0.46 | 0 | Eyjar | EYJ-S | 65.8331 | -21.289 | 26 | 8 | 2019 | NA | AA |
| E268BS40 | Seine | Strandir | 3.6 | 0 | 0.46 | 0 | Eyjar | EYJ-S | 65.8331 | -21.289 | 26 | 8 | 2019 | NA | AA |
| E268BS1 | Seine | Strandir | 3.6 | 0 | 0.5 | 0 | Eyjar | EYJ-S | 65.8331 | -21.289 | 26 | 8 | 2019 | NA | AA |
| E268BS30 | Seine | Strandir | 3.6 | 0 | 0.53 | 0 | Eyjar | EYJ-S | 65.8331 | -21.289 | 26 | 8 | 2019 | NA | AA |
| E268BS47 | Seine | Strandir | 3.7 | 0 | 0.43 | 0 | Eyjar | EYJ-S | 65.8331 | -21.289 | 26 | 8 | 2019 | NA | AA |
| E268BS51 | Seine | Strandir | 3.7 | 0 | 0.44 | 0 | Eyjar | EYJ-S | 65.8331 | -21.289 | 26 | 8 | 2019 | NA | AA |
| E268BS29 | Seine | Strandir | 3.7 | 0 | 0.51 | 0 | Eyjar | EYJ-S | 65.8331 | -21.289 | 26 | 8 | 2019 | NA | AA |
| E268BS37 | Seine | Strandir | 3.7 | 0 | 0.54 | 0 | Eyjar | EYJ-S | 65.8331 | -21.289 | 26 | 8 | 2019 | NA | AA |
| BS268BS50 | Seine | Strandir | 3.7 | 0 | 0.56 | 0 | Bassastadir | BAS-S | 65.8945 | -22.387 | 26 | 8 | 2019 | NA | AB |
| E268BS27 | Seine | Strandir | 3.8 | 0 | 0.49 | 0 | Eyjar | EYJ-S | 65.8331 | -21.289 | 26 | 8 | 2019 | NA | AA |
| BS268BS29 | Seine | Strandir | 3.8 | 0 | 0.5 | 0 | Bassastadir | BAS-S | 65.8945 | -22.387 | 26 | 8 | 2019 | NA | AA |
| BS268BS57 | Seine | Strandir | 3.8 | 0 | 0.5 | 0 | Bassastadir | BAS-S | 65.8945 | -22.387 | 26 | 8 | 2019 | NA | AA |
| 2608EYC2 | Seine | Strandir | 3.8 | 0 | 0.53 | 0 | Eyjar | EYJ-S | 65.98089 | -23.03125 | 26 | 8 | 2017 | NA | AA |
| E268BS36 | Seine | Strandir | 3.8 | 0 | 0.54 | 0 | Eyjar | EYJ-S | 65.8331 | -21.289 | 26 | 8 | 2019 | NA | AA |
| E268BS17 | Seine | Strandir | 3.8 | 0 | 0.56 | 0 | Eyjar | EYJ-S | 65.8331 | -21.289 | 26 | 8 | 2019 | NA | AA |
| E268BS20 | Seine | Strandir | 3.8 | 0 | 0.58 | 0 | Eyjar | EYJ-S | 65.8331 | -21.289 | 26 | 8 | 2019 | NA | AA |
| BS268BS37 | Seine | Strandir | 3.8 | 0 | 0.59 | 0 | Bassastadir | BAS-S | 65.8945 | -22.387 | 26 | 8 | 2019 | NA | AA |
| 2808SSC5 | Seine | Isafjordur | 3.9 | 0 | 0.43 | 0 | Arngerdareyri | ARG-S | 65.96486 | -22.89687 | 28 | 8 | 2017 | NA | AA |
| BS268BS52 | Seine | Strandir | 3.9 | 0 | 0.45 | 0 | Bassastadir | BAS-S | 65.8945 | -22.387 | 26 | 8 | 2019 | NA | AB |
| BS268BS17 | Seine | Strandir | 3.9 | 0 | 0.53 | 0 | Bassastadir | BAS-S | 65.8945 | -22.387 | 26 | 8 | 2019 | NA | AA |
| BS268BS60 | Seine | Strandir | 3.9 | 0 | 0.56 | 0 | Bassastadir | BAS-S | 65.8945 | -22.387 | 26 | 8 | 2019 | NA | AA |
| BS268BS54 | Seine | Strandir | 3.9 | 0 | 0.56 | 0 | Bassastadir | BAS-S | 65.8945 | -22.387 | 26 | 8 | 2019 | NA | AB |
| E268BS26 | Seine | Strandir | 3.9 | 0 | 0.68 | 0 | Eyjar | EYJ-S | 65.8331 | -21.289 | 26 | 8 | 2019 | NA | AA |
| BS268BS45 | Seine | Strandir | 4 | 0 | 0.61 | 0 | Bassastadir | BAS-S | 65.8945 | -22.387 | 26 | 8 | 2019 | NA | AA |
| E268BS38 | Seine | Strandir | 4 | 0 | 0.64 | 0 | Eyjar | EYJ-S | 65.8331 | -21.289 | 26 | 8 | 2019 | NA | AA |
| E268BS11 | Seine | Strandir | 4 | 0 | 0.68 | 0 | Eyjar | EYJ-S | 65.8331 | -21.289 | 26 | 8 | 2019 | NA | AA |
| BS268BS46 | Seine | Strandir | 4 | 0 | 0.69 | 0 | Bassastadir | BAS-S | 65.8945 | -22.387 | 26 | 8 | 2019 | NA | AA |
| 2508BSC8 | Seine | Strandir | 4 | 0 | 1.08 | 0 | Bassastadir | BAS-S | 65.75949 | -23.47852 | 25 | 8 | 2017 | NA | AB |
| BS268BS35 | Seine | Strandir | 4.1 | 0 | 0.59 | 0 | Bassastadir | BAS-S | 65.8945 | -22.387 | 26 | 8 | 2019 | NA | AA |
| E268BS8 | Seine | Strandir | 4.1 | 0 | 0.62 | 0 | Eyjar | EYJ-S | 65.8331 | -21.289 | 26 | 8 | 2019 | NA | AA |
| E268BS9 | Seine | Strandir | 4.1 | 0 | 0.63 | 0 | Eyjar | EYJ-S | 65.8331 | -21.289 | 26 | 8 | 2019 | NA | AA |
| BS268BS64 | Seine | Strandir | 4.1 | 0 | 0.66 | 0 | Bassastadir | BAS-S | 65.8945 | -22.387 | 26 | 8 | 2019 | NA | AA |
| E268BS52 | Seine | Strandir | 4.1 | 0 | 0.68 | 0 | Eyjar | EYJ-S | 65.8331 | -21.289 | 26 | 8 | 2019 | NA | AA |
| E268BS13 | Seine | Strandir | 4.1 | 0 | 0.71 | 0 | Eyjar | EYJ-S | 65.8331 | -21.289 | 26 | 8 | 2019 | NA | AA |
| BS268BS19 | Seine | Strandir | 4.1 | 0 | 0.79 | 0 | Bassastadir | BAS-S | 65.8945 | -22.387 | 26 | 8 | 2019 | NA | AA |
| 2808IFC5 | Seine | Isafjordur | 4.2 | 0 | 0.6 | 0 | Arngerdareyri | ARG-S | 65.96486 | -22.89687 | 28 | 8 | 2017 | NA | AA |
| 2808SSC13 | Seine | Isafjordur | 4.2 | 0 | 0.64 | 0 | Arngerdareyri | ARG-S | 65.96486 | -22.89687 | 28 | 8 | 2017 | NA | AA |
| E268BS5 | Seine | Strandir | 4.2 | 0 | 0.68 | 0 | Eyjar | EYJ-S | 65.8331 | -21.289 | 26 | 8 | 2019 | NA | AA |
| E268BS21 | Seine | Strandir | 4.2 | 0 | 0.73 | 0 | Eyjar | EYJ-S | 65.8331 | -21.289 | 26 | 8 | 2019 | NA | AA |
| 2808SSC7 | Seine | Isafjordur | 4.2 | 0 | 0.98 | 0 | Arngerdareyri | ARG-S | 65.96486 | -22.89687 | 28 | 8 | 2017 | NA | AB |
| 2508BSC25 | Seine | Strandir | 4.2 | 0 | NA | 0 | Bassastadir | BAS-S | 65.75949 | -23.47852 | 25 | 8 | 2017 | NA | AA |
| 2808SSC3 | Seine | Isafjordur | 4.3 | 0 | 0.57 | 0 | Arngerdareyri | ARG-S | 65.96486 | -22.89687 | 28 | 8 | 2017 | NA | AA |
| 2808SSC12 | Seine | Isafjordur | 4.3 | 0 | 0.6 | 0 | Arngerdareyri | ARG-S | 65.96486 | -22.89687 | 28 | 8 | 2017 | NA | AB |
| 2508BSC2 | Seine | Strandir | 4.3 | 0 | 0.75 | 0 | Bassastadir | BAS-S | 65.75949 | -23.47852 | 25 | 8 | 2017 | NA | AA |
| 2608EYC8 | Seine | Strandir | 4.4 | 0 | 0.62 | 0 | Eyjar | EYJ-S | 65.98089 | -23.03125 | 26 | 8 | 2017 | NA | AB |
| BS268BS47 | Seine | Strandir | 4.4 | 0 | 0.7 | 0 | Bassastadir | BAS-S | 65.8945 | -22.387 | 26 | 8 | 2019 | NA | AA |
| 2808SSC11 | Seine | Isafjordur | 4.4 | 0 | 0.73 | 0 | Arngerdareyri | ARG-S | 65.96486 | -22.89687 | 28 | 8 | 2017 | NA | AA |
| E268BS15 | Seine | Strandir | 4.4 | 0 | 0.8 | 0 | Eyjar | EYJ-S | 65.8331 | -21.289 | 26 | 8 | 2019 | NA | AA |
| E268BS23 | Seine | Strandir | 4.4 | 0 | 0.9 | 0 | Eyjar | EYJ-S | 65.8331 | -21.289 | 26 | 8 | 2019 | NA | AA |
| 2808IFC37 | Seine | Isafjordur | 4.5 | 0 | 0.7 | 0 | Arngerdareyri | ARG-S | 65.96486 | -22.89687 | 28 | 8 | 2017 | NA | AA |
| 2508BSC24 | Seine | Strandir | 4.5 | 0 | 0.71 | 0 | Bassastadir | BAS-S | 65.75949 | -23.47852 | 25 | 8 | 2017 | NA | AA |
| E268BS22 | Seine | Strandir | 4.5 | 0 | 0.81 | 0 | Eyjar | EYJ-S | 65.8331 | -21.289 | 26 | 8 | 2019 | NA | AA |
| 2508BSC10 | Seine | Strandir | 4.5 | 0 | 0.81 | 0 | Bassastadir | BAS-S | 65.75949 | -23.47852 | 25 | 8 | 2017 | NA | BB |
| 2808IFC33 | Seine | Isafjordur | 4.5 | 0 | 0.9 | 0 | Arngerdareyri | ARG-S | 65.96486 | -22.89687 | 28 | 8 | 2017 | NA | AA |
| 2808IFC1 | Seine | Isafjordur | 4.5 | 0 | 1.36 | 0 | Arngerdareyri | ARG-S | 65.96486 | -22.89687 | 28 | 8 | 2017 | NA | AA |
| 2808IFC28 | Seine | Isafjordur | 4.6 | 0 | 0.74 | 0 | Arngerdareyri | ARG-S | 65.96486 | -22.89687 | 28 | 8 | 2017 | NA | AA |
| E268BS19 | Seine | Strandir | 4.6 | 0 | 0.89 | 0 | Eyjar | EYJ-S | 65.8331 | -21.289 | 26 | 8 | 2019 | NA | AA |
| 2608EYC9 | Seine | Strandir | 4.6 | 0 | 0.93 | 0 | Eyjar | EYJ-S | 65.98089 | -23.03125 | 26 | 8 | 2017 | NA | AB |
| 2808IFC24 | Seine | Isafjordur | 4.6 | 0 | 0.96 | 0 | Arngerdareyri | ARG-S | 65.96486 | -22.89687 | 28 | 8 | 2017 | NA | AA |
| 2508BSC12 | Seine | Strandir | 4.6 | 0 | 0.98 | 0 | Bassastadir | BAS-S | 65.75949 | -23.47852 | 25 | 8 | 2017 | NA | AA |
| 2808SSC10 | Seine | Isafjordur | 4.7 | 0 | 0.9 | 0 | Arngerdareyri | ARG-S | 65.96486 | -22.89687 | 28 | 8 | 2017 | NA | AA |
| 2808IFC39 | Seine | Isafjordur | 4.7 | 0 | 0.96 | 0 | Arngerdareyri | ARG-S | 65.96486 | -22.89687 | 28 | 8 | 2017 | NA | AA |
| BS268BS1 | Seine | Strandir | 4.7 | 0 | 1.03 | 0 | Bassastadir | BAS-S | 65.8945 | -22.387 | 26 | 8 | 2019 | NA | AA |
| 0509AKC15 | Seine | Arnarfjordur | 4.7 | 0 | 1.18 | 0 | Audkula | AUD-S | 65.96486 | -22.89687 | 5 | 9 | 2017 | NA | AA |
| 2508BSC22 | Seine | Strandir | 4.8 | 0 | 0.98 | 0 | Bassastadir | BAS-S | 65.75949 | -23.47852 | 25 | 8 | 2017 | NA | AA |
| 0509AKC16 | Seine | Arnarfjordur | 4.8 | 0 | 1.04 | 0 | Audkula | AUD-S | 65.96486 | -22.89687 | 5 | 9 | 2017 | NA | AA |
| 2508BSC18 | Seine | Strandir | 4.8 | 0 | 1.06 | 0 | Bassastadir | BAS-S | 65.75949 | -23.47852 | 25 | 8 | 2017 | NA | AA |
| E268BS24 | Seine | Strandir | 4.8 | 0 | 1.09 | 0 | Eyjar | EYJ-S | 65.8331 | -21.289 | 26 | 8 | 2019 | NA | AA |
| 0509AKC14 | Seine | Arnarfjordur | 4.8 | 0 | 1.13 | 0 | Audkula | AUD-S | 65.96486 | -22.89687 | 5 | 9 | 2017 | NA | AA |
| 2508BSC14 | Seine | Strandir | 4.9 | 0 | 0.92 | 0 | Bassastadir | BAS-S | 65.75949 | -23.47852 | 25 | 8 | 2017 | NA | AB |
| 2508BSC15 | Seine | Strandir | 4.9 | 0 | 0.95 | 0 | Bassastadir | BAS-S | 65.75949 | -23.47852 | 25 | 8 | 2017 | NA | AA |
| 0509AKC10 | Seine | Arnarfjordur | 4.9 | 0 | 1.06 | 0 | Audkula | AUD-S | 65.96486 | -22.89687 | 5 | 9 | 2017 | NA | AA |
| 0509AKC9 | Seine | Arnarfjordur | 4.9 | 0 | 1.07 | 0 | Audkula | AUD-S | 65.96486 | -22.89687 | 5 | 9 | 2017 | NA | AA |
| 0509AKC22 | Seine | Arnarfjordur | 4.9 | 0 | 1.12 | 0 | Audkula | AUD-S | 65.96486 | -22.89687 | 5 | 9 | 2017 | NA | AA |
| 2608EYC5 | Seine | Strandir | 4.9 | 0 | 1.14 | 0 | Eyjar | EYJ-S | 65.98089 | -23.03125 | 26 | 8 | 2017 | NA | AB |
| E268BS4 | Seine | Strandir | 4.9 | 0 | 1.17 | 0 | Eyjar | EYJ-S | 65.8331 | -21.289 | 26 | 8 | 2019 | NA | AB |
| 2210IFC46 | Seine | Isafjordur | 4.9 | 0 | 1.26 | 0 | Arngerdareyri | ARG-S | 65.89512 | -22.38229 | 22 | 10 | 2017 | NA | AB |
| 2708TFC5 | Seine | Breidafjordur | 4.9 | 0 | 1.29 | 0 | Thorskafjordur | TOR-S | 65.584040 | -22.131605 | 5 | 9 | 2017 | NA | AA |
| 2508BSC21 | Seine | Strandir | 5 | 0 | 1.12 | 0 | Bassastadir | BAS-S | 65.75949 | -23.47852 | 25 | 8 | 2017 | NA | AA |
| 0509AKC11 | Seine | Arnarfjordur | 5 | 0 | 1.17 | 0 | Audkula | AUD-S | 65.96486 | -22.89687 | 5 | 9 | 2017 | NA | AA |
| 0509AKC3 | Seine | Arnarfjordur | 5 | 0 | 1.22 | 0 | Audkula | AUD-S | 65.96486 | -22.89687 | 5 | 9 | 2017 | NA | AA |
| 2708TFC4 | Seine | Breidafjordur | 5 | 0 | 1.22 | 0 | Thorskafjordur | TOR-S | 65.584040 | -22.131605 | 5 | 9 | 2017 | NA | AA |
| 0509AKC21 | Seine | Arnarfjordur | 5 | 0 | 1.25 | 0 | Audkula | AUD-S | 65.96486 | -22.89687 | 5 | 9 | 2017 | NA | AA |
| 2508BSC20 | Seine | Strandir | 5 | 0 | 1.25 | 0 | Bassastadir | BAS-S | 65.75949 | -23.47852 | 25 | 8 | 2017 | NA | AA |
| 2210IFC49 | Seine | Isafjordur | 5 | 0 | 1.25 | 0 | Arngerdareyri | ARG-S | 65.89512 | -22.38229 | 22 | 10 | 2017 | NA | AB |
| 2608EYC10 | Seine | Strandir | 5 | 0 | 1.26 | 0 | Eyjar | EYJ-S | 65.98089 | -23.03125 | 26 | 8 | 2017 | NA | AB |
| BS268BS56 | Seine | Strandir | 5 | 0 | 1.31 | 0 | Bassastadir | BAS-S | 65.8945 | -22.387 | 26 | 8 | 2019 | NA | AA |
| 1610AKC15 | Seine | Arnarfjordur | 5 | 0 | 1.33 | 0 | Audkula | AUD-S | 65.83339 | -21.28872 | 16 | 10 | 2017 | NA | AA |
| 2708KSc6 | Seine | Breidafjordur | 5 | 0 | 1.42 | 0 | Thorskafjordur | TOR-S | 65.584040 | -22.131605 | 5 | 9 | 2017 | NA | AB |
| 2808SSC2 | Seine | Isafjordur | 5.1 | 0 | 1.2 | 0 | Arngerdareyri | ARG-S | 65.96486 | -22.89687 | 28 | 8 | 2017 | NA | AA |
| 2808IFC27 | Seine | Isafjordur | 5.1 | 0 | 1.23 | 0 | Arngerdareyri | ARG-S | 65.96486 | -22.89687 | 28 | 8 | 2017 | NA | AA |
| 2808IFC4 | Seine | Isafjordur | 5.1 | 0 | 1.35 | 0 | Arngerdareyri | ARG-S | 65.96486 | -22.89687 | 28 | 8 | 2017 | NA | AA |
| 2210IFC39 | Seine | Isafjordur | 5.1 | 0 | 1.38 | 0 | Arngerdareyri | ARG-S | 65.89512 | -22.38229 | 22 | 10 | 2017 | NA | AB |
| 2608EYC1 | Seine | Strandir | 5.1 | 0 | 1.7 | 0 | Eyjar | EYJ-S | 65.98089 | -23.03125 | 26 | 8 | 2017 | NA | BB |
| 1610AKC5 | Seine | Arnarfjordur | 5.2 | 0 | 1.09 | 0 | Audkula | AUD-S | 65.83339 | -21.28872 | 16 | 10 | 2017 | NA | AA |
| 2808IFC13 | Seine | Isafjordur | 5.2 | 0 | 1.19 | 0 | Arngerdareyri | ARG-S | 65.96486 | -22.89687 | 28 | 8 | 2017 | NA | AA |
| 0509AKC23 | Seine | Arnarfjordur | 5.2 | 0 | 1.25 | 0 | Audkula | AUD-S | 65.96486 | -22.89687 | 5 | 9 | 2017 | NA | AA |
| 2508BSC16 | Seine | Strandir | 5.2 | 0 | 1.28 | 0 | Bassastadir | BAS-S | 65.75949 | -23.47852 | 25 | 8 | 2017 | NA | AB |
| 2508BSC6 | Seine | Strandir | 5.2 | 0 | 1.41 | 0 | Bassastadir | BAS-S | 65.75949 | -23.47852 | 25 | 8 | 2017 | NA | AA |
| 2210IFC2 | Seine | Isafjordur | 5.2 | 0 | 1.44 | 0 | Arngerdareyri | ARG-S | 65.89512 | -22.38229 | 22 | 10 | 2017 | NA | AB |
| 2110BSC2 | Seine | Strandir | 5.3 | 0 | 0.99 | 0 | Bassastadir | BAS-S | 65.99392 | -22.94096 | 21 | 10 | 2017 | NA | AA |
| 2508BSC5 | Seine | Strandir | 5.3 | 0 | 1.29 | 0 | Bassastadir | BAS-S | 65.75949 | -23.47852 | 25 | 8 | 2017 | NA | AA |
| 0509AKC8 | Seine | Arnarfjordur | 5.3 | 0 | 1.32 | 0 | Audkula | AUD-S | 65.96486 | -22.89687 | 5 | 9 | 2017 | NA | AA |
| 2708TFC2 | Seine | Breidafjordur | 5.3 | 0 | 1.32 | 0 | Thorskafjordur | TOR-S | 65.584040 | -22.131605 | 5 | 9 | 2017 | NA | AA |
| 2808SSC9 | Seine | Isafjordur | 5.3 | 0 | 1.34 | 0 | Arngerdareyri | ARG-S | 65.96486 | -22.89687 | 28 | 8 | 2017 | NA | AA |
| 1610AKC1 | Seine | Arnarfjordur | 5.3 | 0 | 1.38 | 0 | Audkula | AUD-S | 65.83339 | -21.28872 | 16 | 10 | 2017 | NA | AA |
| 2508BSC4 | Seine | Strandir | 5.3 | 0 | 1.4 | 0 | Bassastadir | BAS-S | 65.75949 | -23.47852 | 25 | 8 | 2017 | NA | AA |
| 2508BSC17 | Seine | Strandir | 5.3 | 0 | 1.4 | 0 | Bassastadir | BAS-S | 65.75949 | -23.47852 | 25 | 8 | 2017 | NA | AA |
| 2210IFC42 | Seine | Isafjordur | 5.3 | 0 | 1.4 | 0 | Arngerdareyri | ARG-S | 65.89512 | -22.38229 | 22 | 10 | 2017 | NA | AB |
| 2508BSC1 | Seine | Strandir | 5.3 | 0 | 1.41 | 0 | Bassastadir | BAS-S | 65.75949 | -23.47852 | 25 | 8 | 2017 | NA | AA |
| 2210IFC48 | Seine | Isafjordur | 5.3 | 0 | 1.47 | 0 | Arngerdareyri | ARG-S | 65.89512 | -22.38229 | 22 | 10 | 2017 | NA | AB |
| 2808IFC7 | Seine | Isafjordur | 5.3 | 0 | 1.61 | 0 | Arngerdareyri | ARG-S | 65.96486 | -22.89687 | 28 | 8 | 2017 | NA | AB |
| 2508BSC27 | Seine | Strandir | 5.3 | 0 | 5.3 | 0 | Bassastadir | BAS-S | 65.75949 | -23.47852 | 25 | 8 | 2017 | NA | AA |
| 0509AKC20 | Seine | Arnarfjordur | 5.4 | 0 | 1.36 | 0 | Audkula | AUD-S | 65.96486 | -22.89687 | 5 | 9 | 2017 | NA | AA |
| 0509AKC19 | Seine | Arnarfjordur | 5.4 | 0 | 1.4 | 0 | Audkula | AUD-S | 65.96486 | -22.89687 | 5 | 9 | 2017 | NA | AA |
| 2808IFC19 | Seine | Isafjordur | 5.4 | 0 | 1.41 | 0 | Arngerdareyri | ARG-S | 65.96486 | -22.89687 | 28 | 8 | 2017 | NA | AA |
| 2508BSC19 | Seine | Strandir | 5.4 | 0 | 1.5 | 0 | Bassastadir | BAS-S | 65.75949 | -23.47852 | 25 | 8 | 2017 | NA | AA |
| 0509AKC7 | Seine | Arnarfjordur | 5.4 | 0 | 1.6 | 0 | Audkula | AUD-S | 65.96486 | -22.89687 | 5 | 9 | 2017 | NA | AA |
| 2808SSC14 | Seine | Isafjordur | 5.4 | 0 | 1.61 | 0 | Arngerdareyri | ARG-S | 65.96486 | -22.89687 | 28 | 8 | 2017 | NA | AA |
| 2708KSc2 | Seine | Breidafjordur | 5.4 | 0 | 1.7 | 0 | Thorskafjordur | TOR-S | 65.584040 | -22.131605 | 5 | 9 | 2017 | NA | AB |
| 1610AKC2 | Seine | Arnarfjordur | 5.5 | 0 | 1.35 | 0 | Audkula | AUD-S | 65.83339 | -21.28872 | 16 | 10 | 2017 | NA | AA |
| 2210IFC5 | Seine | Isafjordur | 5.5 | 0 | 1.36 | 0 | Arngerdareyri | ARG-S | 65.89512 | -22.38229 | 22 | 10 | 2017 | NA | AB |
| 2808IFC30 | Seine | Isafjordur | 5.5 | 0 | 1.37 | 0 | Arngerdareyri | ARG-S | 65.96486 | -22.89687 | 28 | 8 | 2017 | NA | AA |
| 2210IFC40 | Seine | Isafjordur | 5.5 | 0 | 1.43 | 0 | Arngerdareyri | ARG-S | 65.89512 | -22.38229 | 22 | 10 | 2017 | NA | AA |
| 2210IFC41 | Seine | Isafjordur | 5.5 | 0 | 1.43 | 0 | Arngerdareyri | ARG-S | 65.89512 | -22.38229 | 22 | 10 | 2017 | NA | AB |
| 2210IFC33 | Seine | Isafjordur | 5.5 | 0 | 1.43 | 0 | Arngerdareyri | ARG-S | 65.89512 | -22.38229 | 22 | 10 | 2017 | NA | BB |
| 2210IFC17 | Seine | Isafjordur | 5.5 | 0 | 1.46 | 0 | Arngerdareyri | ARG-S | 65.89512 | -22.38229 | 22 | 10 | 2017 | NA | AA |
| 0509AKC13 | Seine | Arnarfjordur | 5.5 | 0 | 1.54 | 0 | Audkula | AUD-S | 65.96486 | -22.89687 | 5 | 9 | 2017 | NA | AA |
| 2508BSC26 | Seine | Strandir | 5.5 | 0 | 1.55 | 0 | Bassastadir | BAS-S | 65.75949 | -23.47852 | 25 | 8 | 2017 | NA | AA |
| 1610AKC8 | Seine | Arnarfjordur | 5.5 | 0 | 1.59 | 0 | Audkula | AUD-S | 65.83339 | -21.28872 | 16 | 10 | 2017 | NA | AA |
| 2508BSC9 | Seine | Strandir | 5.5 | 0 | 1.59 | 0 | Bassastadir | BAS-S | 65.75949 | -23.47852 | 25 | 8 | 2017 | NA | AA |
| 2608EYC3 | Seine | Strandir | 5.5 | 0 | 1.63 | 0 | Eyjar | EYJ-S | 65.98089 | -23.03125 | 26 | 8 | 2017 | NA | AA |
| 2608EYC6 | Seine | Strandir | 5.5 | 0 | 1.64 | 0 | Eyjar | EYJ-S | 65.98089 | -23.03125 | 26 | 8 | 2017 | NA | AB |
| AK109BS33 | Seine | Arnarfjordur | 5.5 | 0 | 1.66 | 0 | Audkula | AUD-S | 65.96486 | -22.89687 | 10 | 9 | 2019 | NA | AB |
| 2708?FC2 | Seine | Breidafjordur | 5.5 | 0 | 1.67 | 0 | Thorskafjordur | TOR-S | 65.584040 | -22.131605 | 5 | 9 | 2017 | NA | AA |
| 2708?FC3 | Seine | Breidafjordur | 5.5 | 0 | 1.67 | 0 | Thorskafjordur | TOR-S | 65.584040 | -22.131605 | 5 | 9 | 2017 | NA | AA |
| 2210IFC14 | Seine | Isafjordur | 5.5 | 0 | 1.72 | 0 | Arngerdareyri | ARG-S | 65.89512 | -22.38229 | 22 | 10 | 2017 | NA | AB |
| 1610AKC14 | Seine | Arnarfjordur | 5.5 | 0 | 1.76 | 0 | Audkula | AUD-S | 65.83339 | -21.28872 | 16 | 10 | 2017 | NA | AA |
| 1610AKC18 | Seine | Arnarfjordur | 5.5 | 0 | 1.76 | 0 | Audkula | AUD-S | 65.83339 | -21.28872 | 16 | 10 | 2017 | NA | AA |
| 2210IFC35 | Seine | Isafjordur | 5.5 | 0 | 1.78 | 0 | Arngerdareyri | ARG-S | 65.89512 | -22.38229 | 22 | 10 | 2017 | NA | AB |
| 2608EYC4 | Seine | Strandir | 5.5 | 0 | 1.79 | 0 | Eyjar | EYJ-S | 65.98089 | -23.03125 | 26 | 8 | 2017 | NA | AB |
| 2708TFC6 | Seine | Breidafjordur | 5.5 | 0 | 1.82 | 0 | Thorskafjordur | TOR-S | 65.584040 | -22.131605 | 5 | 9 | 2017 | NA | AA |
| 2808IFC12 | Seine | Isafjordur | 5.5 | 0 | 1.83 | 0 | Arngerdareyri | ARG-S | 65.96486 | -22.89687 | 28 | 8 | 2017 | NA | AA |
| 0509AKC6 | Seine | Arnarfjordur | 5.6 | 0 | 1.54 | 0 | Audkula | AUD-S | 65.96486 | -22.89687 | 5 | 9 | 2017 | NA | AA |
| 1610AKC17 | Seine | Arnarfjordur | 5.6 | 0 | 1.54 | 0 | Audkula | AUD-S | 65.83339 | -21.28872 | 16 | 10 | 2017 | NA | AA |
| 2808SSC1 | Seine | Isafjordur | 5.6 | 0 | 1.55 | 0 | Arngerdareyri | ARG-S | 65.96486 | -22.89687 | 28 | 8 | 2017 | NA | AB |
| 2508BSC13 | Seine | Strandir | 5.6 | 0 | 1.56 | 0 | Bassastadir | BAS-S | 65.75949 | -23.47852 | 25 | 8 | 2017 | NA | AA |
| 1610AKC10 | Seine | Arnarfjordur | 5.6 | 0 | 1.58 | 0 | Audkula | AUD-S | 65.83339 | -21.28872 | 16 | 10 | 2017 | NA | AB |
| 2210IFC3 | Seine | Isafjordur | 5.6 | 0 | 1.6 | 0 | Arngerdareyri | ARG-S | 65.89512 | -22.38229 | 22 | 10 | 2017 | NA | AB |
| 0509AKC5 | Seine | Arnarfjordur | 5.6 | 0 | 1.65 | 0 | Audkula | AUD-S | 65.96486 | -22.89687 | 5 | 9 | 2017 | NA | AA |
| 2708?FC6 | Seine | Breidafjordur | 5.6 | 0 | 1.67 | 0 | Thorskafjordur | TOR-S | 65.584040 | -22.131605 | 5 | 9 | 2017 | NA | AA |
| 2210IFC36 | Seine | Isafjordur | 5.6 | 0 | 1.76 | 0 | Arngerdareyri | ARG-S | 65.89512 | -22.38229 | 22 | 10 | 2017 | NA | BB |
| 1610AKC16 | Seine | Arnarfjordur | 5.6 | 0 | 1.77 | 0 | Audkula | AUD-S | 65.83339 | -21.28872 | 16 | 10 | 2017 | NA | AB |
| 2708?FC10 | Seine | Breidafjordur | 5.6 | 0 | 1.78 | 0 | Thorskafjordur | TOR-S | 65.584040 | -22.131605 | 5 | 9 | 2017 | NA | AA |
| 2708KSc5 | Seine | Breidafjordur | 5.6 | 0 | 1.8 | 0 | Thorskafjordur | TOR-S | 65.584040 | -22.131605 | 5 | 9 | 2017 | NA | BB |
| 1610AKC12 | Seine | Arnarfjordur | 5.6 | 0 | 1.85 | 0 | Audkula | AUD-S | 65.83339 | -21.28872 | 16 | 10 | 2017 | NA | AA |
| 2210BSC7 | Seine | Strandir | 5.6 | 0 | 2.02 | 0 | Bassastadir | BAS-S | 66.04037 | -22.68079 | 22 | 10 | 2017 | NA | AB |
| 1610AKC13 | Seine | Arnarfjordur | 5.6 | 0 | 2.25 | 0 | Audkula | AUD-S | 65.83339 | -21.28872 | 16 | 10 | 2017 | NA | AA |
| 1610AKC3 | Seine | Arnarfjordur | 5.7 | 0 | 1.5 | 0 | Audkula | AUD-S | 65.83339 | -21.28872 | 16 | 10 | 2017 | NA | AA |
| 1610AKC6 | Seine | Arnarfjordur | 5.7 | 0 | 1.69 | 0 | Audkula | AUD-S | 65.83339 | -21.28872 | 16 | 10 | 2017 | NA | AA |
| 2210IFC7 | Seine | Isafjordur | 5.7 | 0 | 1.74 | 0 | Arngerdareyri | ARG-S | 65.89512 | -22.38229 | 22 | 10 | 2017 | NA | AA |
| 0711102C15 | Trawl | Isafjordur | 5.7 | 0 | 1.74 | 44 | Isafjordur | ISA-T | 65.90367 | -22.4085 | 7 | 11 | 2017 | NA | AB |
| 2808SSC4 | Seine | Isafjordur | 5.8 | 0 | 0.87 | 0 | Arngerdareyri | ARG-S | 65.96486 | -22.89687 | 28 | 8 | 2017 | NA | AA |
| 2210IFC8 | Seine | Isafjordur | 5.8 | 0 | 1.63 | 0 | Arngerdareyri | ARG-S | 65.89512 | -22.38229 | 22 | 10 | 2017 | NA | AA |
| 2210IFC38 | Seine | Isafjordur | 5.8 | 0 | 1.76 | 0 | Arngerdareyri | ARG-S | 65.89512 | -22.38229 | 22 | 10 | 2017 | NA | AB |
| 2210IFC43 | Seine | Isafjordur | 5.8 | 0 | 1.79 | 0 | Arngerdareyri | ARG-S | 65.89512 | -22.38229 | 22 | 10 | 2017 | NA | AA |
| 2210IFC15 | Seine | Isafjordur | 5.8 | 0 | 1.94 | 0 | Arngerdareyri | ARG-S | 65.89512 | -22.38229 | 22 | 10 | 2017 | NA | AB |
| 1610AKC7 | Seine | Arnarfjordur | 5.8 | 0 | 2.21 | 0 | Audkula | AUD-S | 65.83339 | -21.28872 | 16 | 10 | 2017 | NA | AA |
| 2210BSC14 | Seine | Strandir | 5.8 | 0 | NA | 0 | Bassastadir | BAS-S | 66.04037 | -22.68079 | 22 | 10 | 2017 | NA | AB |
| 1610AKC11 | Seine | Arnarfjordur | 5.9 | 0 | 1.86 | 0 | Audkula | AUD-S | 65.83339 | -21.28872 | 16 | 10 | 2017 | NA | AA |
| 0711102C9 | Trawl | Isafjordur | 5.9 | 0 | 1.89 | 44 | Isafjordur | ISA-T | 65.90367 | -22.4085 | 7 | 11 | 2017 | NA | AB |
| 2708KSc4 | Seine | Breidafjordur | 5.9 | 0 | 1.9 | 0 | Thorskafjordur | TOR-S | 65.584040 | -22.131605 | 5 | 9 | 2017 | NA | BB |
| 2708TFC3 | Seine | Breidafjordur | 5.9 | 0 | 1.97 | 0 | Thorskafjordur | TOR-S | 65.584040 | -22.131605 | 5 | 9 | 2017 | NA | AA |
| 1610AKC9 | Seine | Arnarfjordur | 5.9 | 0 | 2.09 | 0 | Audkula | AUD-S | 65.83339 | -21.28872 | 16 | 10 | 2017 | NA | AA |
| 2708?FC5 | Seine | Breidafjordur | 5.9 | 0 | 2.11 | 0 | Thorskafjordur | TOR-S | 65.584040 | -22.131605 | 5 | 9 | 2017 | NA | AA |
| 0509AKC2 | Seine | Arnarfjordur | 5.9 | 0 | 2.22 | 0 | Audkula | AUD-S | 65.96486 | -22.89687 | 5 | 9 | 2017 | NA | AA |
| 2210IFC13 | Seine | Isafjordur | 6 | 0 | 1.57 | 0 | Arngerdareyri | ARG-S | 65.89512 | -22.38229 | 22 | 10 | 2017 | NA | AB |
| 2210BSC5 | Seine | Strandir | 6 | 0 | 1.66 | 0 | Bassastadir | BAS-S | 66.04037 | -22.68079 | 22 | 10 | 2017 | NA | AB |
| 2608EYC7 | Seine | Strandir | 6 | 0 | 1.86 | 0 | Eyjar | EYJ-S | 65.98089 | -23.03125 | 26 | 8 | 2017 | NA | AB |
| 2210IFC1 | Seine | Isafjordur | 6 | 0 | 1.9 | 0 | Arngerdareyri | ARG-S | 65.89512 | -22.38229 | 22 | 10 | 2017 | NA | AB |
| 2110BSC3 | Seine | Strandir | 6 | 0 | 2.05 | 0 | Bassastadir | BAS-S | 65.99392 | -22.94096 | 21 | 10 | 2017 | NA | AB |
| 021177C29 | Trawl | Isafjordur | 6 | 0 | 2.1 | 43 | Mjoifjordur | MJO-T | 65.97533 | -22.55733 | 2 | 11 | 2017 | NA | AB |
| 0509AKC18 | Seine | Arnarfjordur | 6 | 0 | 2.3 | 0 | Audkula | AUD-S | 65.96486 | -22.89687 | 5 | 9 | 2017 | NA | AA |
| 0509AKC1 | Seine | Arnarfjordur | 6 | 0 | 2.41 | 0 | Audkula | AUD-S | 65.96486 | -22.89687 | 5 | 9 | 2017 | NA | AB |
| 2708?FC7 | Seine | Breidafjordur | 6 | 0 | NA | 0 | Thorskafjordur | TOR-S | 65.584040 | -22.131605 | 5 | 9 | 2017 | NA | AA |
| 2708?FC20 | Seine | Breidafjordur | 6 | 0 | NA | 0 | Thorskafjordur | TOR-S | 65.584040 | -22.131605 | 5 | 9 | 2017 | NA | AB |
| 011167C28 | Trawl | Isafjordur | 6.1 | 0 | 2.04 | 48 | Isafjordur | ISA-T | 65.92233 | -22.4065 | 1 | 11 | 2017 | NA | AB |
| 2708KSc8 | Seine | Breidafjordur | 6.1 | 0 | 2.04 | 0 | Thorskafjordur | TOR-S | 65.584040 | -22.131605 | 5 | 9 | 2017 | NA | AB |
| 0711102C8 | Trawl | Isafjordur | 6.1 | 0 | 2.06 | 44 | Isafjordur | ISA-T | 65.90367 | -22.4085 | 7 | 11 | 2017 | NA | AB |
| 2210IFC16 | Seine | Isafjordur | 6.1 | 0 | 2.12 | 0 | Arngerdareyri | ARG-S | 65.89512 | -22.38229 | 22 | 10 | 2017 | NA | AB |
| 2708?FC15 | Seine | Breidafjordur | 6.1 | 0 | 2.35 | 0 | Thorskafjordur | TOR-S | 65.584040 | -22.131605 | 5 | 9 | 2017 | NA | AA |
| 2808IFC20 | Seine | Isafjordur | 6.1 | 0 | 2.36 | 0 | Arngerdareyri | ARG-S | 65.96486 | -22.89687 | 28 | 8 | 2017 | NA | AA |
| 2110BSC1 | Seine | Strandir | 6.1 | 0 | 2.43 | 0 | Bassastadir | BAS-S | 65.99392 | -22.94096 | 21 | 10 | 2017 | NA | AA |
| 2808SSC15 | Seine | Isafjordur | 6.1 | 0 | 2.51 | 0 | Arngerdareyri | ARG-S | 65.96486 | -22.89687 | 28 | 8 | 2017 | NA | AB |
| 2708?FC19 | Seine | Breidafjordur | 6.1 | 0 | 2.9 | 0 | Thorskafjordur | TOR-S | 65.584040 | -22.131605 | 5 | 9 | 2017 | NA | AA |
| 2708?FC14 | Seine | Breidafjordur | 6.1 | 0 | 2.95 | 0 | Thorskafjordur | TOR-S | 65.584040 | -22.131605 | 5 | 9 | 2017 | NA | AA |
| A1072T10 | Trawl | Arnarfjordur | 6.2 | 0 | 2.41 | 63 | Inner_Arnarfjordur | Inner_ARN-T | 65.4476 | -23.185 | 4 | 10 | 2019 | NA | BB |
| 021177C32 | Trawl | Isafjordur | 6.2 | 0 | 1.8 | 43 | Mjoifjordur | MJO-T | 65.97533 | -22.55733 | 2 | 11 | 2017 | NA | AB |
| 2708KSc3 | Seine | Breidafjordur | 6.2 | 0 | 1.98 | 0 | Thorskafjordur | TOR-S | 65.584040 | -22.131605 | 5 | 9 | 2017 | NA | AB |
| 0711102C12 | Trawl | Isafjordur | 6.2 | 0 | 2.05 | 44 | Isafjordur | ISA-T | 65.90367 | -22.4085 | 7 | 11 | 2017 | NA | AB |
| 2708?FC12 | Seine | Breidafjordur | 6.2 | 0 | 2.52 | 0 | Thorskafjordur | TOR-S | 65.584040 | -22.131605 | 5 | 9 | 2017 | NA | AA |
| 2708?FC17 | Seine | Breidafjordur | 6.2 | 0 | 2.54 | 0 | Thorskafjordur | TOR-S | 65.584040 | -22.131605 | 5 | 9 | 2017 | NA | AA |
| 2210IFC12 | Seine | Isafjordur | 6.2 | 0 | 2.64 | 0 | Arngerdareyri | ARG-S | 65.89512 | -22.38229 | 22 | 10 | 2017 | NA | AB |
| 2808SSC6 | Seine | Isafjordur | 6.2 | 0 | 2.67 | 0 | Arngerdareyri | ARG-S | 65.96486 | -22.89687 | 28 | 8 | 2017 | NA | AB |
| 2708?FC1 | Seine | Breidafjordur | 6.2 | 0 | 3.48 | 0 | Thorskafjordur | TOR-S | 65.584040 | -22.131605 | 5 | 9 | 2017 | NA | AA |
| 2210IFC11 | Seine | Isafjordur | 6.3 | 0 | 2.06 | 0 | Arngerdareyri | ARG-S | 65.89512 | -22.38229 | 22 | 10 | 2017 | NA | AB |
| 2210BSC6 | Seine | Strandir | 6.3 | 0 | 2.12 | 0 | Bassastadir | BAS-S | 66.04037 | -22.68079 | 22 | 10 | 2017 | NA | AA |
| 2210IFC37 | Seine | Isafjordur | 6.3 | 0 | 2.13 | 0 | Arngerdareyri | ARG-S | 65.89512 | -22.38229 | 22 | 10 | 2017 | NA | BB |
| 011167C26 | Trawl | Isafjordur | 6.3 | 0 | 2.16 | 48 | Isafjordur | ISA-T | 65.92233 | -22.4065 | 1 | 11 | 2017 | NA | BB |
| 0711102C7 | Trawl | Isafjordur | 6.3 | 0 | 2.17 | 44 | Isafjordur | ISA-T | 65.90367 | -22.4085 | 7 | 11 | 2017 | NA | AB |
| 2210BSC9 | Seine | Strandir | 6.3 | 0 | 2.44 | 0 | Bassastadir | BAS-S | 66.04037 | -22.68079 | 22 | 10 | 2017 | NA | AA |
| 2210BSC10 | Seine | Strandir | 6.3 | 0 | 2.44 | 0 | Bassastadir | BAS-S | 66.04037 | -22.68079 | 22 | 10 | 2017 | NA | AA |
| 2210IFC6 | Seine | Isafjordur | 6.3 | 0 | 2.45 | 0 | Arngerdareyri | ARG-S | 65.89512 | -22.38229 | 22 | 10 | 2017 | NA | AB |
| 2210IFC32 | Seine | Isafjordur | 6.3 | 0 | 2.48 | 0 | Arngerdareyri | ARG-S | 65.89512 | -22.38229 | 22 | 10 | 2017 | NA | AB |
| 2210IFC44 | Seine | Isafjordur | 6.3 | 0 | 2.49 | 0 | Arngerdareyri | ARG-S | 65.89512 | -22.38229 | 22 | 10 | 2017 | NA | BB |
| 0509AKC12 | Seine | Arnarfjordur | 6.3 | 0 | 2.51 | 0 | Audkula | AUD-S | 65.96486 | -22.89687 | 5 | 9 | 2017 | NA | AA |
| 2708?FC8 | Seine | Breidafjordur | 6.3 | 0 | 2.57 | 0 | Thorskafjordur | TOR-S | 65.584040 | -22.131605 | 5 | 9 | 2017 | NA | AA |
| A1071T1 | Trawl | Arnarfjordur | 6.4 | 0 | 2.31 | 93 | Inner_Arnarfjordur | Inner_ARN-T | 65.4476 | -23.2435 | 4 | 10 | 2019 | NA | AB |
| A1071T6 | Trawl | Arnarfjordur | 6.4 | 0 | 2.42 | 93 | Inner_Arnarfjordur | Inner_ARN-T | 65.4476 | -23.2435 | 4 | 10 | 2019 | NA | BB |
| 011167C3 | Trawl | Isafjordur | 6.4 | 0 | 2.13 | 48 | Isafjordur | ISA-T | 65.92233 | -22.4065 | 1 | 11 | 2017 | NA | AA |
| 0711102C10 | Trawl | Isafjordur | 6.4 | 0 | 2.15 | 44 | Isafjordur | ISA-T | 65.90367 | -22.4085 | 7 | 11 | 2017 | NA | AB |
| 2210BSC4 | Seine | Strandir | 6.4 | 0 | 2.31 | 0 | Bassastadir | BAS-S | 66.04037 | -22.68079 | 22 | 10 | 2017 | NA | BB |
| 0509AKC17 | Seine | Arnarfjordur | 6.4 | 0 | 2.52 | 0 | Audkula | AUD-S | 65.96486 | -22.89687 | 5 | 9 | 2017 | NA | AA |
| 2210IFC45 | Seine | Isafjordur | 6.4 | 0 | 2.55 | 0 | Arngerdareyri | ARG-S | 65.89512 | -22.38229 | 22 | 10 | 2017 | NA | AA |
| 2210IFC9 | Seine | Isafjordur | 6.4 | 0 | 2.65 | 0 | Arngerdareyri | ARG-S | 65.89512 | -22.38229 | 22 | 10 | 2017 | NA | AA |
| 2210BSC12 | Seine | Strandir | 6.4 | 0 | NA | 0 | Bassastadir | BAS-S | 66.04037 | -22.68079 | 22 | 10 | 2017 | NA | AA |
| A1071T14 | Trawl | Arnarfjordur | 6.5 | 0 | 2.92 | 93 | Inner_Arnarfjordur | Inner_ARN-T | 65.4476 | -23.2435 | 4 | 10 | 2019 | NA | BB |
| 011167C27 | Trawl | Isafjordur | 6.5 | 0 | 2.18 | 48 | Isafjordur | ISA-T | 65.92233 | -22.4065 | 1 | 11 | 2017 | NA | AA |
| 0711102C13 | Trawl | Isafjordur | 6.5 | 0 | 2.21 | 44 | Isafjordur | ISA-T | 65.90367 | -22.4085 | 7 | 11 | 2017 | NA | AB |
| 0711102C14 | Trawl | Isafjordur | 6.5 | 0 | 2.22 | 44 | Isafjordur | ISA-T | 65.90367 | -22.4085 | 7 | 11 | 2017 | NA | BB |
| 0711102C17 | Trawl | Isafjordur | 6.5 | 0 | 2.39 | 44 | Isafjordur | ISA-T | 65.90367 | -22.4085 | 7 | 11 | 2017 | NA | BB |
| 0711102C18 | Trawl | Isafjordur | 6.5 | 0 | 2.47 | 44 | Isafjordur | ISA-T | 65.90367 | -22.4085 | 7 | 11 | 2017 | NA | AB |
| 2808IFC18 | Seine | Isafjordur | 6.5 | 0 | 2.52 | 0 | Arngerdareyri | ARG-S | 65.96486 | -22.89687 | 28 | 8 | 2017 | NA | AA |
| A1076T12 | Trawl | Arnarfjordur | 6.5 | 0 | 2.54 | 48 | Inner_Arnarfjordur | Inner_ARN-T | 65.4421 | -23.2196 | 4 | 10 | 2019 | NA | AA |
| 0509AKC4 | Seine | Arnarfjordur | 6.5 | 0 | 2.63 | 0 | Audkula | AUD-S | 65.96486 | -22.89687 | 5 | 9 | 2017 | NA | AA |
| 2608EYC11 | Seine | Strandir | 6.5 | 0 | 2.85 | 0 | Eyjar | EYJ-S | 65.98089 | -23.03125 | 26 | 8 | 2017 | NA | AB |
| A1076T2 | Trawl | Arnarfjordur | 6.6 | 0 | 2.61 | 48 | Inner_Arnarfjordur | Inner_ARN-T | 65.4421 | -23.2196 | 4 | 10 | 2019 | NA | AB |
| A1072T1 | Trawl | Arnarfjordur | 6.6 | 0 | 2.39 | 63 | Inner_Arnarfjordur | Inner_ARN-T | 65.4476 | -23.185 | 4 | 10 | 2019 | NA | BB |
| 011167C30 | Trawl | Isafjordur | 6.6 | 0 | 2.09 | 48 | Isafjordur | ISA-T | 65.92233 | -22.4065 | 1 | 11 | 2017 | NA | BB |
| 2210IFC4 | Seine | Isafjordur | 6.6 | 0 | 2.33 | 0 | Arngerdareyri | ARG-S | 65.89512 | -22.38229 | 22 | 10 | 2017 | NA | AB |
| 2708KSc7 | Seine | Breidafjordur | 6.6 | 0 | 2.36 | 0 | Thorskafjordur | TOR-S | 65.584040 | -22.131605 | 5 | 9 | 2017 | NA | AA |
| A1076T14 | Trawl | Arnarfjordur | 6.6 | 0 | 2.66 | 48 | Inner_Arnarfjordur | Inner_ARN-T | 65.4421 | -23.2196 | 4 | 10 | 2019 | NA | BB |
| 2708?FC11 | Seine | Breidafjordur | 6.6 | 0 | 3.32 | 0 | Thorskafjordur | TOR-S | 65.584040 | -22.131605 | 5 | 9 | 2017 | NA | AA |
| A1071T12 | Trawl | Arnarfjordur | 6.7 | 0 | 2.68 | 93 | Inner_Arnarfjordur | Inner_ARN-T | 65.4476 | -23.2435 | 4 | 10 | 2019 | NA | BB |
| 0711102C5 | Trawl | Isafjordur | 6.7 | 0 | 2.68 | 44 | Isafjordur | ISA-T | 65.90367 | -22.4085 | 7 | 11 | 2017 | NA | BB |
| 011167C22 | Trawl | Isafjordur | 6.7 | 0 | 2.75 | 48 | Isafjordur | ISA-T | 65.92233 | -22.4065 | 1 | 11 | 2017 | NA | AB |
| 0711106C33 | Trawl | Isafjordur | 6.7 | 0 | 2.76 | 42 | Isafjordur | ISA-T | 65.92267 | -22.4075 | 7 | 11 | 2017 | NA | AB |
| 2708?FC9 | Seine | Breidafjordur | 6.7 | 0 | 2.89 | 0 | Thorskafjordur | TOR-S | 65.584040 | -22.131605 | 5 | 9 | 2017 | NA | AA |
| 2210BSC3 | Seine | Strandir | 6.7 | 0 | 3.02 | 0 | Bassastadir | BAS-S | 66.04037 | -22.68079 | 22 | 10 | 2017 | NA | AB |
| 2110BSC5 | Seine | Strandir | 6.7 | 0 | NA | 0 | Bassastadir | BAS-S | 65.99392 | -22.94096 | 21 | 10 | 2017 | NA | AA |
| 0711106C53 | Trawl | Isafjordur | 6.8 | 0 | 2.69 | 42 | Isafjordur | ISA-T | 65.92267 | -22.4075 | 7 | 11 | 2017 | NA | AB |
| 0711106C55 | Trawl | Isafjordur | 6.8 | 0 | 3.16 | 42 | Isafjordur | ISA-T | 65.92267 | -22.4075 | 7 | 11 | 2017 | NA | BB |
| 2508BSC3 | Seine | Strandir | 6.9 | 0 | 3.18 | 0 | Bassastadir | BAS-S | 65.75949 | -23.47852 | 25 | 8 | 2017 | NA | AB |
| E95 | Seine | Isafjordur | NA | 0 | NA | 0 | Seydisfjordur | SEY-S | 65.95817 | -22.38883 | 9 | 10 | 2019 | NA | AA |
| E95 | Seine | Isafjordur | NA | 0 | NA | 0 | Seydisfjordur | SEY-S | 65.95817 | -22.38883 | 9 | 10 | 2019 | NA | AA |
| I1196T1 | Trawl | Isafjordur | 22 | 1 | 107.69 | 89 | Isafjordur | ISA-T | 66.0416667 | -22.5369444 | 7 | 10 | 2019 | NA | BB |
| 0711102C6 | Trawl | Isafjordur | 7 | 0 | 3.24 | 44 | Isafjordur | ISA-T | 65.90367 | -22.4085 | 7 | 11 | 2017 | NA | BB |
| 2708?FC16 | Seine | Breidafjordur | 7 | 0 | 3.8 | 0 | Thorskafjordur | TOR-S | 65.584040 | -22.131605 | 5 | 9 | 2017 | NA | AA |
| I1196T10 | Trawl | Isafjordur | 17 | 1 | 56.56 | 89 | Isafjordur | ISA-T | 66.0416667 | -22.5369444 | 7 | 10 | 2019 | NA | AA |
| I1196T11 | Trawl | Isafjordur | 17.3 | 1 | 62.55 | 89 | Isafjordur | ISA-T | 66.0416667 | -22.5369444 | 7 | 10 | 2019 | NA | AA |
| A1072T7 | Trawl | Arnarfjordur | 7.1 | 0 | 4.41 | 63 | Inner_Arnarfjordur | Inner_ARN-T | 65.4476 | -23.185 | 4 | 10 | 2019 | NA | BB |
| I1196T12 | Trawl | Isafjordur | 18.5 | 1 | 77.6 | 89 | Isafjordur | ISA-T | 66.0416667 | -22.5369444 | 7 | 10 | 2019 | NA | BB |
| I1196T13 | Trawl | Isafjordur | 17.2 | 1 | 62.23 | 89 | Isafjordur | ISA-T | 66.0416667 | -22.5369444 | 7 | 10 | 2019 | NA | AA |
| 0711106C60 | Trawl | Isafjordur | 7.1 | 0 | 3.36 | 42 | Isafjordur | ISA-T | 65.92267 | -22.4075 | 7 | 11 | 2017 | NA | BB |
| 0711106C61 | Trawl | Isafjordur | 7.1 | 0 | 3.4 | 42 | Isafjordur | ISA-T | 65.92267 | -22.4075 | 7 | 11 | 2017 | NA | BB |
| 0711106C50 | Trawl | Isafjordur | 7.1 | 0 | 3.51 | 42 | Isafjordur | ISA-T | 65.92267 | -22.4075 | 7 | 11 | 2017 | NA | AB |
| 2808SSC8 | Seine | Isafjordur | 7.1 | 0 | 3.54 | 0 | Arngerdareyri | ARG-S | 65.96486 | -22.89687 | 28 | 8 | 2017 | NA | AB |
| 2708KSc1 | Seine | Breidafjordur | 7.1 | 0 | 3.74 | 0 | Thorskafjordur | TOR-S | 65.584040 | -22.131605 | 5 | 9 | 2017 | NA | AB |
| 1610AKC4 | Seine | Arnarfjordur | 7.1 | 0 | 3.77 | 0 | Audkula | AUD-S | 65.83339 | -21.28872 | 16 | 10 | 2017 | NA | AA |
| 2110BSC4 | Seine | Strandir | 7.1 | 0 | 4.01 | 0 | Bassastadir | BAS-S | 65.99392 | -22.94096 | 21 | 10 | 2017 | NA | AA |
| I1196T14 | Trawl | Isafjordur | 19.4 | 1 | 107.08 | 89 | Isafjordur | ISA-T | 66.0416667 | -22.5369444 | 7 | 10 | 2019 | NA | AB |
| I1196T15 | Trawl | Isafjordur | 17.5 | 1 | 74.88 | 89 | Isafjordur | ISA-T | 66.0416667 | -22.5369444 | 7 | 10 | 2019 | NA | AB |
| A1071T8 | Trawl | Arnarfjordur | 7.2 | 0 | 3.86 | 93 | Inner_Arnarfjordur | Inner_ARN-T | 65.4476 | -23.2435 | 4 | 10 | 2019 | NA | BB |
| 011167C25 | Trawl | Isafjordur | 7.2 | 0 | 3.01 | 48 | Isafjordur | ISA-T | 65.92233 | -22.4065 | 1 | 11 | 2017 | NA | BB |
| 2210BSC11 | Seine | Strandir | 7.2 | 0 | 3.36 | 0 | Bassastadir | BAS-S | 66.04037 | -22.68079 | 22 | 10 | 2017 | NA | AA |
| 2210BSC8 | Seine | Strandir | 7.2 | 0 | 3.48 | 0 | Bassastadir | BAS-S | 66.04037 | -22.68079 | 22 | 10 | 2017 | NA | AA |
| I1196T16 | Trawl | Isafjordur | 19 | 1 | 90.72 | 89 | Isafjordur | ISA-T | 66.0416667 | -22.5369444 | 7 | 10 | 2019 | NA | AB |
| I1196T17 | Trawl | Isafjordur | 16.8 | 1 | 76.49 | 89 | Isafjordur | ISA-T | 66.0416667 | -22.5369444 | 7 | 10 | 2019 | NA | BB |
| 2210BSC13 | Seine | Strandir | 7.3 | 0 | NA | 0 | Bassastadir | BAS-S | 66.04037 | -22.68079 | 22 | 10 | 2017 | NA | AB |
| I1196T18 | Trawl | Isafjordur | 19.1 | 1 | 83.49 | 89 | Isafjordur | ISA-T | 66.0416667 | -22.5369444 | 7 | 10 | 2019 | NA | AA |
| I1196T19 | Trawl | Isafjordur | 16.8 | 1 | 56.13 | 89 | Isafjordur | ISA-T | 66.0416667 | -22.5369444 | 7 | 10 | 2019 | NA | BB |
| I1196T2 | Trawl | Isafjordur | 15.7 | 1 | 54.48 | 89 | Isafjordur | ISA-T | 66.0416667 | -22.5369444 | 7 | 10 | 2019 | NA | AA |
| 0711102C3 | Trawl | Isafjordur | 7.4 | 0 | 3.62 | 44 | Isafjordur | ISA-T | 65.90367 | -22.4085 | 7 | 11 | 2017 | NA | BB |
| 2708?FC21 | Seine | Breidafjordur | 7.4 | 0 | NA | 0 | Thorskafjordur | TOR-S | 65.584040 | -22.131605 | 5 | 9 | 2017 | NA | AB |
| 2210BSC2 | Seine | Strandir | 7.5 | 0 | 3.82 | 0 | Bassastadir | BAS-S | 66.04037 | -22.68079 | 22 | 10 | 2017 | NA | BB |
| 0711106C65 | Trawl | Isafjordur | 7.5 | 0 | 3.95 | 42 | Isafjordur | ISA-T | 65.92267 | -22.4075 | 7 | 11 | 2017 | NA | BB |
| 0711102C4 | Trawl | Isafjordur | 7.5 | 0 | 4.01 | 44 | Isafjordur | ISA-T | 65.90367 | -22.4085 | 7 | 11 | 2017 | NA | AB |
| 2508BSC11 | Seine | Strandir | 7.5 | 0 | 4.1 | 0 | Bassastadir | BAS-S | 65.75949 | -23.47852 | 25 | 8 | 2017 | NA | BB |
| I1196T20 | Trawl | Isafjordur | 17.1 | 1 | 64.01 | 89 | Isafjordur | ISA-T | 66.0416667 | -22.5369444 | 7 | 10 | 2019 | NA | AB |
| I1196T22 | Trawl | Isafjordur | 18.8 | 1 | 62.87 | 89 | Isafjordur | ISA-T | 66.0416667 | -22.5369444 | 7 | 10 | 2019 | NA | AA |
| 011167C23 | Trawl | Isafjordur | 7.6 | 0 | 3.07 | 48 | Isafjordur | ISA-T | 65.92233 | -22.4065 | 1 | 11 | 2017 | NA | BB |
| 011167C21 | Trawl | Isafjordur | 7.6 | 0 | 3.61 | 48 | Isafjordur | ISA-T | 65.92233 | -22.4065 | 1 | 11 | 2017 | NA | BB |
| 0711106C63 | Trawl | Isafjordur | 7.6 | 0 | 3.75 | 42 | Isafjordur | ISA-T | 65.92267 | -22.4075 | 7 | 11 | 2017 | NA | BB |
| 2910TFC2 | Seine | Breidafjordur | 7.6 | 0 | 4.49 | 0 | Thorskafjordur | TOR-S | 65.584040 | -22.131605 | 29 | 10 | 2017 | NA | AA |
| I1196T23 | Trawl | Isafjordur | 14.8 | 1 | 50.49 | 89 | Isafjordur | ISA-T | 66.0416667 | -22.5369444 | 7 | 10 | 2019 | NA | AA |
| A1072T13 | Trawl | Arnarfjordur | 7.7 | 0 | 4.03 | 63 | Inner_Arnarfjordur | Inner_ARN-T | 65.4476 | -23.185 | 4 | 10 | 2019 | NA | BB |
| I1196T24 | Trawl | Isafjordur | 16.2 | 1 | 41.9 | 89 | Isafjordur | ISA-T | 66.0416667 | -22.5369444 | 7 | 10 | 2019 | NA | BB |
| I1196T25 | Trawl | Isafjordur | 24.4 | 1 | 81.61 | 89 | Isafjordur | ISA-T | 66.0416667 | -22.5369444 | 7 | 10 | 2019 | NA | BB |
| 011167C18 | Trawl | Isafjordur | 7.7 | 0 | 3.6 | 48 | Isafjordur | ISA-T | 65.92233 | -22.4065 | 1 | 11 | 2017 | NA | AB |
| 0711106C58 | Trawl | Isafjordur | 7.7 | 0 | 3.91 | 42 | Isafjordur | ISA-T | 65.92267 | -22.4075 | 7 | 11 | 2017 | NA | BB |
| 011167C17 | Trawl | Isafjordur | 7.7 | 0 | 4.13 | 48 | Isafjordur | ISA-T | 65.92233 | -22.4065 | 1 | 11 | 2017 | NA | BB |
| 011167C24 | Trawl | Isafjordur | 7.7 | 0 | 4.43 | 48 | Isafjordur | ISA-T | 65.92233 | -22.4065 | 1 | 11 | 2017 | NA | AB |
| A1071T11 | Trawl | Arnarfjordur | 7.8 | 0 | 5.2 | 93 | Inner_Arnarfjordur | Inner_ARN-T | 65.4476 | -23.2435 | 4 | 10 | 2019 | NA | BB |
| A1071T7 | Trawl | Arnarfjordur | 7.8 | 0 | 4.67 | 93 | Inner_Arnarfjordur | Inner_ARN-T | 65.4476 | -23.2435 | 4 | 10 | 2019 | NA | BB |
| I1196T26 | Trawl | Isafjordur | 24 | 1 | 112.48 | 89 | Isafjordur | ISA-T | 66.0416667 | -22.5369444 | 7 | 10 | 2019 | NA | AB |
| 0711106C32 | Trawl | Isafjordur | 7.9 | 0 | 4.85 | 42 | Isafjordur | ISA-T | 65.92267 | -22.4075 | 7 | 11 | 2017 | NA | AB |
| 2210BSC1 | Seine | Strandir | 7.9 | 0 | 5.35 | 0 | Bassastadir | BAS-S | 66.04037 | -22.68079 | 22 | 10 | 2017 | NA | AB |
| I1196T27 | Trawl | Isafjordur | 20.4 | 1 | 112.98 | 89 | Isafjordur | ISA-T | 66.0416667 | -22.5369444 | 7 | 10 | 2019 | NA | AB |
| I1196T3 | Trawl | Isafjordur | 20 | 1 | 76.06 | 89 | Isafjordur | ISA-T | 66.0416667 | -22.5369444 | 7 | 10 | 2019 | NA | BB |
| I1196T4 | Trawl | Isafjordur | 18.2 | 1 | 76.25 | 89 | Isafjordur | ISA-T | 66.0416667 | -22.5369444 | 7 | 10 | 2019 | NA | AB |
| A1072T3 | Trawl | Arnarfjordur | 8 | 0 | 5.15 | 63 | Inner_Arnarfjordur | Inner_ARN-T | 65.4476 | -23.185 | 4 | 10 | 2019 | NA | BB |
| I1196T5 | Trawl | Isafjordur | 18 | 1 | 76.92 | 89 | Isafjordur | ISA-T | 66.0416667 | -22.5369444 | 7 | 10 | 2019 | NA | AB |
| I1196T6 | Trawl | Isafjordur | 18.5 | 1 | 89 | 89 | Isafjordur | ISA-T | 66.0416667 | -22.5369444 | 7 | 10 | 2019 | NA | AB |
| I1196T7 | Trawl | Isafjordur | 17.5 | 1 | 72.06 | 89 | Isafjordur | ISA-T | 66.0416667 | -22.5369444 | 7 | 10 | 2019 | NA | AA |
| I1196T8 | Trawl | Isafjordur | 20.8 | 1 | 118.6 | 89 | Isafjordur | ISA-T | 66.0416667 | -22.5369444 | 7 | 10 | 2019 | NA | AB |
| I1196T9 | Trawl | Isafjordur | 18.1 | 1 | 78.48 | 89 | Isafjordur | ISA-T | 66.0416667 | -22.5369444 | 7 | 10 | 2019 | NA | AB |
| A1071T9 | Trawl | Arnarfjordur | 8.1 | 0 | 4.98 | 93 | Inner_Arnarfjordur | Inner_ARN-T | 65.4476 | -23.2435 | 4 | 10 | 2019 | NA | BB |
| 0711106C59 | Trawl | Isafjordur | 8.1 | 0 | 3.96 | 42 | Isafjordur | ISA-T | 65.92267 | -22.4075 | 7 | 11 | 2017 | NA | BB |
| 011167C5 | Trawl | Isafjordur | 8.3 | 0 | 5.63 | 48 | Isafjordur | ISA-T | 65.92233 | -22.4065 | 1 | 11 | 2017 | NA | AB |
| A933T10 | Trawl | Arnarfjordur | 8.38 | 0 | 9.1 | 64 | Outer_Arnarfjordur | Outer_ARN-T | 65.8031 | -23.7978 | 1 | 10 | 2019 | NA | AB |
| A1072T14 | Trawl | Arnarfjordur | 8.4 | 0 | 5.86 | 63 | Inner_Arnarfjordur | Inner_ARN-T | 65.4476 | -23.185 | 4 | 10 | 2019 | NA | BB |
| 0711102C2 | Trawl | Isafjordur | 8.4 | 0 | 6.02 | 44 | Isafjordur | ISA-T | 65.90367 | -22.4085 | 7 | 11 | 2017 | NA | BB |
| 0711106C62 | Trawl | Isafjordur | 8.4 | 0 | 6.03 | 42 | Isafjordur | ISA-T | 65.92267 | -22.4075 | 7 | 11 | 2017 | NA | BB |
| A1071T10 | Trawl | Arnarfjordur | 8.5 | 0 | 5.83 | 93 | Inner_Arnarfjordur | Inner_ARN-T | 65.4476 | -23.2435 | 4 | 10 | 2019 | NA | BB |
| A1071T4 | Trawl | Arnarfjordur | 8.5 | 0 | 5.85 | 93 | Inner_Arnarfjordur | Inner_ARN-T | 65.4476 | -23.2435 | 4 | 10 | 2019 | NA | BB |
| 011167C29 | Trawl | Isafjordur | 8.5 | 0 | 4.9 | 48 | Isafjordur | ISA-T | 65.92233 | -22.4065 | 1 | 11 | 2017 | NA | BB |
| 0711106C64 | Trawl | Isafjordur | 8.5 | 0 | 5.36 | 42 | Isafjordur | ISA-T | 65.92267 | -22.4075 | 7 | 11 | 2017 | NA | BB |
| 2910TFC1 | Seine | Breidafjordur | 8.5 | 0 | 7.25 | 0 | Thorskafjordur | TOR-S | 65.584040 | -22.131605 | 29 | 10 | 2017 | NA | AA |
| 2110EYC1 | Seine | Strandir | 8.6 | 0 | 6 | 0 | Eyjar | EYJ-S | 65.83339 | -21.28872 | 21 | 10 | 2017 | NA | AA |
| 011167C8 | Trawl | Isafjordur | 8.6 | 0 | 7.72 | 48 | Isafjordur | ISA-T | 65.92233 | -22.4065 | 1 | 11 | 2017 | NA | AB |
| 011167C6 | Trawl | Isafjordur | 8.7 | 0 | 6.2 | 48 | Isafjordur | ISA-T | 65.92233 | -22.4065 | 1 | 11 | 2017 | NA | BB |
| A1071T15 | Trawl | Arnarfjordur | 8.8 | 0 | 6.14 | 93 | Inner_Arnarfjordur | Inner_ARN-T | 65.4476 | -23.2435 | 4 | 10 | 2019 | NA | BB |
| 0711106C31 | Trawl | Isafjordur | 8.9 | 0 | 6.8 | 42 | Isafjordur | ISA-T | 65.92267 | -22.4075 | 7 | 11 | 2017 | NA | BB |
| 0711106C27 | Trawl | Isafjordur | 8.9 | 0 | 6.9 | 42 | Isafjordur | ISA-T | 65.92267 | -22.4075 | 7 | 11 | 2017 | NA | BB |
| 011167C4 | Trawl | Isafjordur | 9 | 0 | 6.68 | 48 | Isafjordur | ISA-T | 65.92233 | -22.4065 | 1 | 11 | 2017 | NA | AB |
| 0711106C36 | Trawl | Isafjordur | 9.2 | 0 | 6.39 | 42 | Isafjordur | ISA-T | 65.92267 | -22.4075 | 7 | 11 | 2017 | NA | BB |
| 011167C10 | Trawl | Isafjordur | 9.2 | 0 | 6.81 | 48 | Isafjordur | ISA-T | 65.92233 | -22.4065 | 1 | 11 | 2017 | NA | AB |
| 0711106C52 | Trawl | Isafjordur | 9.2 | 0 | 8 | 42 | Isafjordur | ISA-T | 65.92267 | -22.4075 | 7 | 11 | 2017 | NA | BB |
| 0711102C1 | Trawl | Isafjordur | 9.3 | 0 | 8.64 | 44 | Isafjordur | ISA-T | 65.90367 | -22.4085 | 7 | 11 | 2017 | NA | BB |
| 0711106C40 | Trawl | Isafjordur | 9.4 | 0 | 9.74 | 42 | Isafjordur | ISA-T | 65.92267 | -22.4075 | 7 | 11 | 2017 | NA | BB |
| 021177C14 | Trawl | Isafjordur | 9.5 | 0 | 8.77 | 43 | Mjoifjordur | MJO-T | 65.97533 | -22.55733 | 2 | 11 | 2017 | NA | AB |
| 021177C13 | Trawl | Isafjordur | 9.5 | 0 | 8.87 | 43 | Mjoifjordur | MJO-T | 65.97533 | -22.55733 | 2 | 11 | 2017 | NA | AB |
| 011167C19 | Trawl | Isafjordur | 9.6 | 0 | 8.24 | 48 | Isafjordur | ISA-T | 65.92233 | -22.4065 | 1 | 11 | 2017 | NA | BB |
| I1322T3 | Trawl | Isafjordur | 6.8 | 0 | 3.15 | 75 | Isafjordur | ISA-T | 65.572 | -22.4802 | 10 | 10 | 2019 | NA | AB |
| I1322T30 | Trawl | Isafjordur | 8.9 | 0 | 7.02 | 75 | Isafjordur | ISA-T | 65.572 | -22.4802 | 10 | 10 | 2019 | NA | AB |
| 0711106C49 | Trawl | Isafjordur | 9.7 | 0 | 7.78 | 42 | Isafjordur | ISA-T | 65.92267 | -22.4075 | 7 | 11 | 2017 | NA | BB |
| 0711106C56 | Trawl | Isafjordur | 9.7 | 0 | 7.95 | 42 | Isafjordur | ISA-T | 65.92267 | -22.4075 | 7 | 11 | 2017 | NA | AB |
| 0711106C57 | Trawl | Isafjordur | 9.8 | 0 | 8.52 | 42 | Isafjordur | ISA-T | 65.92267 | -22.4075 | 7 | 11 | 2017 | NA | BB |
| I1322T5 | Trawl | Isafjordur | 8 | 0 | 5.47 | 75 | Isafjordur | ISA-T | 65.572 | -22.4802 | 10 | 10 | 2019 | NA | BB |
| BS268BS65 | Seine | Strandir | NA | 0 | NA | 0 | Bassastadir | BAS-S | 65.8945 | -22.387 | 26 | 8 | 2019 | NA | AA |
| E21 | Seine | Strandir | NA | 0 | NA | 0 | Bassastadir | BAS-S | 65.8945 | -22.387 | 12 | 10 | 2019 | NA | AA |
| 2608TFC1 | Seine | Breidafjordur | NA | 0 | NA | 0 | Thorskafjordur | TOR-S | 65.584040 | -22.131605 | 5 | 9 | 2017 | NA | AA |
| 2608TFC2 | Seine | Breidafjordur | NA | 0 | NA | 0 | Thorskafjordur | TOR-S | 65.584040 | -22.131605 | 5 | 9 | 2017 | NA | AA |
| 2608TFC5 | Seine | Breidafjordur | NA | 0 | NA | 0 | Thorskafjordur | TOR-S | 65.584040 | -22.131605 | 5 | 9 | 2017 | NA | AA |
| 2608TFC2 | Seine | Breidafjordur | NA | 0 | NA | 0 | Thorskafjordur | TOR-S | 65.584040 | -22.131605 | 5 | 9 | 2017 | NA | AA |
| 0509VFC1 | Seine | Breidafjordur | NA | 0 | NA | 0 | Vatnsfjordur | VAT-S | 65.52899 | -23.18938 | 5 | 9 | 2017 | NA | AA |
| 0509VFC2 | Seine | Breidafjordur | NA | 0 | NA | 0 | Vatnsfjordur | VAT-S | 65.52899 | -23.18938 | 5 | 9 | 2017 | NA | AA |
| BS268BS67 | Seine | Strandir | NA | 0 | NA | 0 | Bassastadir | BAS-S | 65.8945 | -22.387 | 26 | 8 | 2019 | NA | AB |
| 2608TFC3 | Seine | Breidafjordur | NA | 0 | NA | 0 | Thorskafjordur | TOR-S | 65.584040 | -22.131605 | 5 | 9 | 2017 | NA | AB |
| 2608TFC1 | Seine | Breidafjordur | NA | 0 | NA | 0 | Thorskafjordur | TOR-S | 65.584040 | -22.131605 | 5 | 9 | 2017 | NA | AB |
| 2608TFC3 | Seine | Breidafjordur | NA | 0 | NA | 0 | Thorskafjordur | TOR-S | 65.584040 | -22.131605 | 5 | 9 | 2017 | NA | AB |
| 2608TFC4 | Seine | Breidafjordur | NA | 0 | NA | 0 | Thorskafjordur | TOR-S | 65.584040 | -22.131605 | 5 | 9 | 2017 | NA | AB |
| 2608TFC5 | Seine | Breidafjordur | NA | 0 | NA | 0 | Thorskafjordur | TOR-S | 65.584040 | -22.131605 | 5 | 9 | 2017 | NA | AB |
| 2210TFC1 | Seine | Breidafjordur | NA | 0 | NA | 0 | Thorskafjordur | TOR-S | 65.584040 | -22.131605 | 22 | 10 | 2017 | NA | AB |
| 0509VFC3 | Seine | Breidafjordur | NA | 0 | NA | 0 | Vatnsfjordur | VAT-S | 65.52899 | -23.18938 | 5 | 9 | 2017 | NA | AB |
| 0509VFC5 | Seine | Breidafjordur | NA | 0 | NA | 0 | Vatnsfjordur | VAT-S | 65.52899 | -23.18938 | 5 | 9 | 2017 | NA | AB |
| 0509VFC1 | Seine | Breidafjordur | NA | 0 | NA | 0 | Vatnsfjordur | VAT-S | 65.52899 | -23.18938 | 5 | 9 | 2017 | NA | AB |
| 0509VFC2 | Seine | Breidafjordur | NA | 0 | NA | 0 | Vatnsfjordur | VAT-S | 65.52899 | -23.18938 | 5 | 9 | 2017 | NA | AB |
| 0509VFC3 | Seine | Breidafjordur | NA | 0 | NA | 0 | Vatnsfjordur | VAT-S | 65.52899 | -23.18938 | 5 | 9 | 2017 | NA | AB |
| 0509VFC5 | Seine | Breidafjordur | NA | 0 | NA | 0 | Vatnsfjordur | VAT-S | 65.52899 | -23.18938 | 5 | 9 | 2017 | NA | AB |
| 0509VFC4 | Seine | Breidafjordur | NA | 0 | NA | 0 | Vatnsfjordur | VAT-S | 65.52899 | -23.18938 | 5 | 9 | 2017 | NA | BB |
| A1244T11 | Trawl | Isafjordur | 6.6 | 0 | 2.59 | 63 | Skotufjordur | SKO-T | 65.5868 | -22.2582 | 8 | 10 | 2019 | Offshore | AB |
| A1244T12 | Trawl | Isafjordur | 8.5 | 0 | 6.11 | 63 | Skotufjordur | SKO-T | 65.5868 | -22.2582 | 8 | 10 | 2019 | Offshore | AB |
| A1244T15 | Trawl | Isafjordur | 8.9 | 0 | 7.18 | 63 | Skotufjordur | SKO-T | 65.5868 | -22.2582 | 8 | 10 | 2019 | Offshore | AB |
| A1244T19 | Trawl | Isafjordur | 7 | 0 | 4.11 | 63 | Skotufjordur | SKO-T | 65.5868 | -22.2582 | 8 | 10 | 2019 | Offshore | AB |
| A1244T20 | Trawl | Isafjordur | 6 | 0 | 1.96 | 63 | Skotufjordur | SKO-T | 65.5868 | -22.2582 | 8 | 10 | 2019 | Offshore | BB |
| A1244T3 | Trawl | Isafjordur | 6.2 | 0 | 2.51 | 63 | Skotufjordur | SKO-T | 65.5868 | -22.2582 | 8 | 10 | 2019 | Offshore | BB |
| A1244T4 | Trawl | Isafjordur | 6.8 | 0 | 3.2 | 63 | Skotufjordur | SKO-T | 65.5868 | -22.2582 | 8 | 10 | 2019 | Offshore | BB |
| A1244T5 | Trawl | Isafjordur | 8 | 0 | 4.68 | 63 | Skotufjordur | SKO-T | 65.5868 | -22.2582 | 8 | 10 | 2019 | Offshore | BB |
| A1244T6 | Trawl | Isafjordur | NA | 0 | NA | 63 | Skotufjordur | SKO-T | 65.5868 | -22.2582 | 8 | 10 | 2019 | Offshore | NA |
| A1244T7 | Trawl | Isafjordur | 9.1 | 0 | 8.18 | 63 | Skotufjordur | SKO-T | 65.5868 | -22.2582 | 8 | 10 | 2019 | Offshore | BB |
| A1244T8 | Trawl | Isafjordur | 8.2 | 0 | 5.56 | 63 | Skotufjordur | SKO-T | 65.5868 | -22.2582 | 8 | 10 | 2019 | Offshore | BB |
| A1244T9 | Trawl | Isafjordur | 8 | 0 | 5.71 | 63 | Skotufjordur | SKO-T | 65.5868 | -22.2582 | 8 | 10 | 2019 | Offshore | BB |
| A1249T1 | Trawl | Isafjordur | 5.9 | 0 | 2.12 | 48 | Skotufjordur | SKO-T | 65.5509 | -22.2432 | 8 | 10 | 2019 | Offshore | BB |
| A1249T11 | Trawl | Isafjordur | 6.9 | 0 | 3.65 | 48 | Skotufjordur | SKO-T | 65.5509 | -22.2432 | 8 | 10 | 2019 | Offshore | BB |
| A1249T13 | Trawl | Isafjordur | 7.1 | 0 | 3.72 | 48 | Skotufjordur | SKO-T | 65.5509 | -22.2432 | 8 | 10 | 2019 | Offshore | AB |
| A1249T14 | Trawl | Isafjordur | 7.1 | 0 | 3.6 | 48 | Skotufjordur | SKO-T | 65.5509 | -22.2432 | 8 | 10 | 2019 | Offshore | AB |
| BS268BS51 | Seine | Strandir | 3.3 | 0 | 0.34 | 0 | Bassastadir | BAS-S | 65.8945 | -22.387 | 26 | 8 | 2019 | Offshore | BB |
| BS268BS26 | Seine | Strandir | 3.5 | 0 | 0.4 | 0 | Bassastadir | BAS-S | 65.8945 | -22.387 | 26 | 8 | 2019 | Offshore | AB |
| A1249T2 | Trawl | Isafjordur | 6 | 0 | 2.53 | 48 | Skotufjordur | SKO-T | 65.5509 | -22.2432 | 8 | 10 | 2019 | Offshore | AB |
| A1249T20 | Trawl | Isafjordur | 8.5 | 0 | 5.44 | 48 | Skotufjordur | SKO-T | 65.5509 | -22.2432 | 8 | 10 | 2019 | Offshore | BB |
| A1249T3 | Trawl | Isafjordur | 6 | 0 | 2.65 | 48 | Skotufjordur | SKO-T | 65.5509 | -22.2432 | 8 | 10 | 2019 | Offshore | BB |
| A933T4 | Trawl | Arnarfjordur | 4.13 | 0 | 7.4 | 64 | Outer_Arnarfjordur | Outer_ARN-T | 65.8031 | -23.7978 | 1 | 10 | 2019 | Offshore | BB |
| A1249T5 | Trawl | Isafjordur | 6.5 | 0 | 2.8 | 48 | Skotufjordur | SKO-T | 65.5509 | -22.2432 | 8 | 10 | 2019 | Offshore | AB |
| BS268BS66 | Seine | Strandir | 4.7 | 0 | 0.97 | 0 | Bassastadir | BAS-S | 65.8945 | -22.387 | 26 | 8 | 2019 | Offshore | AB |
| A1249T7 | Trawl | Isafjordur | 6.7 | 0 | 2.93 | 48 | Skotufjordur | SKO-T | 65.5509 | -22.2432 | 8 | 10 | 2019 | Offshore | BB |
| A1249T9 | Trawl | Isafjordur | 6.9 | 0 | 3.69 | 48 | Skotufjordur | SKO-T | 65.5509 | -22.2432 | 8 | 10 | 2019 | Offshore | BB |
| A1282T1 | Trawl | Isafjordur | NA | 0 | NA | 68 | Isafjordur | ISA-T | 65.9833 | -22.4923 | 9 | 10 | 2019 | Offshore | NA |
| A1282T12 | Trawl | Isafjordur | NA | 0 | NA | 68 | Isafjordur | ISA-T | 65.9833 | -22.4923 | 9 | 10 | 2019 | Offshore | NA |
| A933T9 | Trawl | Arnarfjordur | 5.34 | 0 | 7.9 | 64 | Outer_Arnarfjordur | Outer_ARN-T | 65.8031 | -23.7978 | 1 | 10 | 2019 | Offshore | BB |
| A933T11 | Trawl | Arnarfjordur | 5.38 | 0 | 7.9 | 64 | Outer_Arnarfjordur | Outer_ARN-T | 65.8031 | -23.7978 | 1 | 10 | 2019 | Offshore | BB |
| A1282T15 | Trawl | Isafjordur | NA | 0 | NA | 68 | Isafjordur | ISA-T | 65.9833 | -22.4923 | 9 | 10 | 2019 | Offshore | NA |
| A1282T16 | Trawl | Isafjordur | NA | 0 | NA | 68 | Isafjordur | ISA-T | 65.9833 | -22.4923 | 9 | 10 | 2019 | Offshore | NA |
| A1282T17 | Trawl | Isafjordur | NA | 0 | NA | 68 | Isafjordur | ISA-T | 65.9833 | -22.4923 | 9 | 10 | 2019 | Offshore | NA |
| A1282T2 | Trawl | Isafjordur | NA | 0 | NA | 68 | Isafjordur | ISA-T | 65.9833 | -22.4923 | 9 | 10 | 2019 | Offshore | NA |
| A1017T9 | Trawl | Arnarfjordur | 5.6 | 0 | 1.87 | 85 | Inner_Arnarfjordur | Inner_ARN-T | 65.4416 | -23.3048 | 3 | 10 | 2019 | Offshore | BB |
| A1282T4 | Trawl | Isafjordur | NA | 0 | NA | 68 | Isafjordur | ISA-T | 65.9833 | -22.4923 | 9 | 10 | 2019 | Offshore | NA |
| A1282T5 | Trawl | Isafjordur | NA | 0 | NA | 68 | Isafjordur | ISA-T | 65.9833 | -22.4923 | 9 | 10 | 2019 | Offshore | NA |
| A1282T6 | Trawl | Isafjordur | NA | 0 | NA | 68 | Isafjordur | ISA-T | 65.9833 | -22.4923 | 9 | 10 | 2019 | Offshore | NA |
| A1282T8 | Trawl | Isafjordur | NA | 0 | NA | 68 | Isafjordur | ISA-T | 65.9833 | -22.4923 | 9 | 10 | 2019 | Offshore | NA |
| A1282T9 | Trawl | Isafjordur | NA | 0 | NA | 68 | Isafjordur | ISA-T | 65.9833 | -22.4923 | 9 | 10 | 2019 | Offshore | NA |
| A1322T26 | Trawl | Isafjordur | NA | 0 | NA | 76 | Isafjordur | ISA-T | 65.573 | -22.4803 | 10 | 10 | 2019 | Offshore | NA |
| A1322T31 | Trawl | Isafjordur | NA | 0 | NA | 80 | Isafjordur | ISA-T | 65.577 | -22.4807 | 10 | 10 | 2019 | Offshore | NA |
| E20 | Seine | Isafjordur | NA | 0 | NA | 0 | Seydisfjordur | SEY-S | 65.95817 | -22.38883 | 9 | 10 | 2019 | Offshore | BB |
| A980T1 | Trawl | Arnarfjordur | 6.5 | 0 | 3.42 | 58 | Outer_Arnarfjordur | Outer_ARN-T | 65.4243 | -23.322 | 2 | 10 | 2019 | Offshore | AB |
| E57 | Seine | Isafjordur | NA | 0 | NA | 0 | Seydisfjordur | SEY-S | 65.95817 | -22.38883 | 9 | 10 | 2019 | Offshore | AB |
| A1076T15 | Trawl | Arnarfjordur | 6.7 | 0 | 2.61 | 48 | Inner_Arnarfjordur | Inner_ARN-T | 65.4421 | -23.2196 | 4 | 10 | 2019 | Offshore | BB |
| A1076T5 | Trawl | Arnarfjordur | 6.7 | 0 | 3.12 | 48 | Inner_Arnarfjordur | Inner_ARN-T | 65.4421 | -23.2196 | 4 | 10 | 2019 | Offshore | AB |
| A1076T1 | Trawl | Arnarfjordur | 6.8 | 0 | 2.56 | 48 | Inner_Arnarfjordur | Inner_ARN-T | 65.4421 | -23.2196 | 4 | 10 | 2019 | Offshore | BB |
| A933T8 | Trawl | Arnarfjordur | 6.86 | 0 | 8.8 | 64 | Outer_Arnarfjordur | Outer_ARN-T | 65.8031 | -23.7978 | 1 | 10 | 2019 | Offshore | BB |
| E76 | Seine | Isafjordur | NA | 0 | NA | 0 | Seydisfjordur | SEY-S | 65.95817 | -22.38883 | 9 | 10 | 2019 | Offshore | AB |
| A933T14 | Trawl | Arnarfjordur | 7.11 | 0 | 8.6 | 64 | Outer_Arnarfjordur | Outer_ARN-T | 65.8031 | -23.7978 | 1 | 10 | 2019 | Offshore | BB |
| A1076T3 | Trawl | Arnarfjordur | 7.2 | 0 | 3.41 | 48 | Inner_Arnarfjordur | Inner_ARN-T | 65.4421 | -23.2196 | 4 | 10 | 2019 | Offshore | AB |
| A980T2 | Trawl | Arnarfjordur | 7.4 | 0 | 5.18 | 58 | Outer_Arnarfjordur | Outer_ARN-T | 65.4243 | -23.322 | 2 | 10 | 2019 | Offshore | BB |
| A933T1 | Trawl | Arnarfjordur | 7.43 | 0 | 8.9 | 64 | Outer_Arnarfjordur | Outer_ARN-T | 65.8031 | -23.7978 | 1 | 10 | 2019 | Offshore | BB |
| A933T5 | Trawl | Arnarfjordur | 7.49 | 0 | 9 | 64 | Outer_Arnarfjordur | Outer_ARN-T | 65.8031 | -23.7978 | 1 | 10 | 2019 | Offshore | BB |
| A1017T7 | Trawl | Arnarfjordur | 7.5 | 0 | 4.07 | 85 | Inner_Arnarfjordur | Inner_ARN-T | 65.4416 | -23.3048 | 3 | 10 | 2019 | Offshore | BB |
| A933T12 | Trawl | Arnarfjordur | 7.5 | 0 | 9.1 | 64 | Outer_Arnarfjordur | Outer_ARN-T | 65.8031 | -23.7978 | 1 | 10 | 2019 | Offshore | BB |
| A980T3 | Trawl | Arnarfjordur | 7.6 | 0 | 4.75 | 58 | Outer_Arnarfjordur | Outer_ARN-T | 65.4243 | -23.322 | 2 | 10 | 2019 | Offshore | BB |
| A980T4 | Trawl | Arnarfjordur | 7.6 | 0 | 4.92 | 58 | Outer_Arnarfjordur | Outer_ARN-T | 65.4243 | -23.322 | 2 | 10 | 2019 | Offshore | AB |
| A933T15 | Trawl | Arnarfjordur | 7.79 | 0 | 8.8 | 64 | Outer_Arnarfjordur | Outer_ARN-T | 65.8031 | -23.7978 | 1 | 10 | 2019 | Offshore | BB |
| A1017T14 | Trawl | Arnarfjordur | 7.8 | 0 | 5.19 | 85 | Inner_Arnarfjordur | Inner_ARN-T | 65.4416 | -23.3048 | 3 | 10 | 2019 | Offshore | BB |
| A1076T4 | Trawl | Arnarfjordur | 8 | 0 | 5.18 | 48 | Inner_Arnarfjordur | Inner_ARN-T | 65.4421 | -23.2196 | 4 | 10 | 2019 | Offshore | AB |
| A980T5 | Trawl | Arnarfjordur | 8 | 0 | 5.79 | 58 | Outer_Arnarfjordur | Outer_ARN-T | 65.4243 | -23.322 | 2 | 10 | 2019 | Offshore | BB |
| I1322T1 | Trawl | Isafjordur | 10.5 | 0 | 12.06 | 75 | Isafjordur | ISA-T | 65.572 | -22.4802 | 10 | 10 | 2019 | Offshore | BB |
| A1017T4 | Trawl | Arnarfjordur | 8.2 | 0 | 6.08 | 85 | Inner_Arnarfjordur | Inner_ARN-T | 65.4416 | -23.3048 | 3 | 10 | 2019 | Offshore | BB |
| I1322T10 | Trawl | Isafjordur | 8.2 | 0 | 5.72 | 75 | Isafjordur | ISA-T | 65.572 | -22.4802 | 10 | 10 | 2019 | Offshore | BB |
| A980T7 | Trawl | Arnarfjordur | 8.2 | 0 | 6.06 | 58 | Outer_Arnarfjordur | Outer_ARN-T | 65.4243 | -23.322 | 2 | 10 | 2019 | Offshore | BB |
| A980T8 | Trawl | Arnarfjordur | 8.2 | 0 | 5.2 | 58 | Outer_Arnarfjordur | Outer_ARN-T | 65.4243 | -23.322 | 2 | 10 | 2019 | Offshore | AB |
| I1322T11 | Trawl | Isafjordur | 8.8 | 0 | 6.76 | 75 | Isafjordur | ISA-T | 65.572 | -22.4802 | 10 | 10 | 2019 | Offshore | BB |
| I1322T12 | Trawl | Isafjordur | 8.3 | 0 | 5.59 | 75 | Isafjordur | ISA-T | 65.572 | -22.4802 | 10 | 10 | 2019 | Offshore | AB |
| A933T17 | Trawl | Arnarfjordur | 8.28 | 0 | 9.2 | 64 | Outer_Arnarfjordur | Outer_ARN-T | 65.8031 | -23.7978 | 1 | 10 | 2019 | Offshore | BB |
| I1322T14 | Trawl | Isafjordur | 8.1 | 0 | 6.08 | 75 | Isafjordur | ISA-T | 65.572 | -22.4802 | 10 | 10 | 2019 | Offshore | BB |
| A980T9 | Trawl | Arnarfjordur | 8.4 | 0 | 6.34 | 58 | Outer_Arnarfjordur | Outer_ARN-T | 65.4243 | -23.322 | 2 | 10 | 2019 | Offshore | BB |
| A933T7 | Trawl | Arnarfjordur | 8.45 | 0 | 9.1 | 64 | Outer_Arnarfjordur | Outer_ARN-T | 65.8031 | -23.7978 | 1 | 10 | 2019 | Offshore | BB |
| I1322T15 | Trawl | Isafjordur | 7.6 | 0 | 4.43 | 75 | Isafjordur | ISA-T | 65.572 | -22.4802 | 10 | 10 | 2019 | Offshore | BB |
| A980T10 | Trawl | Arnarfjordur | 8.5 | 0 | 6.87 | 58 | Outer_Arnarfjordur | Outer_ARN-T | 65.4243 | -23.322 | 2 | 10 | 2019 | Offshore | BB |
| I1322T17 | Trawl | Isafjordur | NA | 0 | NA | 76 | Isafjordur | ISA-T | 65.573 | -22.4803 | 10 | 10 | 2019 | Offshore | NA |
| A1017T15 | Trawl | Arnarfjordur | 8.6 | 0 | 6.09 | 85 | Inner_Arnarfjordur | Inner_ARN-T | 65.4416 | -23.3048 | 3 | 10 | 2019 | Offshore | BB |
| I1322T18 | Trawl | Isafjordur | 7.7 | 0 | 4.59 | 75 | Isafjordur | ISA-T | 65.572 | -22.4802 | 10 | 10 | 2019 | Offshore | BB |
| A1017T6 | Trawl | Arnarfjordur | 8.7 | 0 | 6.53 | 85 | Inner_Arnarfjordur | Inner_ARN-T | 65.4416 | -23.3048 | 3 | 10 | 2019 | Offshore | BB |
| I1322T19 | Trawl | Isafjordur | 8.7 | 0 | 7.4 | 75 | Isafjordur | ISA-T | 65.572 | -22.4802 | 10 | 10 | 2019 | Offshore | BB |
| A980T11 | Trawl | Arnarfjordur | 8.8 | 0 | 7.65 | 58 | Outer_Arnarfjordur | Outer_ARN-T | 65.4243 | -23.322 | 2 | 10 | 2019 | Offshore | BB |
| A980T12 | Trawl | Arnarfjordur | 8.8 | 0 | 6.97 | 58 | Outer_Arnarfjordur | Outer_ARN-T | 65.4243 | -23.322 | 2 | 10 | 2019 | Offshore | BB |
| I1322T2 | Trawl | Isafjordur | 8.3 | 0 | 5.29 | 75 | Isafjordur | ISA-T | 65.572 | -22.4802 | 10 | 10 | 2019 | Offshore | BB |
| A933T18 | Trawl | Arnarfjordur | 8.88 | 0 | 9.3 | 64 | Outer_Arnarfjordur | Outer_ARN-T | 65.8031 | -23.7978 | 1 | 10 | 2019 | Offshore | BB |
| I1322T20 | Trawl | Isafjordur | 6.9 | 0 | 3.63 | 75 | Isafjordur | ISA-T | 65.572 | -22.4802 | 10 | 10 | 2019 | Offshore | BB |
| A980T13 | Trawl | Arnarfjordur | 8.9 | 0 | 6.92 | 58 | Outer_Arnarfjordur | Outer_ARN-T | 65.4243 | -23.322 | 2 | 10 | 2019 | Offshore | BB |
| I1322T21 | Trawl | Isafjordur | 9.7 | 0 | 8.93 | 75 | Isafjordur | ISA-T | 65.572 | -22.4802 | 10 | 10 | 2019 | Offshore | AB |
| I1322T24 | Trawl | Isafjordur | 10.4 | 0 | 10.95 | 75 | Isafjordur | ISA-T | 65.572 | -22.4802 | 10 | 10 | 2019 | Offshore | BB |
| I1322T27 | Trawl | Isafjordur | 9 | 0 | 6.96 | 77 | Isafjordur | ISA-T | 65.574 | -22.4804 | 10 | 10 | 2019 | Offshore | BB |
| I1322T28 | Trawl | Isafjordur | 8.2 | 0 | 5.07 | 78 | Isafjordur | ISA-T | 65.575 | -22.4805 | 10 | 10 | 2019 | Offshore | BB |
| A933T16 | Trawl | Arnarfjordur | 9.13 | 0 | 9.7 | 64 | Outer_Arnarfjordur | Outer_ARN-T | 65.8031 | -23.7978 | 1 | 10 | 2019 | Offshore | BB |
| A1017T5 | Trawl | Arnarfjordur | 9.2 | 0 | 8.8 | 85 | Inner_Arnarfjordur | Inner_ARN-T | 65.4416 | -23.3048 | 3 | 10 | 2019 | Offshore | BB |
| I1322T29 | Trawl | Isafjordur | 9.8 | 0 | 9.28 | 79 | Isafjordur | ISA-T | 65.576 | -22.4806 | 10 | 10 | 2019 | Offshore | BB |
| A933T3 | Trawl | Arnarfjordur | 9.22 | 0 | 9.7 | 64 | Outer_Arnarfjordur | Outer_ARN-T | 65.8031 | -23.7978 | 1 | 10 | 2019 | Offshore | BB |
| A933T19 | Trawl | Arnarfjordur | 9.5 | 0 | 9.5 | 64 | Outer_Arnarfjordur | Outer_ARN-T | 65.8031 | -23.7978 | 1 | 10 | 2019 | Offshore | BB |
| A980T16 | Trawl | Arnarfjordur | 9.5 | 0 | 10.12 | 58 | Outer_Arnarfjordur | Outer_ARN-T | 65.4243 | -23.322 | 2 | 10 | 2019 | Offshore | BB |
| A980T17 | Trawl | Arnarfjordur | 9.6 | 0 | 9.72 | 58 | Outer_Arnarfjordur | Outer_ARN-T | 65.4243 | -23.322 | 2 | 10 | 2019 | Offshore | BB |
| A980T19 | Trawl | Arnarfjordur | 9.6 | 0 | 10.48 | 58 | Outer_Arnarfjordur | Outer_ARN-T | 65.4243 | -23.322 | 2 | 10 | 2019 | Offshore | BB |
| A1017T8 | Trawl | Arnarfjordur | 9.7 | 0 | 10.2 | 85 | Inner_Arnarfjordur | Inner_ARN-T | 65.4416 | -23.3048 | 3 | 10 | 2019 | Offshore | BB |
| A980T20 | Trawl | Arnarfjordur | 9.7 | 0 | 11.22 | 58 | Outer_Arnarfjordur | Outer_ARN-T | 65.4243 | -23.322 | 2 | 10 | 2019 | Offshore | AB |
| I1322T32 | Trawl | Isafjordur | 9.1 | 0 | 7.6 | 81 | Isafjordur | ISA-T | 65.578 | -22.4808 | 10 | 10 | 2019 | Offshore | BB |
| I1322T33 | Trawl | Isafjordur | 8.9 | 0 | 7.1 | 82 | Isafjordur | ISA-T | 65.579 | -22.4809 | 10 | 10 | 2019 | Offshore | BB |
| I1322T34 | Trawl | Isafjordur | NA | 0 | NA | 83 | Isafjordur | ISA-T | 65.580 | -22.4810 | 10 | 10 | 2019 | Offshore | NA |
| I1322T35 | Trawl | Isafjordur | 9.1 | 0 | 7.68 | 75 | Isafjordur | ISA-T | 65.572 | -22.4802 | 10 | 10 | 2019 | Offshore | BB |
| I1322T36 | Trawl | Isafjordur | 8.6 | 0 | 6.93 | 75 | Isafjordur | ISA-T | 65.572 | -22.4802 | 10 | 10 | 2019 | Offshore | BB |
| I1322T37 | Trawl | Isafjordur | 8 | 0 | 5.26 | 75 | Isafjordur | ISA-T | 65.572 | -22.4802 | 10 | 10 | 2019 | Offshore | BB |
| I1322T38 | Trawl | Isafjordur | 9.2 | 0 | 7.62 | 75 | Isafjordur | ISA-T | 65.572 | -22.4802 | 10 | 10 | 2019 | Offshore | BB |
| I1322T39 | Trawl | Isafjordur | 8.5 | 0 | 6.75 | 75 | Isafjordur | ISA-T | 65.572 | -22.4802 | 10 | 10 | 2019 | Offshore | AB |
| I1322T4 | Trawl | Isafjordur | 10.8 | 0 | 13.8 | 75 | Isafjordur | ISA-T | 65.572 | -22.4802 | 10 | 10 | 2019 | Offshore | BB |
| I1322T40 | Trawl | Isafjordur | 7.7 | 0 | 4.37 | 75 | Isafjordur | ISA-T | 65.572 | -22.4802 | 10 | 10 | 2019 | Offshore | BB |
| I1322T6 | Trawl | Isafjordur | 9.7 | 0 | 9.23 | 75 | Isafjordur | ISA-T | 65.572 | -22.4802 | 10 | 10 | 2019 | Offshore | AA |
| I1322T7 | Trawl | Isafjordur | 7.8 | 0 | 4.52 | 75 | Isafjordur | ISA-T | 65.572 | -22.4802 | 10 | 10 | 2019 | Offshore | BB |
| SF229BS1 | Seine | Isafjordur | 5.8 | 0 | 1.72 | 0 | Seydisfjordur | SEY-S | 65.95817 | -22.38883 | 22 | 9 | 2019 | Offshore | AB |
| SF229BS10 | Seine | Isafjordur | 5.5 | 0 | 1.72 | 0 | Seydisfjordur | SEY-S | 65.95817 | -22.38883 | 22 | 9 | 2019 | Offshore | AB |
| SF59BS10 | Seine | Isafjordur | 4.3 | 0 | 0.76 | 0 | Seydisfjordur | SEY-S | 65.95817 | -22.38883 | 5 | 9 | 2019 | Offshore | BB |
| A1017T10 | Trawl | Arnarfjordur | NA | N | NA | 85 | Inner_Arnarfjordur | Inner_ARN-T | 65.4416 | -23.3048 | 3 | 10 | 2019 | Offshore | NA |
| A1017T11 | Trawl | Arnarfjordur | NA | N | NA | 86 | Inner_Arnarfjordur | Inner_ARN-T | 65.4417 | -23.3049 | 3 | 10 | 2019 | Offshore | NA |
| A1017T2 | Trawl | Arnarfjordur | NA | N | NA | 85 | Inner_Arnarfjordur | Inner_ARN-T | 65.4416 | -23.3048 | 3 | 10 | 2019 | Offshore | NA |
| A1017T3 | Trawl | Arnarfjordur | NA | N | NA | 85 | Inner_Arnarfjordur | Inner_ARN-T | 65.4416 | -23.3048 | 3 | 10 | 2019 | Offshore | NA |
| SF59BS20 | Seine | Isafjordur | NA | 0 | NA | 0 | Seydisfjordur | SEY-S | 65.95818 | -22.38884 | 5 | 9 | 2019 | Offshore | NA |
| A980T15 | Trawl | Arnarfjordur | NA | 0 | NA | 59 | Outer_Arnarfjordur | Outer_ARN-T | 65.4244 | -23.323 | 2 | 10 | 2019 | Offshore | NA |
| E1 | Seine | Strandir | NA | 0 | NA | 0 | Bassastadir | BAS-S | 65.8945 | -22.387 | 12 | 10 | 2019 | Offshore | NA |
| E35 | Seine | Strandir | NA | 0 | NA | 0 | Bassastadir | BAS-S | 65.8945 | -22.387 | 12 | 10 | 2019 | Offshore | AA |
| E54 | Seine | Strandir | NA | 0 | NA | 0 | Bassastadir | BAS-S | 65.8945 | -22.387 | 12 | 10 | 2019 | Offshore | BB |
| E79 | Seine | Strandir | NA | 0 | NA | 0 | Bassastadir | BAS-S | 65.8945 | -22.387 | 12 | 10 | 2019 | Offshore | AB |
| SF59BS8 | Seine | Isafjordur | 5 | 0 | 1.16 | 0 | Seydisfjordur | SEY-S | 65.95817 | -22.38883 | 5 | 9 | 2019 | Offshore | AA |
