# Supplementary material for: Genetic assignment predicts depth of benthic settlement for 0-group Atlantic cod
Source: PLoS One. 2023 Oct 4;18(10):e0292495. doi: 10.1371/journal.pone.0292495 (PMC10550133; doi:10.1371/journal.pone.0292495)
Supplement: S2 Table — (PDF) [file pone.0292495.s002.pdf]

S2 Table. Pairwise F_{ST} values based on the SNP dataset. Differences in the depth of benthic settlement of 0-group Atlantic cod of offshore and inshore origin. Comparisons between trawling and seining sites are indicated within a black frame. * indicates significance at the < 0.05 level and ** at the 0.01 level.

Corresponding author: gaol@hi.is

		Trawl sites			Seine sites						
		ISA	SKO	Outer_ARN	ARG_Aug	SEY_Sept	BAS_Aug	AUD_Oct	SEY_Oct	BAS_Oct	ARN_Sept
Trawl sites	Inner_ARN	-0.00200	0.01040	0.00949	0.14289**	0.07565**	0.07519**	0.11067**	0.08525**	0.05944**	0.10473**
	ISA	-	0.00931	0.01766*	0.12048**	0.06133**	0.06574**	0.09420**	0.06980**	0.04526**	0.09042**
	SKO	-	-	0.00797	0.19833**	0.12378**	0.12614**	0.16523**	0.13617**	0.10446**	0.15845**
	Outer_ARN	-	-	-	0.20741**	0.13077**	0.13401**	0.17429**	0.14236**	0.11869**	0.16849**
Seine sites	ARG_Aug	-	-	-	-	0.01328*	0.01306*	0.01124	0.01068	0.02967*	0.00906
	SEY_Sept	-	-	-	-	-	0.00197	-0.00089	-0.00249	0.00595	-0.00189
	BAS_Aug	-	-	-	-	-	-	0.00743	0.00019	0.01150*	0.00470
	AUD_Oct	-	-	-	-	-	-	-	-0.00219	0.00553	-0.00571
	SEY_Oct	-	-	-	-	-	-	-	-	0.00424	-0.00064
	BAS_Oct	-	-	-	-	-	-	-	-	-	0.00661
