# Supplementary figures and images for: Genetic assignment predicts depth of benthic settlement for 0-group Atlantic cod
Source: PLoS One. 2023 Oct 4;18(10):e0292495. doi: 10.1371/journal.pone.0292495 (PMC10550133; doi:10.1371/journal.pone.0292495)

# 1-year old juveniles

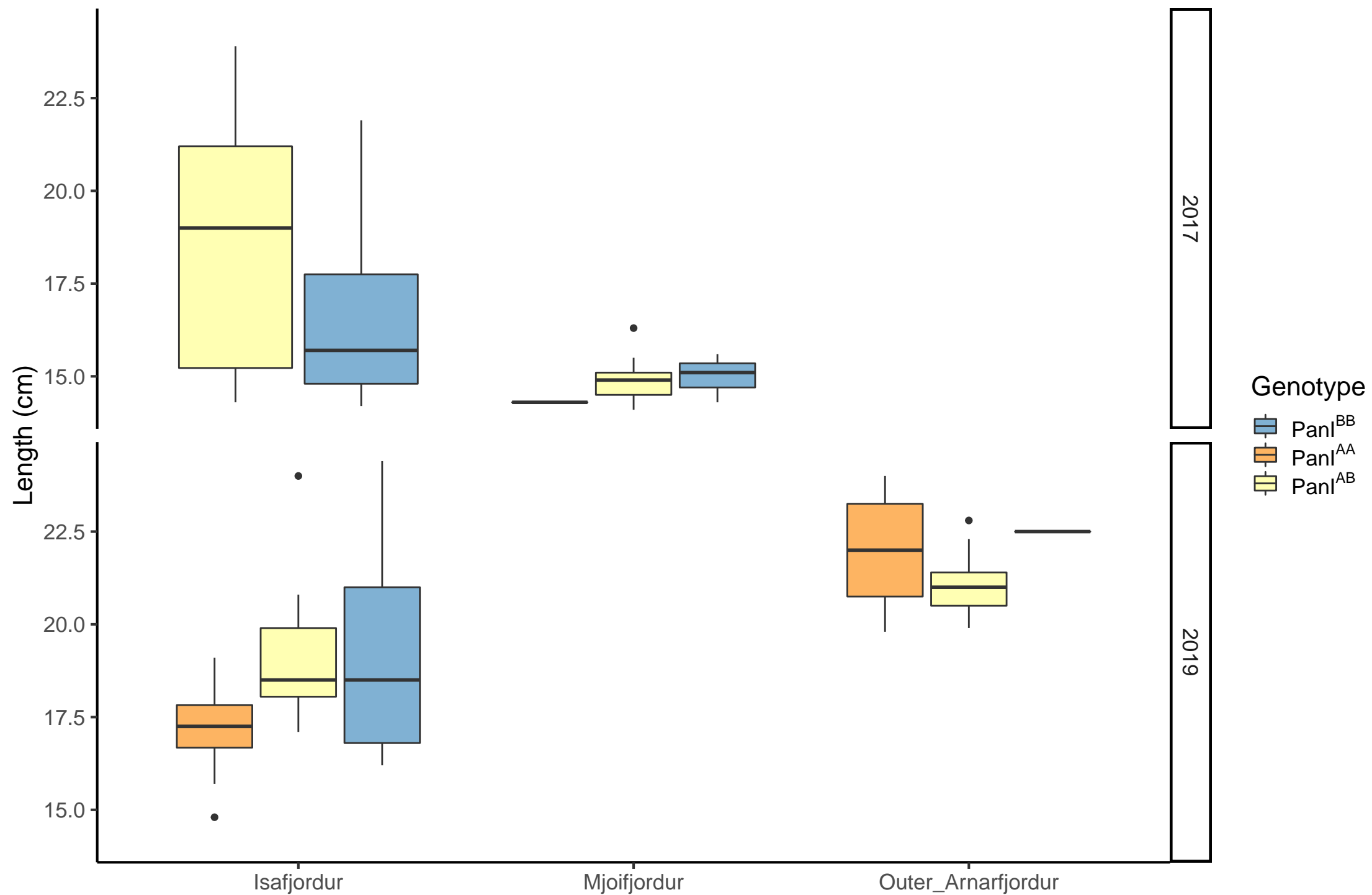

Supplement: S1 Fig — (PDF) [file pone.0292495.s003.pdf]

Year ■ 2017 ■ 2019

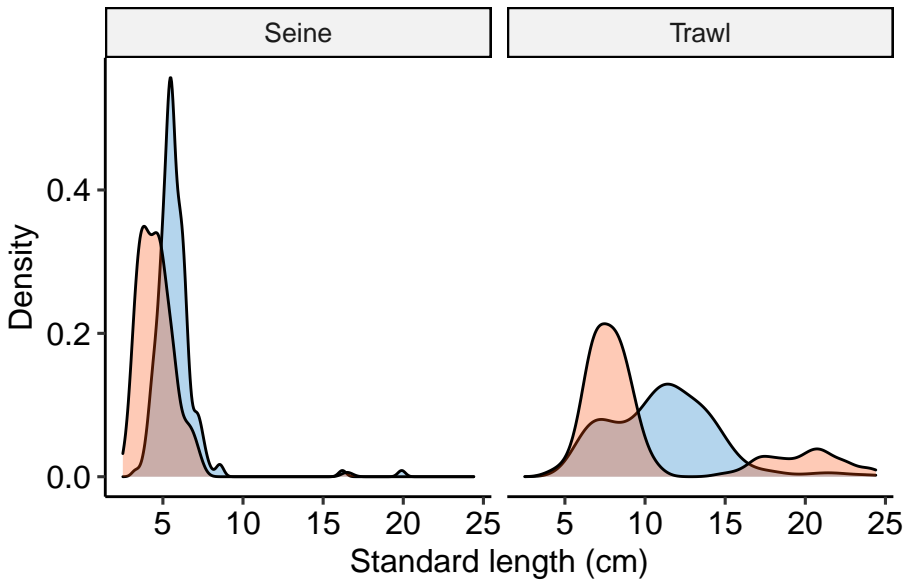

Supplement: S2 Fig — (PDF) [file pone.0292495.s004.pdf]
